# The inherent capacity of neurons to learn order relations and support abstract reasoning

**DOI:** 10.1038/s41467-026-76102-5

**Published:** 2026-08-05

**Authors:** Yukun Yang, Wolfgang Maass

**Affiliations:** Institute of Machine Learning and Neural Computation, Graz University of Technology, Graz, Austria

**Keywords:** Network models, Learning algorithms, Electrical and electronic engineering, Decision

## Abstract

Brains extract relations between objects and concepts and integrate them into cognitive maps for decision-making. But it remains unclear how they achieve that. Here we present a rigorous theory showing that single neurons can already learn to extract ranks of items in a linear order with a simple local rule for synaptic plasticity. The resulting model explains human brain data on the emergence of cognitive maps from linear orders, accounts for the terminal item effect in transitive inference, and enables rapid reconfiguration of internal representations when new evidence appears. We also present a theoretical explanation for the surprising fact that 2D projections of neural representations of linear orders in the brain are curved rather than linear. Since the model requires only local synaptic plasticity in shallow networks, it is suited for relational learning and fast inference on low-energy edge devices. We demonstrate this on the neuromorphic chip Loihi 2.

## Abstract (web summary)

How the brain learns relations between objects and concepts remains largely unclear. Here, the authors show that a simple synaptic plasticity rule can achieve this and enable abstract inference, reconfiguration, and the emergence of cognitive maps in shallow networks.

## Introduction

A hallmark of higher cognitive function is the capability to extract and represent information about relations between objects in a visual scenes, or between abstract concept, to integrate information from several learnt relationships, and to use it for fast inference. In particular, the brain is able to extract information about order relations between items. Furthermore, it encodes this order information in a way that supports transitive inference, a key paradigm for abstract reasoning. Essential for transitive inference is the extraction and neural representation of a latent variable from items in an order relation that can not be directly observed: their rank. Experimental data show that brains are able to extract this latent variable from dispersed information about order relations between pairs of items, and to encode it in the parietal cortex. In fact, experiments show that rank information is encoded by the brain in two different ways: By a summation code where many neurons fire with a rate that is indicative of the rank of an item, and by neurons that only fire for a single rank and a few adjacent ranks (heterogeneous code), see the review in ref. ^1^. Experimental evidence for the existence of a summation code was provided by refs. ^2–5^, see section 6 of the review^1^ and Box 3 in ref. ^6^ for an overview. The summation code was explained in ref. ^2^ from a more general perspective: The surprising dominance of a single dimension in the network dynamics of area LIP (lateral intraparietal cortex). This effect is consistent with a large body of experimental data on the representation of numbers and other types of magnitudes in the brain^1,6–14^. Informally one refers to these common types of 1-D neural representations as mental number line and as a 1-D cognitive map^6^. It is closely related to the theoretically postulated geometric mental line of ref. ^15^. One functional advantage of such a scalar neural network code is that downstream network can use the same decoder for many tasks^3^.

The presence of a summation code for ranks of items in an order makes transitive inference easy: Downstream networks just have to compare the firing activity of these neurons for two different items in an order. In fact, this computational strategy automatically gives rise to the symbolic distance effect that is seen in human behavior, see the review in ref. ^16^: Human subjects have lower reaction time and make fewer errors if the required transitive inference involves pairs of items whose ranks are substantially different. But a summation code does not explain the terminal item effect, also referred to as serial position effect^16–18^, whereby transitive inference that involves a terminal item in an order exhibits fewer errors and is carried out faster than for other pairs of items with the same rank difference.

It remains unknown by which learning process the underlying latent variable of an order, the rank of an item, is extracted from dispersed pieces of information about order relations between pairs of items and mapped to the largely 1-dimensional dynamics of area LIP. Previous models for learning order relations in the brain involved multi-layer perceptrons^19^ or recurrent neural networks^20^ that were trained through stochastic gradient descent, or involved a special-purpose neuromodulatory system that had been optimized for this task^21^. An exception is the approach of ref. ^15^, where also a shallow linear network is used for learning orders. They considered the case where all order relations between pairs of items that are presented during learning are guaranteed to be adjacent items in the order, and observed that learning of an order can be reduced in this case to solving a linear regression task. Achieving through learning a representation of ranks in neural networks with a basically 1-dimensional dynamics, like that of area LIP, has been addressed besides ref. ^15^ only with the multilayer neural network model of ref. ^19^, where the activation of neurons on a hidden layer exhibited a 1D dynamics after Backprop training.

We present a minimal model for learning to extract and represent ranks in the area LIP that is not only consistent with a large body of experimental data, but is also supported by a rigorous mathematical theory. The simplest possible assumption about the underlying network architecture is to have direct synaptic connections between neural populations that represent items and a neural system that has an LIP-like 1-dimensional dynamics, as in ref. ^2^, or even a single linear neuron. We show that this simple architecture suffices for learning rank estimates, with a simple and biologically plausible rule for synaptic plasticity. Furthermore, this learning capability is supported by a rigorous mathematical theory, the Rank Convergence Theorem. Our simple model exploits the commonly observed fact that different objects and concepts tend to be represented in higher brain areas by approximately orthogonal neural codes. For example, it was found that different concepts or items are represented in the medial temporal lobe (MTL) of the human brain through the firing of almost disjoint assemblies of concept cells^22^, i.e., by approximately orthogonal high-dimensional (high-D) neural codes. Approximate orthogonality of neural codes was also found in the hippocampus of the rodent^23^. Orthogonality of item representations, or even one-hot encoding, has also been commonly assumed in other models for order learning, see, e.g., ref. ^19^.

We then address experimental data which show how the brain integrates information from different learnt order relations into a cognitive map^24^, or into a larger order^19^. We are not aware of any model for the former, and for the latter only of a multi-layer perceptron model trained through stochastic gradient descent with different learning rates for each pair^19^ and of a RNN model trained through stochastic gradient descent with an artificial neuromodulatory system and meta-training^21^. We show that our approach provides simple and transparent models for both of these order integration tasks.

Apart from modeling how the brain learns and represents order relations, it is also important to understand how such learning processes can be implemented in energy-efficient neuromorphic hardware with simple on-chip learning rules. Whereas previous models based on training multi-layer networks by backprop were not suitable for that, our simple model is suitable for implementation through on-chip learning with memristors that can assume a fair number of resistance values, such as the ones described in ref. ^25^. In addition, digital neuromorphic platforms such as Loihi2^26^ are also suitable because they support programmable neuron dynamics, event-driven computation, and local on-chip plasticity. Furthermore, autonomous generation of cognitive maps from several learnt order relations as we show for the data of ref. ^24^ from the human brain, appears to be useful for enabling navigation and abstract inference in robots.

In the last section of the paper, we address another important result on neural representations of rank representations in the parietal cortex of the human brain. Although the primarily 1-dimensional dynamics of area LIP suggests that 2D projections of fMRI recordings of rank representations should lie on a line, they are consistently curved. The question why this is the case was explicitly raised in ref. ^19^. We provide a rigorous explanation of this effect based on the fact that ranks are not only represented in a summation code, but also by heterogeneous rank selective neurons. We show that this representation entails that 2D projections of neural codes for ranks lie on a curve, rather than on a line. The same theory also explains other questions about curved 2D projections of high-D neural codes that were addressed in ref. ^27^.

## Results

### Order relations can be learnt by single neurons

We want to understand how neurons in area LIP can learn to produce rank estimates for items **a**_*i*_ in an order “ ≺ ” that are each encoded by some high-D input vector. We assume that information about this order “≺” is provided in a realistic piecemeal fashion, through unstructured sequences of order relations **a**_*i*_ ≺ **a**_*j*_ for arbitrary pairs of items (that are not necessarily adjacent in the order). Producing rank estimates for items in this order is a non-trivial computing and learning task, because the rank of an item **a**_*i*_ is a latent variable that depends in a complex way not only on order relations that involve this item **a**_*i*_, but also on order relations for all other pairs of items.

**Fig. 1:**
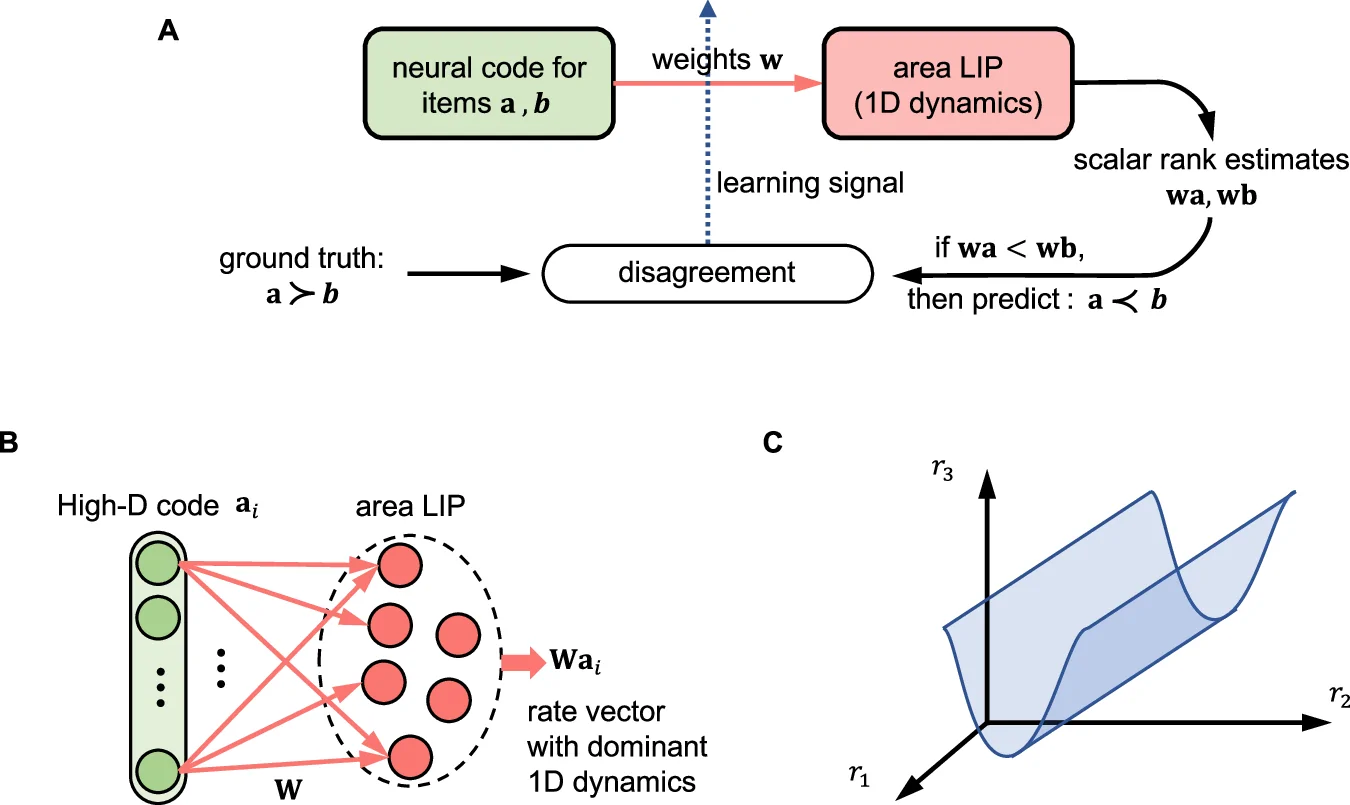
Architecture of our model. **A** The underlying online learning framework: Weights are updated whenever the model produces through its rank estimates *r*(**a**) and *r*(**b**) predictions about the order between two items **a** and **b** that turn out to be wrong. **B** Proposed architecture for learning rank estimates (summation code) in area LIP: High-D representations of items are projected through synapses with weights **w** onto a population of neurons that has an ~1-D dynamics. **C** Scheme for 1-D dynamics of firing rates in a network: Activity states of the network drawn to a 1-D attractor a suggested by ref. ^2^ (compare with their Fig. 2).

This learning process can best be modeled as an online learning process, instead of a batch learning process that is more common in machine learning, but also in models for learning order relations. In online learning, weight updates are only carried out upon errors or surprises, i.e., when information about a relationship **a**_*i*_ ≺ **a**_*j*_ disagrees with current internal rank estimates, see Fig. 1A. The number of these errors is a standard measure for the efficiency of an online learning algorithm. Our model can also be trained using batch learning, and the standard measure for the efficiency of that, the number of examples that are presented-yields roughly twice the number of weight updates observed in online learning, see Section B.2 of the Supplementary Information.

We show that this online learning process can be accomplished by a single layer of synapses with weights **W** to area LIP, as indicated in Fig. 1B. In other words, we show that after learning the overall firing activity **r**(**a**_*i*_) (space rate code) in a population of neurons that is targeted by these synaptic connections is able to provide consistent approximations of the ranks of all item **a**_*i*_ in a learned linear order “ ≺ ”.

**Fig. 2:**
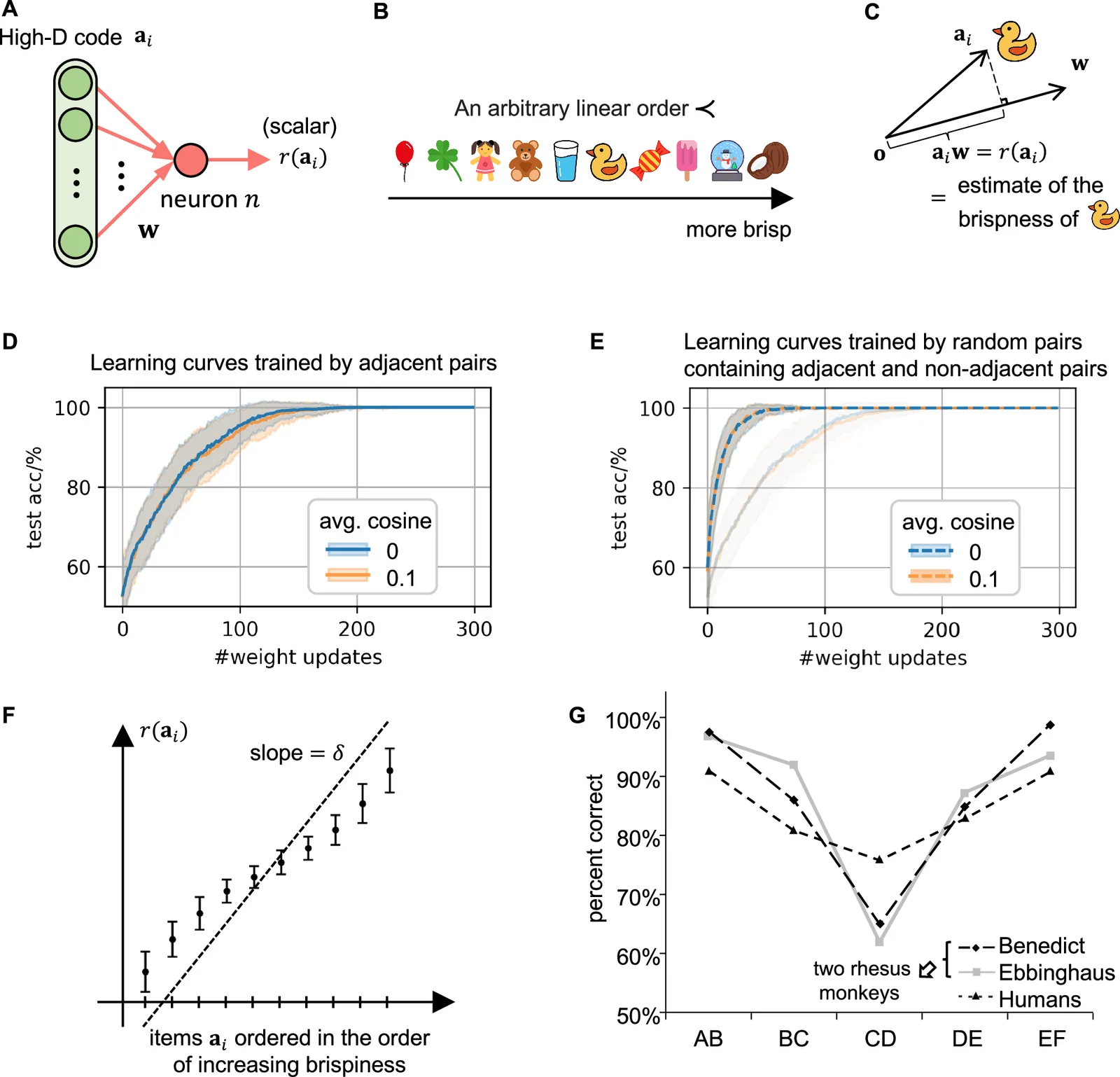
Performance of the single neuron model, and comparison with brain data. **A** Analytically tractable version of the model in panel B of the previous figure, with a single linear neuron representing the firing activity of a population with 1-dimensional dynamics. **B** Task illustration: Learning the relation “more brisp than" for 10 items. **C** The brispiness prediction for an item **a**_*i*_ (here: a duck) by the linear neuron in panel D can be understood geometrically as the length of the projection of the high-D neural code for **a**_*i*_ onto the weight vector **w**. Learning **w** therefore amounts to finding a direction where the projection lengths *r*(**a**_*i*_) are consistent with the brispiness relations among items. **D** Emergence of transitive inference capability. Accuracy was evaluated after each weight update on non-adjacent item pairs, although the learner only received information about adjacent pairs. Perfect orthogonality of item representations **a**_*i*_ was assumed for the blue curve, and approximate orthogonality, suggested by experimental data, with an average cosine of 0.1 for the orange curve. The model therefore does not require perfect orthogonality for fast learning. Means and standard deviations were calculated over 100 training rounds with different random seeds. **E** Same settings as in panel D except that all pairs, rather than only adjacent pairs, were used as the training set. Results from panel D are shown with higher transparency in the background for comparison. **F** Brispiness predictions for all items were evaluated at the stage when they first became accurate, which took on average 93.6 weight updates. Dots and error bars indicate means and standard deviations, respectively, over 100 training rounds with different random seeds. The resulting rank estimates show a characteristic S-shape. **G** Experimental data from ref. ^17^ for two monkeys ("Benedict" and “Ebbinghaus") and human subjects on the terminal item effect. Order estimates were more accurate for pairs involving terminal items, as predicted by the S-shape in panel F.

According to the experimental data that we reviewed in the Introduction, the neural dynamics in area LIP is dominated by a 1-D line attractor (see Fig. 1C), that can be reproduced in a model through lateral connections between excitatory neurons^2^. In order to make the learning process analytically tractable, we replace this population with 1-D dynamics as shown in Fig. 1B by a single linear neuron *n*, see Fig. 2A, that provides rank estimates for items **a**_*i*_ through its output *r*(**a**_*i*_) = **wa**_*i*_. One can interpret it as the firing rate of neuron *n* relative to a non-zero baseline firing rate, in order to account for negative values that it may assume.

We apply the following simple learning rule for the weights of this neuron, to which we refer in the following as Rank Learning Rule:

#### **Rank Learning Rule**

Define an error signal *e*_*ij*_ that assumes value 1 if information is received that **a**_*i*_ ≺ **a**_*j*_ for some items **a**_*i*_ and **a**_*j*_, but *r*(**a**_*j*_) = **wa**_*j*_ is not by some given margin *δ* larger than *r*(**a**_*i*_) = **wa**_*i*_; else *e*_*ij*_ = 0. The Rank Learning Rule is:

1 $${{{\bf{w}}}}\,\leftarrow \,{{{\bf{w}}}}+\eta \,{e}_{ij}\,({{{{\bf{a}}}}}_{j}-{{{{\bf{a}}}}}_{i}).$$

The parameters *δ* and *η* have fixed positive values, representing the margin and learning rate, respectively. In our experiment, the default values are *δ* = 1 and *η* = 0.1.

This learning rule is arguably the simplest and most straightforward one because it arises from gradient descent for the ranks of two fixed items **a**_*i*_ and **a**_*j*_, see Section A.1 of the Supplementary Information. Hence, if it is sufficiently often iterated, it will eventually achieve that *r*(**a**_*i*_) + *δ* < *r*(**a**_*j*_). But updates for this pair of items are in general interwoven with updates for other pairs, which may contain some of the same items or not. Hence it is not a-priori clear whether this simple learning rule converges, i.e., arrives at rank estimates that are consistent with all order information that has been received. For example, the weight updates might get caught in infinite loops, since an item **a**_*i*_ has to satisfy many relations **a**_*i*_ ≺ **a**_*k*_ and **a**_*l*_ ≺ **a**_*i*_ with other items **a**_*k*_, **a**_*l*_. As long as the rank of **a**_*i*_ is not yet properly reflected in the output value of *n* for synaptic input **a**_*i*_, the Rank Learning Rule may sometimes add a fraction of **a**_*i*_ to **w** and sometimes subtract a fraction of it. These updates could potentially cancel each other. We show in Fig. 2B, C that the Rank Learning Rule works very well for learning the nonsensical relation “more brisp than” for 10 items (borrowed from ref. ^19^). For example, the item “candy” is more brisp than the item “yellow duck”. The neuron learns from a stream of information that one item is more brisp than some other item, and updates its internal model for predicting brispiness when needed, as indicated in Fig. 1A. The learning performance is shown in Fig. 2D. After, on average, 93.6 updates it produces rank estimates as shown in Fig. 2F that are consistent with the given order.

By design, the main experiments in Fig. 2D restrict training to adjacent pairs and evaluate the learned ranks on non-adjacent pairs. This setting provides a direct test of transitive inference, since the model has to infer long-range order relations that were never presented during training. We further show in Fig. 2E that the Rank Learning Rule also learns correct ranks when order relations between randomly chosen pairs are provided during learning, including non-adjacent pairs. The faster convergence in this setting is expected, because long-range relations are then observed directly during training and the training and test pair distributions overlap. Thus, this experiment serves as a complementary control, whereas the adjacent-pair setting provides the main test of transitive inference. A more detailed analysis is provided in Supplementary Information Section B.3.

Transitive inference can be carried out by downstream networks in a straightforward manner on the basis of rank estimates that are encoded by firing rates in area LIP: In order to determine which of two items comes later in the order they just have to evaluate which of them elicits a higher firing rate. This model is consistent with the commonly observed “symbolic distance effect”^28^ in the performance of transitive inference by animals and human subjects: The accuracy of transitive inference increases, and the response time decreases, with the distance of two items in a learned order. Our model suggests that this effect arises because the comparison of two firing rates by downstream networks takes less time and is more reliable if the two firing rates are further apart. In order to demonstrate this rigorously, one has to transform the rank estimates of our model into rates of Poisson spike trains, and analyze the time it takes to compare the firing rates for two adjacent ranks with that for two items with a larger rank difference.

The concrete rank estimates that emerge through the Rank Learning Rule, shown in Fig. 2F, provide an explanation for another commonly observed effect in transitive inference by human subjects: The “terminal effect” or “serial position effect”^16–18^. This effect describes the fact that transitive inference for pairs of items in a learned order has higher accuracy and requires less response time, compared with other pairs of items that have the same rank distance, if one of the two items is an endpoint in the learned order. For illustration, experimental data from ref. ^17^ are shown in Fig. 2G). The subjects had learnt order relations between 6 images, labeled A, …, F through presentations of image pairs with adjacent labels, and were subsequently tested on image pairs with adjacent labels. One clearly sees better performance for pairs at the two ends of the linear order, This effect can be explained by the S-shape of the firing rates that emerge through learning with the Rank Learning Rule according to Fig. 2F: Firing rates for items at the endpoints of the learned order relation are further apart from those for next-ranked items, compared with distances between firing rates for other pairs of items that have the same rank difference. This non-linearity arises from the fact that the rank estimates for items at the endpoints are only pushed towards the outside by the Rank Learning Rule, whereas items in the mid-range are subject to numerous conflicting updates that push their rank estimates up and down.

The Rank Learning Rule works best if the items **a**_*i*_ are encoded by orthogonal vectors. Orthogonal codes have recently been found to arise commonly in the rodent hippocampus^23^. But experimental data suggest that visual objects and concepts are represented also in the human brain by approximately orthogonal neural codes: Through sparse assemblies of “concept cells” in the MTL^22^. If one encodes these assemblies by binary vectors, where a “1” indicates that a neuron belongs to this assembly, these experimental data support a model where items are encoded by sparse high-D binary vectors. These vectors can not be assumed to be perfectly orthogonal, since assemblies for different concepts are known to have an overlap between 1% and 4%, depending on the degree by which the underlying concepts are associated^29^. Figure S2A shows that this amount of overlap between assemblies amounts to a cosine similarity between 0.02 and 0.08 for the corresponding binary vectors. The orange curves in Fig. 2D, E, and also in later sections show that the learning performance is still very good for an average pairwise cosine value of 0.1 between vectors that represent different items. In other words, the level of orthogonality that is needed for the Rank Learning Rule is reached by the approximate orthogonality that has been found in neural codes for concepts in the MTL of the human brain, no matter whether they are associated or not.

We are not aware of experimental data which elucidate whether a transitive inference query “is **a**_*i*_ ≺ **a**_*j*_?” is processed by neural circuits of the brain by estimating the ranks of these two items in a sequential manner, or whether these items are provided simultaneously as input to these neural circuits. Similarly, it is not clear whether order information **a**_*i*_ ≺ **a**_*j*_ is processed during learning by placing attention sequentially on each of the two items, or in a fully parallel manner. The sequential process can be supported by the argument that placing attention simultaneously on two different items appears to be difficult, at least without explicit training. Our model is neutral with regard to this issue, since it can also be used for fully parallel computations: By giving neural codes for both items simultaneously as inputs to the linear neuron, but **a**_*j*_ with factor  + 1 and **a**_*i*_ with factor  −1, as in the models of refs. ^15,19^. After learning, the linear neuron will then output the rank difference of both items, and this value is positive if and only if the answer to the question “is **a**_*i*_ ≺ **a**_*j*_?” is “yes”. Note that the Rank Learning Rule is also compatible with this fully parallel model, since the weight update is then proportional to the total input **a**_*j*_ −**a**_*i*_ that the neuron receives.

We show in the section “Proof of the Rank Convergence Theorem and details of experiments, discussed in section ‘Learning theory for the Rank Learning Rule’” that the Rank Learning Rule is closely related to the Perceptron Learning Rule for learning a classification of differences **a**_*j*_ −**a**_*i*_. Hence, models for a biologically plausible implementation of the Perceptron Learning Rule via STDP, see ref. ^30^, also provide a biologically plausible implementation of the Rank Learning Rule. Other closely related learning rules that are commonly viewed as being biologically plausible are the Widrow-Hoff rule and the Rescorla Wagner rule^31^. The weight dynamics under the Rank Learning Rule can intuitively be understood as follows: The weight vector moves toward the higher ranked input vector and away from the lower ranked input vector. This is somewhat analogous to the competitive learning of neurons in a WTA circuit, as discussed by ref. ^32^, Ch. 5, where each weight vector moves toward the cluster of inputs that the corresponding neuron will represent.

Figure 2D, E show that learning rank estimates for brispiness works very well in our simple model. But it is not clear whether this example is representative. A deeper analysis of the dynamics of the learning process is needed in order to determine whether it converges for any given order, and for arbitrary sequences of pieces of information that inform about the order relations between some pairs of items. This analysis is provided in the following section.

### Learning theory for the Rank Learning Rule

A simple geometrical argument shows that there exists for any order and any encoding of items by orthogonal vectors a weight vector **w** which enables the neuron *n* to produce accurate rank estimates for all *N* items in the order. Fig. 2C suggests that it suffices to make sure that each item has a projection of a suitable length onto the vector **w**. Hence one can simply define it for any correct set of ranks *r*_*i*_ for the items **a**_*i*_ as

2 $${{{\bf{w}}}}=\sum_{i=1}^{N}{r}_{i}{{{{\bf{a}}}}}_{i}.$$

But this simple argument, assumes that one has already correct rank estimates *r*_*i*_, although these are not provided directly during the learning process. Furthermore, it does not entail that the Rank Learning Rule converges to a vector **w** that provides correct rank estimates. It had been shown in ref. ^15^ that for the special case where only pairs of items are presented during learning that are adjacent in the order that is to be learnt, learning of the order can be reduced to a linear regression task. The optimal solution can be approximated via gradient descent, where the gradient descent rule is identical to the Rank Learning Rule, except that it requires a varying continuous-valued factor that measures continuous-valued deviations from target values. In contrast, we show that the Rank Learning Rule reaches after finitely many steps an optimal solution with a fixed learning rate. Importantly, it learns an order also if non-adjacent pairs of items are presented during learning. This can not be achieved if order learning is formulated as regression task, because one would then have to provide the actual rank difference of the two items, which becomes only known after the order has been learnt.

The learning theory for the Rank Learning Rule is governed by a rigorous mathematical result, the Rank Convergence Theorem. This Theorem guarantees that the Rank Learning Rule converges for every given total order “ ≺ ”, and for any order of presentations of pairs of items during learning, after finitely many weight updates. The precise statement of the Rank Convergence Theorem (see “Methods”) is analogous to that of the famous Perceptron Convergence Theorem, which guarantees convergence of online learning of binary classifications with a simple plasticity rule (the delta learning rule). But the Rank Learning Rule has to solve a different task, since it has to learn to output estimates of a latent variable, the rank of an item, rather than a binary classifications. One can prove the Rank Convergence Theorem directly. For the case where the margin *δ* has value 0 one can also reduce it to the proof of the Perceptron Convergence Theorem. Both lines of argument are presented in the “Methods” section.

Note that the Rank Convergence Theorem only guarantees that if one keeps on presenting pairs of items for which the estimated ranks are not yet consistent with their order relation, this process will end after finitely many steps with a weight vector **w** for which the rank estimates that neuron *n* provides are fully consistent with the order. But if one stops this learning process prematurely, the rank estimates that neuron *n* provides are in general not yet consistent with all order relations.

There may even be cases where the order relations between some pairs of items are unknown, e.g. when a partial order is to be learned. In this case the Rank Convergence Theorem guarantees that the learning process converges to rank estimates that are consistent with all pairwise order relations that exist in the partial order. In order to apply the Rank Convergence Theorem, which assumes existence of a total order that is consistent with all examples, one can use the fact that every partial order can be embedded into a total order.

An empirical finding is that the inference of new relations between items-those implied by other previously revealed relationships-reaches high accuracy quite fast (see Fig. 2D, E for example). The y-axes of these plots illustrate the emergent correctness of these inferences during learning. Furthermore, as detailed in section “Rapid reconfiguration of the internal model when separately learned orders are combined on the basis of new information,” our model learns approximately 7-24 × faster than humans in one experiment and 10 × faster in another one under identical settings. This may be due to the presence of noise in neural circuits of the human brain, or to rules for synaptic plasticity that require more iterations. But one can adjust our learning model to these behavioral data by lowering its learning rate.

### Neuromorphic implementation

Our model is well suited for neuromorphic hardware because the Rank Learning Rule is local, event driven, and shallow. It only requires pre-synaptic presentation of two items and a signal that informs about the true order between them. Hence learning does not require backpropagation, weight transport, multi-layer credit assignment, or high-precision gradient accumulation.

**Fig. 3:**
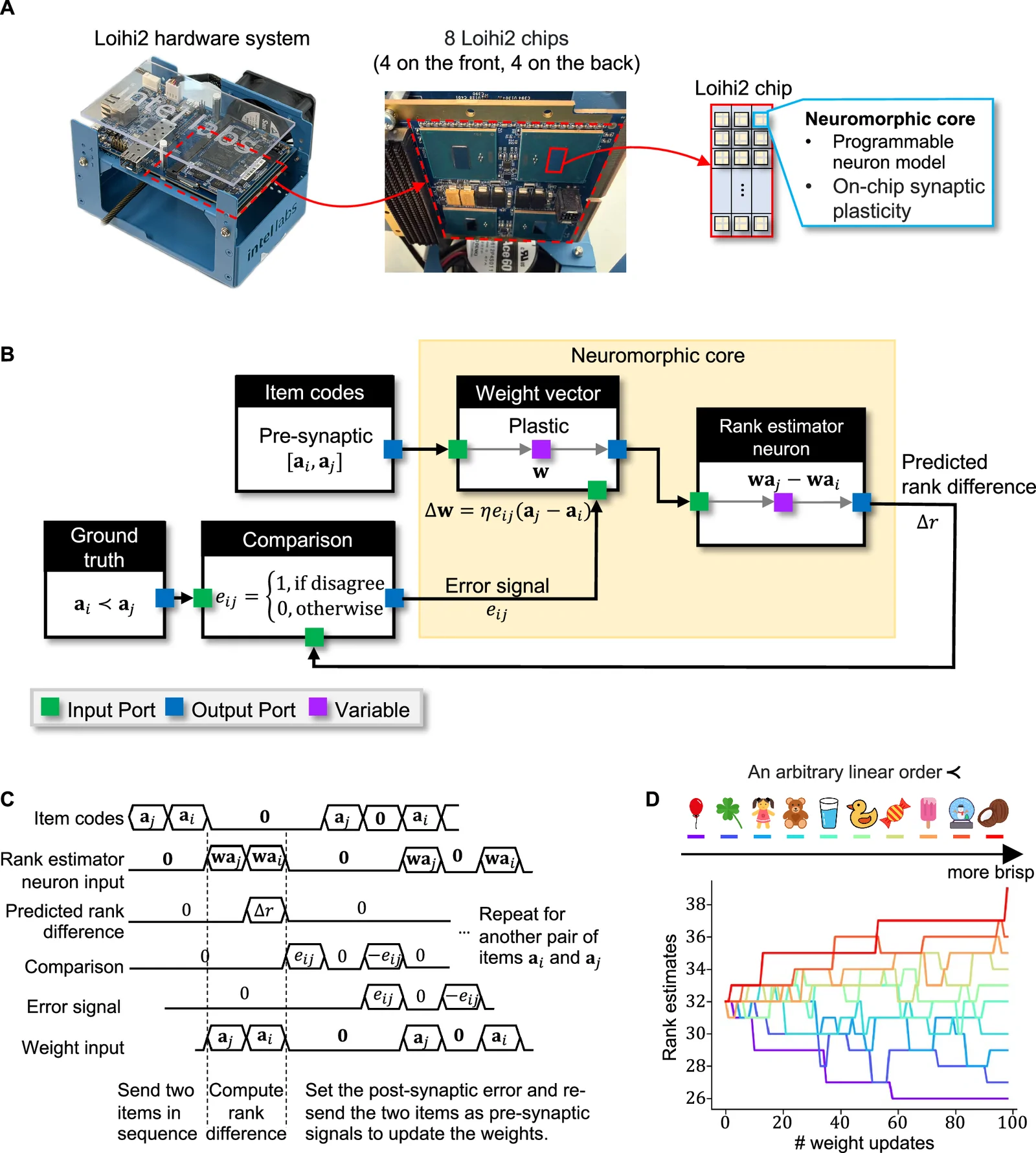
Neuromorphic implementation of the Rank Learning Rule on Loihi2. **A** Target platform. The model is deployed on a Loihi2 hardware system, whose neuromorphic cores support programmable neuron dynamics and on-chip plasticity. **B** Network architecture. Sequential item inputs [**a**_*i*_, **a**_*j*_] and a signed teaching signal *e*_*ij*_ are delivered via separate input groups. A linear plastic neuron computes the predicted rank difference **wa**_*i*_ − **wa**_*j*_, and synaptic weights are updated locally according to *Δw* = *ηe*_*ij*_(**a**_*i*_ − **a**_*j*_). The rule depends only on pre-synaptic signals and a locally computed error. **C** Micro-time schedule. The first item is stored in the internal state, the second item is used to create the difference signal, and the ground truth is injected in a controlled time window before re-presenting the pair to trigger weight modification. This makes explicit how temporary storage, error timing, and local learning can be implemented in neuromorphic hardware. **D** Learning outcome on hardware. Rank estimates gradually separate with each update and converge to the ground truth order learned from random adjacent and non-adjacent pairs. Each training pair triggers two updates (see panel C), so fewer than 50 pairs are needed to learn the correct order.

A neuromorphic implementation is illustrated in Fig. 3. Figure 3A shows the target hardware platform, a Loihi2 system with programmable neuron dynamics and local synaptic plasticity. Fig. 3B shows how the learning architecture can be mapped onto Loihi2’s Neuromorphic core. Sequential item inputs [**a**_*i*_, **a**_*j*_] are delivered through one pathway, while a ground truth signal is provided through another. A linear plastic neuron computes the signed rank difference between the two items, and synaptic weights are updated only when the represented order is not yet correct.

Figure 3 C shows how this computation can be distributed over a short micro-time schedule. The first item **a**_*j*_ is presented and its estimated rank **wa**_*j*_ is stored in an internal state, the second item **a**_*i*_ is then used to form the signed rank difference signal **wa**_*j*_ −**wa**_*i*_. The locally computed error signal is injected in separate time steps while both items are re-presented to trigger synaptic modification. Thus temporary storage, error gating, and plasticity can all be realized through local state variables and event-based control. Fig. 3D shows the resulting learning dynamics on hardware, where rank estimates separate over updates and converge to the correct linear order, learning the “more brisp than” order introduced in Fig. 2B.

An additional advantage is that the hardware implementation does not require rate-coded real-valued inputs. Item codes can be translated into spike trains or binary event patterns, and the error variable only needs a small discrete alphabet. If pairs are always presented in a fixed order, a binary error signal {0, 1} indicating if the learned order agree with the ground truth is sufficient. If the presentation order is randomized, a ternary signal { + 1, 0, −1} suffices.

This simple implementation on Loihi2 shows that the proposed model is compatible with the main principles of neuromorphic computation. Since the learned variable **wa**_*i*_ is a scalar rank estimate, the resulting circuit can directly support downstream comparison, transitive inference, and other forms of fast relational reasoning. The same implementation principle also extends naturally to the context-dependent version of the model in section “Rapid reconfiguration of the internal model when separately learned orders are combined on the basis of new information,” where separate synaptic pathways for item and context information remain compatible with local learning.

### Emergence of cognitive maps from several learnt order relationships

**Fig. 4:**
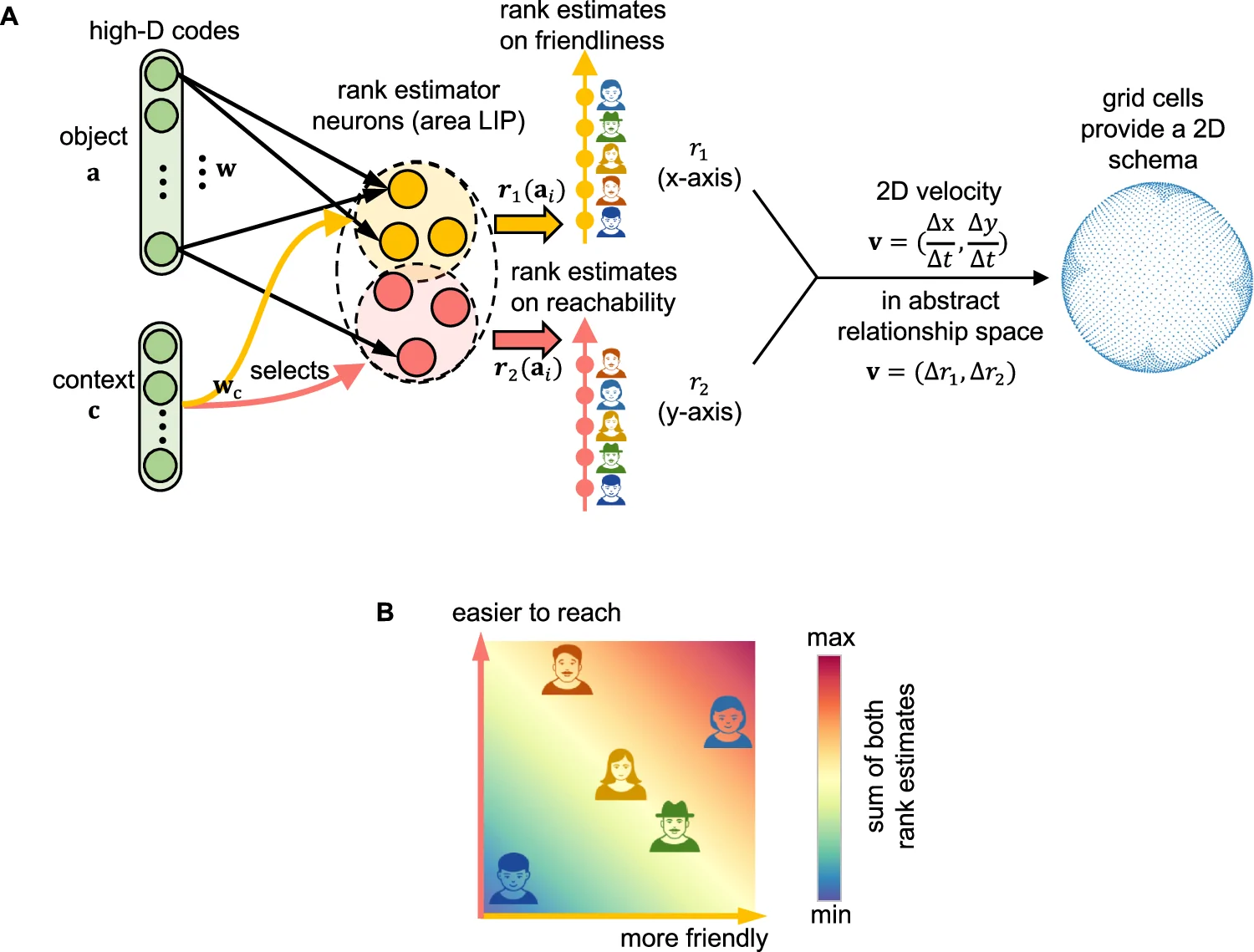
Emergence of a cognitive map from two learnt order relations. **A** Rank estimation is computed as the sum of inputs from object coding (weights **w**) and from context coding (weights **w**_*c*_). The context code can then select subgroups of neurons to respond to a specific context by inhibiting the others. This yields parallel representations of ranks in two different orders by different neurons. One can treat the outputs of these neurons like velocity signals from area EC in a navigation task^34^, and feed them into a grid cell network that creates from them a 2D cognitive map. Possible responses of grid cells can be plotted as locations on a torus^54^. The 2D schema shown here is the PCA result of grid cells' activation for 50 × 50 mesh-grid locations in a small square that corresponds to an approximately flat fragment of the surface of the torus. **B** An example of a 2D cognitive map that enables inference about five people in two relations (friendliness and reachability).

In the real world, an item may participate in multiple order relations. For example, we learn that some people are more friendly than others, and independently, that also some of them are easier to reach than others, see Fig. 4A for an illustration. The recent study^24^ shows through neural recordings from the human brain that it integrates information from two learnt orders into a 2D cognitive map. Compared with transitive inference for a single order, this cognitive map enables a much larger repertoire of abstract inference tasks that involve both orders. We show that a transparent model for these experimental data arises if one duplicates our base model for learning two linear orders so that the firing rates of two different neurons (or two populations of neurons) in area LIP provide rank estimates for an item in the two orders. Integration of information from two different contexts in area LIP had already been examined in ref. ^33^. We capture that by letting context inputs gate off irrelevant neurons via strong inhibition through **w**_*c*_, see Fig. 4A. Routing the outputs of neurons that report rank estimates for the two linear orders to a standard model for a grid cell system from ref. ^34^ yields a 2D map that supports navigation and complex inference by combining information from both orders. The vector of the two rank estimates plays here a role that is analogous to the 2D velocity vector that provides for 2D navigation input to grid cells in the entorhinal cortex (EC)^34^. The resulting cognitive map, visualized by performing PCA on grid cell activations, can be found in Supplementary Information Section C.

To see how the resulting cognitive map enables inference that combines information from both order relations, we consider in Fig. 4B the query “Who is easiest to reach and most friendly?”. Within our model, this reduces to identifying the item closest to the top right corner of the cognitive map. But the geometry of this cognitive map also supports completely different inference tasks, such as finding the person that has the largest mismatch between friendliness and reachability.

### Rapid reconfiguration of the internal model when separately learned orders are combined on the basis of new information

**Fig. 5:**
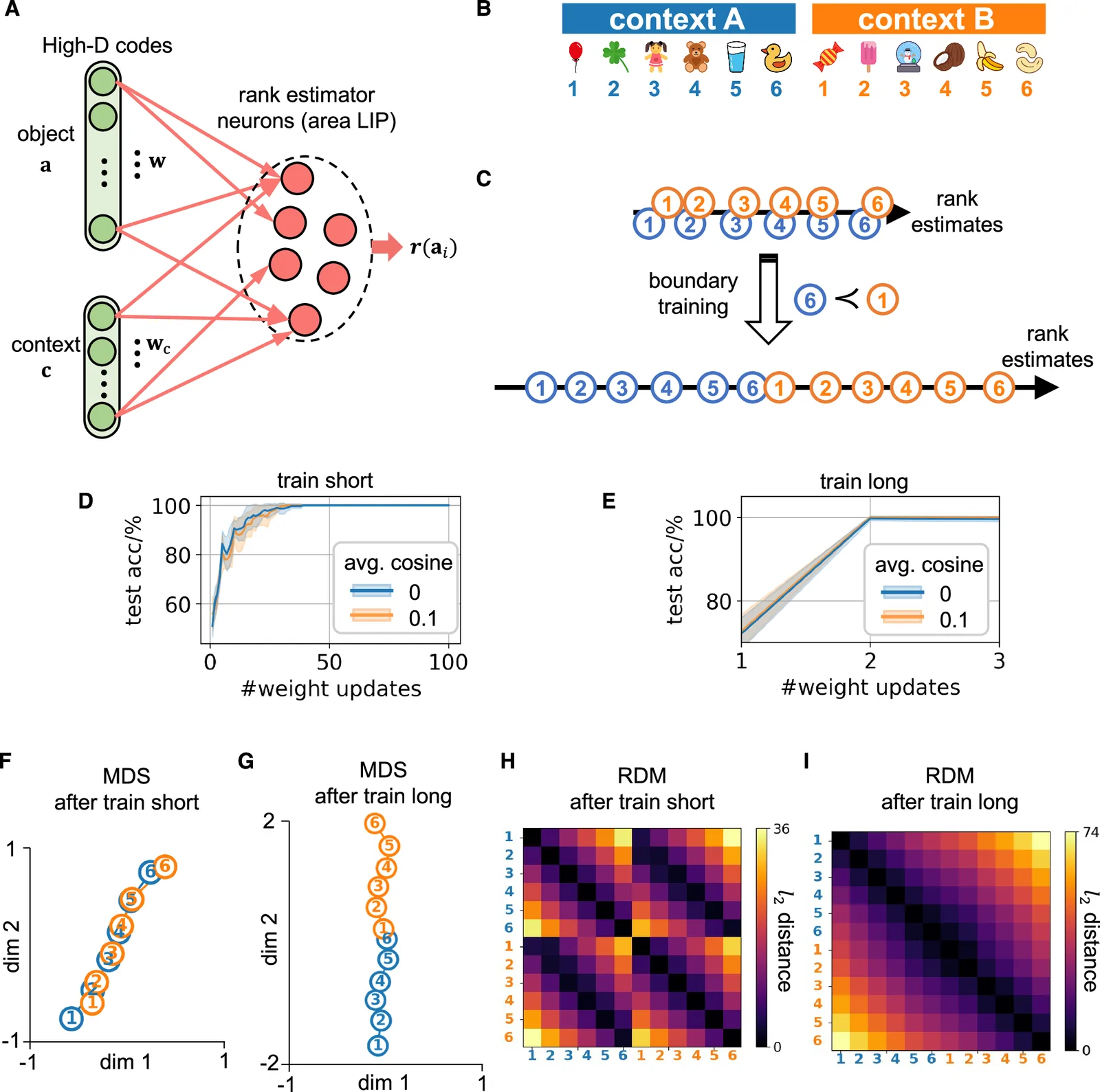
Combining information from separately learned orders. **A** Network architecture: Rank estimation is given by the sum of inputs from object coding (weights **w**) and from context coding (weights **w**_*c*_). **B** Task illustration: Two linear orders are learned separately in different contexts A and B during phase 1. Following ref. ^19^, we call this phase “train short". **C** In the 2nd learning phase (or “train long" following ref. ^19^), the information is provided that the item with lowest rank in context B comes after the item with the highest rank in context A ("boundary pair"). Resulting rank estimates of our model after train short and train long are shown. **D** Learning curve of the 1^st^ learning phase “train short". Model accuracy was evaluated after each weight update on all item pairs within the same context, while the learner received information only about the adjacent pairs within each context. **E** Learning curve of the 2^nd^ learning phase “train long". Model accuracy was evaluated after each weight update on all item pairs in the combined order, while the learner received information only about the boundary pair. Two presentations of the boundary pair were sufficient. The blue curves in both panels D and E assume perfect orthogonality between item representations **a** and context representations **c**, while the orange curves assume approximate orthogonality with an average cosine of 0.1. Mean and standard deviations in both panels D and E were computed from 100 training sessions with different random seeds across train-short and train-long conditions. The model learns rapidly even with just approximate orthogonality, reaching 100% accuracy in all sessions after just two updates. **F**, **G** 2D projections via MDS (Multidimensional scaling) of the response of the rank estimator neurons from panel A for the items from the two contexts after train short and after train long, respectively. **H**, **I** Representational dissimilarity matrices (RDMs) capture pairwise rank estimation distances as *RDM*_*i*,*j*_ = ∥**r**(**a**_*i*_) − **r**(**a**_*j*_)∥_2_ after short and long training, respectively. The same neural representation as for panels F and G was used.

Humans can rapidly reassemble knowledge about separately learned orders, for example merge two separately learned orders on the basis of new information. This process was studied in experiments with human subjects in ref. ^19^. They first learned two separate orders in phase 1, and in phase 2 that the first item in one order comes after the last item in the other order, i.e., the learned orders were appended to each other, see Fig. 5B, C.

The Rank Convergence Theorem guarantees that the Rank Learning Rule will also lead in this 2-phase learning process to rank estimates that are in the end consistent with all order relations for the combined order. We show that if area LIP receives besides synaptic input that encodes items also synaptic input that encodes the contexts A and B to which the items of the two short orders belong (see Fig. 4A and Fig. 5A), then fast reconfiguration of the internal model emerges (Fig. 5E). It is well-known that higher brain areas, especially the PFC, keep track of the current context, and signal it via spike inputs to virtually all other brain areas^35^. Furthermore, it is pointed out in that review that context is typically encoded in the PFC by firing patterns of neurons that are orthogonal to neural codes for other task-relevant variables^36^. This was recently confirmed for the human brain in ref. ^37^.

In order to model rapid configuration of internal order representations we complemented the Rank Learning Rule by a similarly simple Context Learning Rule for the weights of synaptic connections that project context information to area LIP (see Fig. 5A):

#### **Context learning rule**

Define an error signal *e*_*AB*_ that assumes value 1 if information is received that **a**_*i*_ ≺ **a**_*j*_ for some items **a**_*i*_ and **a**_*j*_ that come from two different contexts A and B and are encoded by neural codes **c**_*A*_ and **c**_*B*_, but (**wa**_*j*_ + **w**_*c*_**c**_*B*_) is not by some given margin *δ* larger than (**wa**_*i*_ + **w**_*c*_**c**_*A*_); else *e*_*AB*_ = 0. The Context Learning Rule is:

3 $${{{{\bf{w}}}}}_{c}\leftarrow {{{{\bf{w}}}}}_{c}+{\eta }_{c}\,{e}_{AB}\,({{{{\bf{c}}}}}_{B}-{{{{\bf{c}}}}}_{A}).$$

We used random vectors **c**_*A*_ and **c**_*B*_ to encode contexts and applied a larger learning rate *η*_*c*_ = 8 × *η* to the context weights **w**_*c*_ compared to the other weights **w**. More analysis of the effect of different learning rate ratios between *η*_*c*_ and *η* can be found in Supplementary Information Section D.2. Like *η*, the value of *η*_*c*_ is kept constant throughout the training process. Since the context information **c** is shared by all objects within a context, adjustments to **w**_*c*_ shift the rank estimates of all objects together. When the ratio between *η*_*c*_ and *η* is sufficiently high, learning on boundary pairs is prioritized by shifting each group as a whole, rather than adjusting only the boundary pair. The same mechanism naturally extends to more than two contexts. An example where three separately learned orders are merged is shown in Supplementary Information Section D.3.

The Context Learning Rule is basically the same as the Rank Learning Rule: It adds a fraction of the synaptic inputs for the item whose rank is too low to the current synaptic weights, and correspondingly applies LTD if the rank is too high. But it employs a separate learning rate for those components of the synaptic inputs that carry information about the context. A separate learning rate for these synaptic inputs is biologically plausible because context information from higher brain areas typically arrives in L1 of a cortical column, through synapses on distal apical dendrites of pyramidal cells, see the data of ref. ^38^. Thus context information arrives in a different dendritic compartment than bottom-up sensory inputs, that typically arrive closer to the soma. It is commonly assumed that different compartments of a biological neuron are subject to different plasticity processes (see, e.g., ref. ^39^). These are likely to have also different learning rates.

We tested the resulting learning model for synaptic connections to area LIP on the scenario from ref. ^19^. First, two different orders were learned in contexts A and B, see Fig. 5B, using the same weight vectors **w** and **w**_*c*_ for learning both orders. The learning curve is shown in 5D. For train long, we continued learning for these weight vectors with a boundary pair, consisting of the item that came last in the short order from context A and the first item in the other short order from context B. One sees in Fig. 5E that in general 2 presentations of the boundary pair suffice for adjusting all rank estimates for the long order.

MDS projections of the representations of the items after train short and after train long are shown in Fig. 5F, G. Hence this simple model leads to very similar results as the model of ref. ^19^ for the same experiment, although the architecture and learning processes were very different in the two models^19^. employed a multi-layer neural network that was trained through backpropagation (BP). Their model required in addition that on the side also a certainty matrix was learne for all pairs of items, that was not part of the neural network representation. It was needed to set differential learning rates for all pairs of items. Neither BP nor certainty estimates are needed in our model. In addition, the reconfiguration of ranks for the long order was substantially faster: two presentations of the boundary pair were sufficient, see Fig. 5E.

### Explaining curved 2D projections of neural representations of linear orders in the human brain

One structural difference between the neural network representation of a learned order in the brain and in the multilayer perceptron (MLP) model of ref. ^19^ had been addressed in the “Discussion” section of that study: 2D projections of fMRI data from the human brain have a characteristic horseshoe shape, i.e., they are curved (inflected). In contrast, 2D projections of the MLP representations of items in a learned total order from ref. ^19^ tend to form a line. Fig. 5F, G show that this also occurs in our model. However, we have considered so far only 2D projections of neural responses that encode ranks by a summation code. The brain encodes ranks also by neurons that fire most strongly for particular ranks or regions of ranks, referred to as heterogeneous code in ref. ^1^. We show that adding such a heterogenous encoding of ranks to our model reproduces the experimentally found horseshoe shape of 2D projections of neural representations of items of an order. In addition, we present a theoretical analysis which shows that this horseshoe shape, or in mathematical terms an approximation of a parabola, necessarily emerges in 2D projections of neural codes of the neurons have some overlap in their preferred ranks.

**Fig. 6:**
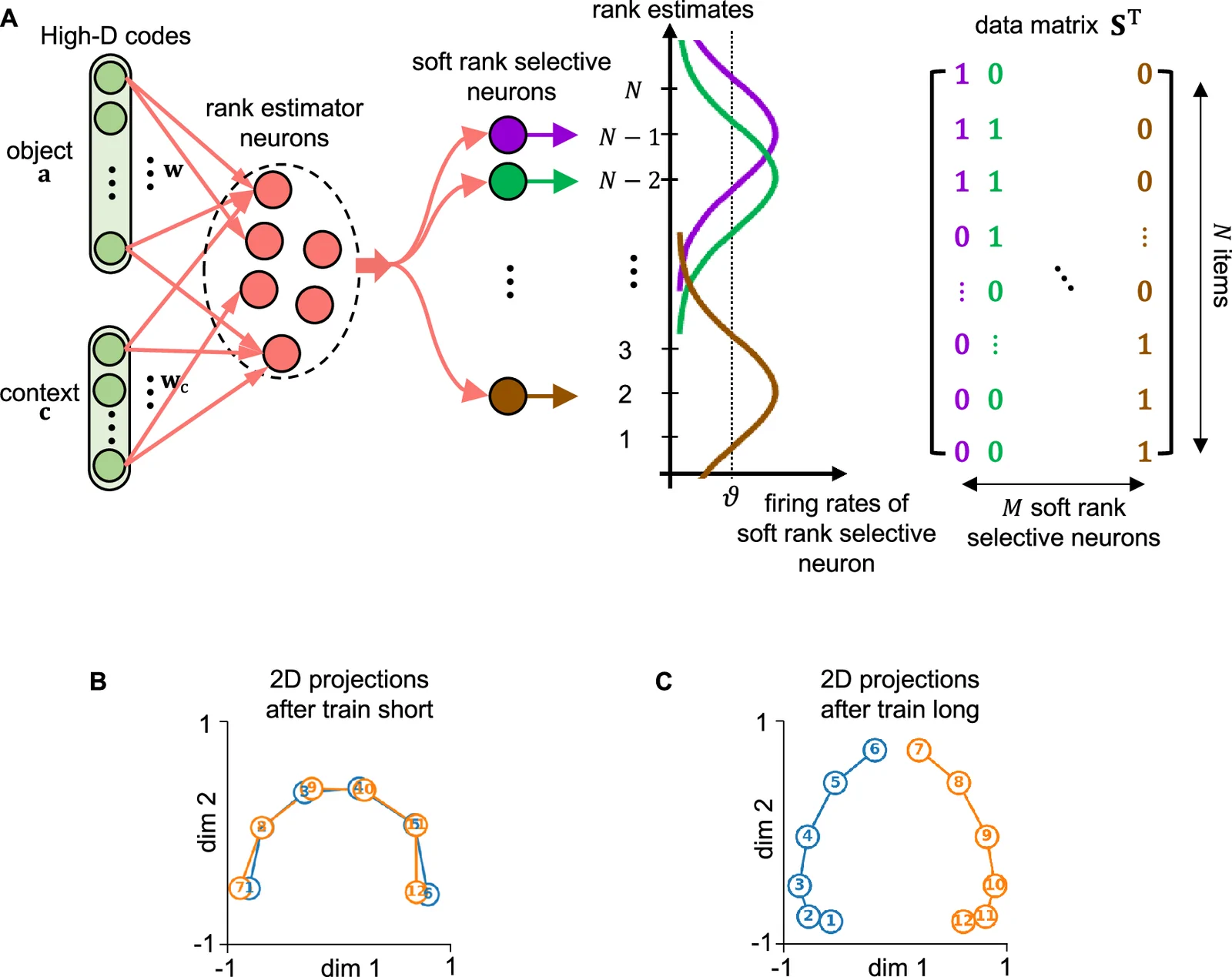
Emergence of a parabola (horseshoe) shape of 2D PCA (principal component analysis) projections through soft rank selective neurons. **A** Architecture for the generation of heterogeneous codes from a summation codes for rank estimates. Each colored circle in the middle indicates a population of excitatory neurons with negative feedback from inhibitory neurons, see Fig. S9 for a more detailed plot. This negative feedback curtails the firing rate in each population. On the other hand, staggered firing thresholds of excitatory neurons in different populations cause different onsets of their preferred ranks. Hence, the response profile of each population can be approximated by a Gaussian. If one binarizes their firing responses via some threshold, one arrives at the simple binary matrix on the right, which we analyze in section “Theoretical analysis of the shape of 2D projections of the firing activity of soft rank selective neurons for items in an order, discussed in section ‘Explaining curved 2D projections of neural representations of linear orders in the human brain’” of “Methods” and Section F of the Supplementary Information. **B** Shown are projections onto the first two PCA dimensions, i.e., onto the first two eigenvectors of the covariance matrix, of the patterns of firing activity of soft rank selective neurons for 6 items in the two separately learnt short orders. This is shown here after *training short*, but before *training long*. **C** 2D PCA of the same neural data after *training long*, revealing a characteristic horseshoe.

In order to model the emergence of a heterogeneous code for ranks it suffices to route the output of neurons that encode ranks in a summation code to a population of excitatory neurons with staggered firing thresholds, see Fig. 6A. It is well known that different biological neurons tend to have different excitability, see, e.g., refs. ^40–42^. The excitability of a biological neuron corresponds to the firing threshold of a simple neuron model. But also other factors, such as synaptic gain and inhibitory feedback regulate the firing response of a biological neuron. Each of these heterogeneous neurons will fire then only for ranks above a certain value. If these neurons are interconnected with inhibitory neurons so that several negative feedback loops arise (each enclosed by dashed lines in Fig. S9), very high ranks will activate this negative feedback so strongly that the neurons no longer respond to very high ranks. As a result, each excitatory neuron in the array becomes tuned to some interval of ranks. The width of this tuning is regulated by the strength of inhibition: According to ref. ^9^, a blockage of GABAergic inhibition leads to a broadening of the tuning profiles of these excitatory neurons. Hence, a network architecture as indicated in Fig. 6A, see Fig. S9 for a more detailed plot, leads to a diversity of tuning profiles of soft rank selective neurons with different approximately Gaussian tuning profiles, as indicated in the middle of Fig. 6A. 2D projections of the responses of these neurons yield horseshoe shaped responses, see Fig. 6B, C. These shapes reproduce qualitatively the responses from the human brain for the same experimental conditions (train short and train long) that were shown by ref. ^19^ Fig. 3C and Fig. 5B In Supplementary Information Section E.2, Figs. S10–S12, we demonstrate that this effect is preserved when the number of items in the order or the number of soft rank selective neurons grow, and that it is robust to noise imposed on their outputs.

A rigorous mathematical proof shows that the horseshoe shape has to emerge whenever a scalar variable is encoded by neurons with overlapping selectivity for values of a scalar varaible. The starting point of our analysis is the matrix on the right side of Fig. 6A. Each column of it records for one of the soft rank selective neurons for which ranks it is selective, in terms of its firing rate being above some threshold. Essential for our theoretical analysis is that adjacent columns have common 1s in some rows and these are shifted downwards from left to right.

Our theoretical explanation is based on an analysis of the covariance structure of the soft rank selective neurons: Their covariance matrix can be approximated by a circulant matrix, i.e., by a matrix where successive rows are shifted by one column, see Methods or ref. ^43^ for a precise definition. Furthermore, if the rank-selectivities of different neurons overlap, this circulant matrix does not degenerate to a diagonal matrix, and its first two eigenvectors have a cosine structure, see section “Theoretical analysis of the shape of 2D projections of the firing activity of soft rank selective neurons for items in an order, discussed in section ‘Explaining curved 2D projections of neural representations of linear orders in the human brain’” of “Methods.”

**Fig. 7:**
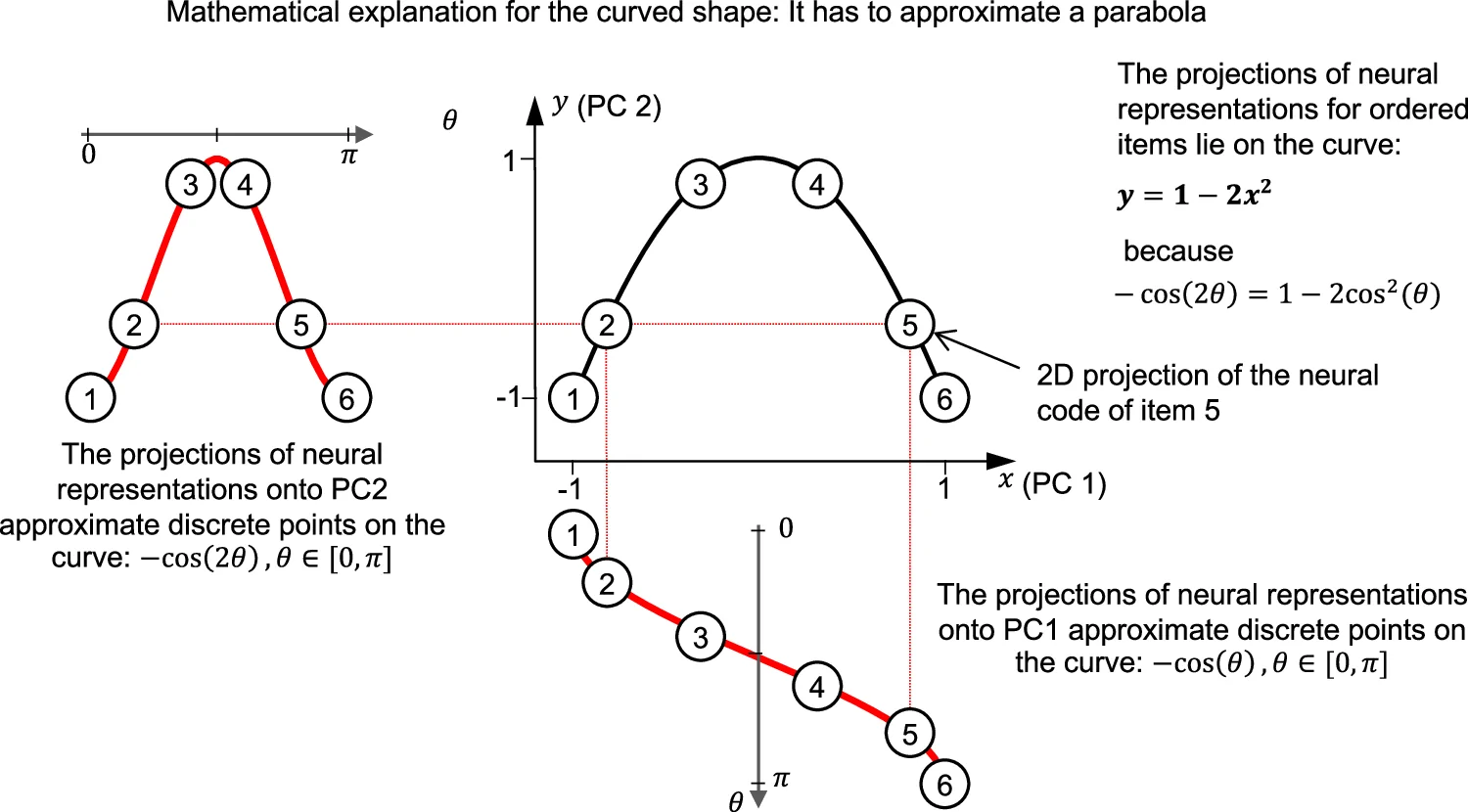
Mathematical illustration of the cause of the curved shape. According to Eq. (34) and (Eq. (35)), the projections of the firing activity of soft rank selective neurons onto the first principal component (PC1) approximate the cosine function over [0, *π*], see the lower part of the plot, while the projections on PC2 approximate the cosine over [0, 2*π*], see the plot at the left side. Combined, they come to lie on a parabola. This parabola explains why the horseshoe shape emerges in the PCA results of fMRI data. Note that we flipped the sign of both eigenvectors so that the PCA result resemble those in ref. ^19^.

Of particular interest for the analysis of the first two dimensions of a PCA projection are the projections of neural codes onto the first two eigenvectors, for which these approximations via the cosine function are shown in Fig. 7. More precisely, when ranks (1, …, *N*) are mapped to uniformly sampled phases *θ* ∈ [0, *π*], the projection of the activities of the soft rank–selective neurons onto the first principal component can be approximated by a cosine function over 180°, namely $\cos (\theta )$. Projections onto the second principal component can be approximated by a cosine function over 360°, namely $\cos (2\theta )$. Using the double-angle formula, $\cos (2\theta )=2{\cos }^{2}(\theta )-1$, one can approximate the shape of the 2D PCA projection by a parabolic function. This yields the horseshoe-shaped inflection in Fig. 6B, D. In section “Theoretical analysis of the shape of 2D projections of the firing activity of soft rank selective neurons for items in an order, discussed in section ‘Explaining curved 2D projections of neural representations of linear orders in the human brain’” of “Methods” and Section F of the Supplementary Information, we provide a full proof of this theoretical prediction. Note that the signs of eigenvectors can be chosen arbitrarily. Hence, PCA projections of the same data that are flipped upside down or left to right are equivalent from this perspective.

A horseshoe shape of 2D projections was also found for multi-unit electrode recordings from area LIP of the macaque for linearly ordered items, see Fig. 2E, F of ref. ^27^. Their emergence can also be explained by our theory. These panels also show that the approximation of the covariance matrix by a circulant matrix is already good enough for this small instance (with *N* = 6 items and *M* = 8 soft rank selective neurons) so that its eigenvectors of the covariance matrix behave qualitatively like those of a circulant matrix.

## Discussion

We have shown that the all-important latent variable for learning an order, the rank of an item in this order, can be learned by a simple plasticity rule for synaptic connections from neurons that encode items or context to neurons in area LIP that participate in the commonly observed representation of learned ranks through a summation code^1,6^, i.e., by a space rate code (see Fig. 1A). This latent variable has to be extracted from dispersed information about order relations between different pairs of items, and it is not a-priori clear whether this is possible. But the convergence of this extraction process is guaranteed by a new theoretical foundation, the Rank Convergence Theorem. The same online learning method may also explain the emergence of rank estimates in other brain areas, such as the PFC^18^.

The emergent S-shape of resulting rank estimates in our model (Fig. 2E) provides a principled explanation for the commonly observed “terminal effect” or “serial position effect” in human behavior, whereby transitive inference performance is enhanced when the comparison involves items located at the end of a linear order, see refs. ^16–18^. Using rank estimates for transitive inference also explains the commonly observed “symbolic distance effect”, whereby transitive inference performance increases linearly with the distance of items in the learned order. This effect arises automatically when transitive inference is based on rank estimates that are encoded in an internal model by firing rates: If the firing rates that encode the rank estimates for two items have a larger distance, this difference can be detected faster and more reliably by downstream networks.

A different model for learning linear orders had previously been proposed by ref. ^21^. Instead of a single neuron as in our model, they employed a completely connected RNN with 200 neurons in order to learn a linear order. The learning of the 40k synaptic weights of recurrent connections in this network was supported by a preceding meta-learning phase where 40k meta-parameters were optimized through an application of a sophisticated reinforcement learning algorithm, called Advantage-Actor-Critic, over 30k training episodes for these meta-parameters. Furthermore, an artificial neuromodulatory system was employed that was simultaneously optimized through this meta-learning process. In contrast, our model neither requires recurrent synaptic connections, nor meta-learning, nor an artificial neuromodulatory system. Furthermore. Our model comes with a convergence guarantee for learning linear orders, whereas their approach does not. Also, their model requires training phases where only pairs of items are presented that are adjacent in the learnt order. Hence they require global insight into the structure of the order before learning can begin. Further details of comparison can be found in section “Theoretical analysis of the shape of 2D projections of the firing activity of soft rank selective neurons for items in an order, discussed in section ‘Explaining curved 2D projections of neural representations of linear orders in the human brain’.”

The work^20^ had previously studied general properties of neural networks that are trained to solve the decision problem “does X come after Y in the order”, especially with regard to their resulting transitive inference capability. They focus on neural networks with non-additive representations, whereas our model uses an additive representation according to their terminology. In fact, our model seems to provide the simplest possible model for solving this decision task (by comparing the learned ranks of X and Y), since it consists of just a single linear neuron. Interestingly, they had shown that any model with an additive representation necessarily implements a ranking system.

If one takes into account that LIP neurons also receive information about the context of the current relational learning process, one can explain by our model also the emergence of a cognitive map that enables navigation in a 2D space that is spanned by ranks in two unrelated order relations, as shown for the human brain in ref. ^24^. Our model can also explain rapid reconfiguration of rank estimates in the light of new order relations, as addressed in the experiments and model of ref. ^19^. An interesting difference between the learning process in their multi-layer perceptron model and our simple model is that our model does not require Backprop training, and also no differential regulation of learning rates for different pairs of items is required for reconfiguration of learned order representations. As a result, substantially fewer learning steps are needed by our model for the reconfiguration of learned order relations in the face of new information.

The primary empirically testable prediction of our model is that learnt ranks of an order are represented by firing rates of neurons, integrated into the dominant 1D dynamics of area LIP (which had been elucidated by ref. ^2^). In other words, our model predicts that these ranks are represented in the brain in a similar fashion as numbers and other types of magnitudes, whose neural representation had been elucidated by refs. ^1, 6–14^. The data of ref. ^44^ suggest that this prediction has a good chance to be verified. They used an order learning task that was very similar to the one we used, but with the difference that two items of the order were always presented simultaneously. They found that for this type of presentation the sum of ranks of the two simultaneously presented items was represented by the firing rate of LIP neurons. Our model predicts that also for a presentation of a single item its learnt rank is likely to be represented by the firing rate of a neuron.

The experimental data of ref. ^24^ suggest that explicit representations of this latent scalar variable also emerge in the human brain, since the formation of a 2D cognitive map for items that are characterized by values of 2 scalar variables was so far only observed when explicit representations of the underlying two scalar variables were formed in the brain. But further experiments are needed to verify this for the case of rank representations in the human brain. Our model also predicts that outputs of LIP neurons that encode learnt rank estimates are routed to the entorhinal cortex, and play there a similar role for advancing grid cell firing as efferent copies of motor commands in the case of locomotion.

The models of refs. ^19–21^ did not predict that explicit representations of learnt ranks of items are formed in the brain, since these models consisted of neural networks with hidden units that were trained to solve a decision problem that does not require explicit representations of learnt ranks.

In addition, these preceding models predicted that complex top-down learning signals can be found in the brain, since these as needed for an implementation of BPTT in a multi-layer neural network. In contrast, our model predicts that only the ground truth about the order between two presented items, which is explicitly given to the subject, is transmitted as top-down learning signal, and a local synaptic plasticity process suffices.

The model of ref. ^15^ also predicted that explicit representations for ranks are formed in the brain. But this model is only applicable to the case where the subject receives for learning exclusively adjacent items from the order. But a selected presentation of only adjacent items requires a-priori information about the global structure of the order, which is in general not available to the subject. Empirical data suggest that orders can also be learnt without this constraint.

In the last part of the paper, we have proposed a theoretically founded solution to an open problem that was addressed in ref. ^19^: 2D projections of internal representations of ranks in the parietal cortex of the human brain are not straight, as predicted by most models, but on a horseshoe shaped curve. We have shown that if one takes into account that ranks are not only represented by summation codes of neurons, but also by soft rank selective neurons that preferentially fire for a certain range of ranks, the experimentally observed inflection of 2D projections automatically emerges. It results from the structure of the first two eigenvectors of circulant matrices that arise in the mathematical analysis of neurons with soft selectivity for ranks. In fact, this analysis is applicable also to neurons with soft selectivity for some other magnitude, thereby also providing a new and principled explanation for the experimentally observed curved manifolds for decision variables in the macaque brain^27^. Our theory predicts that horseshoe shaped curvature will arise in all 2D projections of brain activity that represents value of some scalar variable, provided that there exist neurons with soft selectivity for values of this variable.

The Rank Learning Rule that emerged from our normative approach is consistent with experimental data on synaptic plasticity in the brain, as discussed at the end of section “Order relations can be learnt by single neurons.”

Altogether, we have provided a new theoretical foundation for understanding how order relations are learnt and represented in the brain, and how these representations can give rise to intelligent decision making. These processes appear to be instrumental for enabling our brains to cope with a web of relations that impinge on us both from the environment and in the form of learn relations between abstract concepts. Our theory suggests that the computational and learning processes which are required for that are facilitated by inherent capabilities of neurons and synaptic plasticity that we have elucidated in this work. The stability and convergence of these learning processes is guaranteed by mathematical facts.

Apart from understanding brain processes, our theoretical foundation also provides algorithmic and architectural guidance for building energy-efficient edge devices that are able to cope with a web of relationships that impinge on them. It shows that fast learning and representations of multiple types of relations is possible that are easy to combine, for example in cognitive maps, in a way that supports low-latency decision making. Furthermore, in this work we have implemented the Rank Learning Rule with on-chip learning on a Loihi2 neuromorphic system^26^. Its strictly local form also suggests that the proposed rule is within reach of on-chip learning through memristor arrays, such as for example the ones described in ref. ^25^.

## Methods

### Details to the rank learning rule in section “Order relations can be learnt by single neurons”

Here, we introduce a loss function whose gradient descent optimization exactly reproduces the Rank Learning Rule.

Consider a fixed pair of items represented by coding vectors **a**_*i*_ and **a**_*j*_, where **a**_*i*_ ≺ **a**_*j*_, with a margin *δ* > 0 defining the minimal desired separation between their rank estimates. Let the firing rates (rank estimates) of the rank estimator neuron for these items be defined as *r*(**a**_*i*_) = **wa**_*i*_ and *r*(**a**_*j*_) = **wa**_*j*_.

We define a pairwise ranking loss function as follows:

4 $${L}_{i,j}=\left\{\begin{array}{ll}\delta -(r({{{{\bf{a}}}}}_{j})-r({{{{\bf{a}}}}}_{i})),\quad & \, {{\rm{if}}}\,(r({{{{\bf{a}}}}}_{j})-r({{{{\bf{a}}}}}_{i})) < \delta,\\ 0,\hfill &\,{{\rm{otherwise}}}. \hfill \end{array}\right.$$

This loss can be interpreted as a hinge-type ranking loss associated with the Kendall tau formulation^45–47^, where the standard unit margin is replaced by a general margin parameter *δ*. We provided a sensitivity analysis of the margin *δ* in Supplementary Information Section A.2. The loss for each item pair is convex, and therefore the average loss over all ordered pairs in a ranking remains convex with respect to the model parameters.

Minimizing this loss function (See eq. (39) in Supplementary Section A.1. for detail) via gradient descent leads precisely to the weight update given by the Rank Learning Rule:

5 $$\Delta {{{\bf{w}}}}=-\eta \frac{\partial {L}_{i,j}}{\partial {{{\bf{w}}}}}=\eta \cdot \underbrace{{e}_{ij}}_{\,{{\rm{gating}}}\,}\cdot \underbrace{({{{{\bf{a}}}}}_{j}-{{{{\bf{a}}}}}_{i})}_{\,{{\rm{pre}}}-{{\rm{synaptic}}}\,},{{{\rm{where}}}}\,{e}_{ij}=\left\{\begin{array}{ll}1,\quad &\,{{\rm{if}}}\,(r({{{{\bf{a}}}}}_{j})-r({{{{\bf{a}}}}}_{i})) < \delta,\\ 0,\quad &\,{{\rm{otherwise}}}\,.\hfill\end{array}\right.$$

A proof of convergence for the resulting weight update rule is provided in the next section.

### Proof of the rank convergence theorem and details of experiments, discussed in section “Learning theory for the Rank Learning Rule”

**Rank Convergence Theorem:**

Let **a**_1_, …, **a**_*K*_ be some arbitrary finite set of non-zero orthogonal vectors, to which we refer as items. We consider updates of weights **w** under the Rank Learning Rule:

6 $${{{{\bf{w}}}}}_{t+1}={{{{\bf{w}}}}}_{t}+\eta ({{{{\bf{a}}}}}_{j}-{{{{\bf{a}}}}}_{i}),$$

for arbitrarily selected pairs of items **a**_*j*_ and **a**_*i*_ with the property that **a**_*i*_ ≺ **a**_*j*_ but **w**_*t*_**a**_*j*_ is not by some margin *δ* larger than **w**_*t*_**a**_*i*_. The factor *η* is assumed to be some arbitrary positive fixed value.

Then **w**_*t*_ converges after finitely many steps to some value, independently of the order and frequency by which pairs of items **a**_*j*_ and **a**_*i*_ are selected for updates.

There are two different ways of proving the Rank Convergence Theorem: by a direct proof or by a reduction to the Perceptron Convergence Theorem.

**Direct proof:**

Assume, for contradiction, that the claim is false, i.e., that the learning process does *not* terminate, and infinitely many updates are performed by the Rank Learning Rule.

We consider the subset of items

7 $$T=\left\{{{{{\bf{a}}}}}_{k}| \begin{array}{ll}&\,{{\rm{there}}}\,\,\,{{\rm{are}}}\,\,\,{{\rm{infinitely}}}\,\,\,{{\rm{many}}}\,\,\,{{\rm{weight}}}\,\,\,{{\rm{updates}}}\,\,\,{{\rm{for}}}\,\,\,{{\rm{pairs}}}\,\\ &\,{{\rm{of}}}\,\,\,{{\rm{items}}}\,\,\,{{\rm{where}}}\,\,\,{{\rm{one}}}\,\,\,{{\rm{of}}}\,\,\,{{\rm{them}}}\,\,\,{{\rm{is}}}\,{{{{\bf{a}}}}}_{k}\hfill\end{array}\right\}.$$

By assumption, *T* is nonempty, and contains at least a pair of items in order to make infinitely many updates. We pick an element ${{{{\bf{a}}}}}_{\min }\in T$ that is minimal, among all items in set *T*, under the ordering  ≺ .

**Observation 1: **$${{{{\bf{w}}}}}_{t}{{{{\bf{a}}}}}_{\min }\to -\infty$$.

Since only finitely many updates are made for items ${{{{\bf{a}}}}}_{k}\prec {{{{\bf{a}}}}}_{\min }$ (by choice of ${{{{\bf{a}}}}}_{\min }$; and by definition of ${{{{\bf{a}}}}}_{\min }$, we know that **a**_*k*_ ∉ *T*), from some step *t* on, all weight updates that involve ${{{{\bf{a}}}}}_{\min }$ also involve some ${{{{\bf{a}}}}}_{\min }\prec {{{{\bf{a}}}}}_{k}$. Hence from some step *t* on only negative updates are made for ${{{{\bf{a}}}}}_{\min }$, i.e., $\eta {{{{\bf{a}}}}}_{\min }$ is subtracted from **w** whenever a weight update is made that involves ${{{{\bf{a}}}}}_{\min }$. Hence, the orthogonality of the items **a**_*i*_ implies that ${{{{\bf{w}}}}}_{t}{{{{\bf{a}}}}}_{\min }\to -\infty$.

**Observation 2:** A dual version of this argument shows that

8 $${{{{\bf{w}}}}}_{t}{{{{\bf{a}}}}}_{\max }\to+\!\infty,$$

for the maximal item ${{{{\bf{a}}}}}_{\max }$ in *T* with regard to  ≺.

**Observation 3:** Let item **a**_*k*_ in *T* have the property that **w**_*t*_**a**_*k*_ → −*∞* and **w**_*t*_**a**_*l*_ → −*∞* for all **a**_*l*_ in *T* with **a**_*l*_ ≺ **a**_*k*_. If **a**_*q*_ is the next larger item in *T* after **a**_*k*_ with regard to  ≺, then also **w**_*t*_**a**_*q*_ → −*∞*.

This holds because after a while, all updates of **w** where *η***a**_*q*_ is added to **w**_*t*_ are made at steps *t* when **w**_*t*_**a**_*q*_ ≤ **w**_*t*_**a**_*r*_ + *δ* for some **a**_*r*_ in *T* with **a**_*r*_ ≺ **a**_*q*_. Since by assumption **w**_*t*_**a**_*r*_ → −*∞* for all these **a**_*r*_, the products **w**_*t*_**a**_*q*_ that occur at these steps also converge to −*∞*.

Induction along  ≺, where the induction beginning is provided by Observation 1 and the induction step by Observation 3, implies then that **w**_*t*_**a**_*k*_ → −*∞* for all **a**_*k*_ in *T*. But this yields a contradiction to Observation 2.

This completes the direct proof of the Rank Convergence Theorem.

For the special case where the margin *δ* has value 0 one can reduce this proof to a proof of the Perceptron Convergence Theorem in the following way.

**Reduction of the special case to the proof of the perceptron convergence theorem (which is discussed, for example, in refs.**^48,49^**)**:

A perceptron is a linear gate with a threshold, which gives binary outputs. Hence, it cannot be trained directly to output rank estimates for items in a linear order. But it turns out that if one trains a perceptron on the problem to decide for input *a*_*j*_ −*a*_*i*_ whether *a*_*j*_ comes after *a*_*i*_ in the linear order, and the perceptron has learned to carry out this classification correctly for all pairs of items, its weight vector **w** has the desired property of the weight vector for the Rank Convergence Theorem, i.e., the inner products **wa**_**i**_ provide a correct ordered estimates of the ranks of the items *a*_*i*_.

One first has to realize that the weight updates of a perceptron for this classification task (applied differences *a*_*j*_ −*a*_*i*_) are the same as the weight updates Eq. (1) of the Rank Learning Rule. In the next step one has to verify that the assumption of the Perceptron Convergence Theorem, that there exists a hyperplane that can carry out the desired classification task, is met in this application of the perceptron to differences *a*_*j*_ −*a*_*i*_. This holds because the weight vector **w** constructed in Eq. (2) defines such a hyperplane.

In order to make sure that an upper bound for the number of weight updates of the perceptron learning rule also provides an upper bound for number of weight updates of the Rank Learning Rule one has to verify a third item: That each time when a weight update occurs in the Rank Learning Rule, also an error occurs in the perceptron learning rule for the corresponding learning problem. But a closer look shows that this only holds for the special case where the margin *δ*ğ in the Rank Learning Rule has value 0. In order to reduce the proof of the general version of the Rank Learning Theorem (with arbitrary margins *δ*) to a result for perceptron learning, one has to resort to an extension of the proof of the Perceptron Convergence Theorem that had been given in ref. ^50^.

On the side we would like to point to the interesting fact that the direct proof of the Rank Convergence Theorem is quite different from the standard proof of the Perceptron Convergence Theorem.

#### Details to learning of a given order in Fig. 2

In Fig. 2D, we trained our model on adjacent pairs among the 10 items, and tested on unseen non-adjacent pairs among the same set of items. The learning rate *η* in Eq. (5) is set to 0.1. Each item code **a**_*i*_ is a 10^4^ dimensional random binary vector with number of ones uniformly sampled within the range [0.2%, 1%]. The blue curve is measured when all **a**_*i*_ forms an orthogonal set; and the orange curve is measured when the average cosine similarity between all pairs of items’ code is 0.1. Each synaptic weight in **w** is initialized by a normal distribution ${{{\mathcal{N}}}}(0,1{0}^{-4})$. The weights are initialized very small to prevent unnecessary random biases that decreases the learning speed. The standard deviations of the two curves are calculated by repeated training from scratch 100 times. The randomness comes from data sampling, as a random pair of adjacent objects is picked at each training step. The margin *δ* is set to 1.

#### Method for generating codes for items with a given average pairwise cosine similarity

After initializing each item code with a fixed number of ones, this number is kept constant for each item. The indices are adjusted either to overlap with the dimensions of other items’ ones, increasing the average cosine similarity, or to shift overlapping ones to different dimensions, reducing the cosine similarity. For a given target average cosine similarity, this process is iterated for a sufficiently long time to achieve a numerically optimal coding configuration.

### Details to our neuromorphic implementation on Loihi2, discussed in section “Neuromorphic implementation”

We implemented the Rank Learning Rule on a Loihi2 neuromorphic system. The task consists of learning a linear order over 10 items. Each item is represented by a one-hot vector, so that the inputs **a**_*i*_ and **a**_*j*_ are both 10-dimensional binary vectors with a single active component.

A single linear neuron learns to estimate ranks. Its synaptic weight vector ${{{\bf{w}}}}\in {{\mathbb{Z}}}^{10}$ maps each item code to a scalar rank estimate. It turns out that 6 bits suffice for each weight. Each weight takes values in [0, 63] and is initialized to 32. The rank estimate of an item is given by

9 $${r}_{i}={{{\bf{w}}}}{{{{\bf{a}}}}}_{i},\quad {r}_{j}={{{\bf{w}}}}{{{{\bf{a}}}}}_{j}.$$

The neuron is implemented using Loihi2 microcode, which allows programmable neuron dynamics beyond standard integrate-and-fire models. Each neuron has access to dendritic accumulators, internal state variables, and a custom instruction sequence that defines its behavior. Input spikes are first summed in dendritic accumulators before being processed by the neuron program.

The temporal computation follows a short sequence (Fig. 3C). Upon presentation of the first item **a**_*j*_, the neuron computes and stores its rank estimate *r*_*j*_ in internal state. When the second item **a**_*i*_ arrives, the neuron computes the rank difference

10 $$\Delta {r}_{ij}={{{\bf{w}}}}{{{{\bf{a}}}}}_{j}-{{{\bf{w}}}}{{{{\bf{a}}}}}_{i}.$$

This computation is implemented across a small number of microcode operations within a program pass. Each pass executes a sequence of arithmetic and logic instructions over a fixed set of registers, allowing subtraction, comparison, and state updates within a few hardware cycles. Multiple passes enable temporal logic, such as storing the first input and combining it with the second.

Due to the integer-valued representation and limited precision, we set *δ* = 1, so learning depends only on whether the predicted order matches the ground truth: Δ*r*_*ij*_ > 0 (i.e., at least 1 in the integer case) corresponds to **a**_*j*_ ≻ **a**_*i*_, and Δ*r*_*ij*_ < 0 to **a**_*j*_ ≺ **a**_*i*_.

The predicted rank difference Δ*r*_*ij*_ is combined with the ground truth order in a comparison module to produce an error signal *e*_*ij*_. For clarity, this module is implemented on the host CPU. The same functionality can be realized inside the neuromorphic core by introducing an additional neuron that receives both the predicted comparison and the ground truth as inputs and computes the error signal locally. We keep it outside to maintain a clear separation between the core learning mechanism and auxiliary comparison logic. The error signal is defined as

11 $${e}_{ij}=\left\{\begin{array}{ll}0,\quad &\,{{\rm{if}}}\,\,\,{\rm{the}}\,\,\,{{\rm{predicted}}}\,\,\,{\rm{order}}\,\,\,{{\rm{agrees}}}\,\,\,{{\rm{with}}}\,\,\,{{\rm{the}}}\,\,\,{{\rm{ground}}}\,\,\,{{\rm{truth}}}\,\\+1,\quad &\,{{\rm{if}}}\,{{{{\bf{a}}}}}_{j}\succ {{{{\bf{a}}}}}_{i}\,{{\rm{but}}}\,\Delta {r}_{ij}\le 0\hfill\\ -1,\quad &\,{{\rm{if}}}\,{{{{\bf{a}}}}}_{j}\prec {{{{\bf{a}}}}}_{i}\,{{\rm{but}}}\,\Delta {r}_{ij}\ge 0\hfill\end{array}\right..$$

When item pairs are always presented in a fixed order, the error signal reduces to a binary variable

12 $${e}_{ij}\in \{0,1\},$$

which only indicates whether the predicted order matches the ground truth.

The synaptic weights are updated locally according to

13 $$\Delta {{{\bf{w}}}}=\eta \,{e}_{ij}\,({{{{\bf{a}}}}}_{j}-{{{{\bf{a}}}}}_{i}),$$

where *η* is the learning rate. We set *η* = 1 in this experiment. Since both **a**_*i*_ and **a**_*j*_ are one-hot vectors, each update modifies only two synapses.

All computations related to rank estimation, temporary storage, and synaptic updates are implemented within a single neuromorphic core. The design relies on dendritic accumulation of inputs, programmable neuron logic, and local state variables for storing intermediate results. These features allow the full learning rule to run with only local information and event-driven updates.

### Details to our model for the emergence of cognitive maps from several learnt order relationships, discussed in section “Emergence of cognitive maps from several learnt order relationships”

#### Context selection

The architecture in Fig. 4 requires distinct subgroups of rank-estimator neurons to be selected for encoding item ranks under different contexts, a property reported by ref. ^33^. This can be implemented in multiple ways. Here, we show arguably the simplest method: Using fixed, high-dimensional sparse-binary context codes together with a randomly initialized high-D sparse-binary weight matrix **w**_*c*_, each context is automatically routed to a distinct, non-overlapping subgroup of LIP neurons without any synaptic learning. Other learning rules that introduce competitive mechanisms between LIP neurons-such as lateral inhibition or winner-take-all (WTA)-may also achieve the same effect.

Let each context *c* ∈ {1, …, *K*} be represented by a binary vector ${{{{\bf{c}}}}}^{(c)}\in {\{0,1\}}^{{N}_{c}}$, and assume approximate orthogonality:

14 $${{{{\bf{c}}}}}^{(i)}\cdot {{{{\bf{c}}}}}^{(j)}\ \approx \ 0\quad \,{{\rm{for}}}\,i\,\ne \,j.$$

For a randomly initialized sparse-binary weight matrix ${{{{\bf{w}}}}}_{c}\in {\{0,1\}}^{N\times {N}_{c}}$ (sparsity *p* ≪ 1, mapping input vectors of dimension *N*_*c*_ to *N* LIP neurons) whose *N*_*c*_ columns satisfy that any two distinct columns are also approximately orthogonal:

15 $${({{{{\bf{w}}}}}_{c})}_{*j}\cdot {({{{{\bf{w}}}}}_{c})}_{*k}\ \approx \ 0\quad \,{{\rm{for}}}\,j\,\ne \,k.$$

Upon presentation of context **c**, the net drive to the downstream population is

16 $${{{\bf{h}}}}={{{{\bf{w}}}}}_{c} \,{{{\bf{c}}}}.$$

One sees that **h** is exactly the sum of the columns of **w**_*c*_ selected by the non-zero index of **c**.

Because the context codes are orthogonal and the columns of **w**_*c*_ are orthogonal,

17 $${{{{\bf{h}}}}}^{(i)}\cdot {{{{\bf{h}}}}}^{(j)}={({{{{\bf{h}}}}}^{(i)})}^{\top}{{{{\bf{h}}}}}^{(j)}={({{{{\bf{c}}}}}^{(i)})}^{\top}({{{{\bf{w}}}}}_{c}^{\top }{{{{\bf{w}}}}}_{c}){{{{\bf{c}}}}}^{(j)} \approx {({{{{\bf{c}}}}}^{(i)})}^{\top}{{{{\bf{c}}}}}^{(j)} \approx 0\quad (i \,\ne \,j).$$

Therefore, each context selects approximately distinct subgroup of neurons. When these context inputs induce subthreshold depolarization in specific neuronal ensembles, subsequent object-driven signals can then selectively recruit different LIP subpopulations based on that context.

#### PCA of grid cells

The grid cells naturally form a representation of any 2D space. In Fig. 4A, we showed a 2D projection of grid activations on a (4 × 4) square environment via PCA.

The grid cells are randomly initialized. With 1000 grid cells, each initialized independently and modeled as the sum of three planar cosine gratings $r({{{\bf{x}}}})={\sum}_{j=1}^{3}\cos ({{{{\bf{k}}}}}_{j}\,\cdot \,{{{\bf{x}}}}+{\phi }_{j})$, whose wave-vectors **k**_*j*_ are 60° apart and share magnitude ∣**k**_*j*_∣ = 2*π*/*λ*. The grid scale was drawn once per cell from *λ* ~ *U*(0.05, 8). To eliminate directional bias a global rotation *θ*_0_ ~ *U*(0, 2*π*) was applied, giving $\arg ({{{{\bf{k}}}}}_{j})={\theta }_{0}+j\pi /3$.

The PCA is performed on the grid activations of 50 × 50 evenly sampled mesh-grid locations on the square environment.

### Details to combining separately learned orders into one order, discussed in section “Rapid reconfiguration of the internal model when separately learned orders are combined on the basis of new information”

#### Architecture

When generate the MDS plot in Fig. 5F, G, we use a population of rank estimator neurons **n**’s activations to encode rank information collectively. The population in this experiment contains 100 rank estimator neurons. These rank estimator neurons receive summation inputs from both codes, with each neuron’s value *r*:

18 $$r={{{\bf{w}}}}{{{\bf{a}}}}+{{{{\bf{w}}}}}_{c}{{{\bf{c}}}}.$$

Each neuron learns independently its incoming synaptic weights based on the same learning rule Eq. (5) as a single rank estimator neuron. The symmetry breaks because of the different initialization with each synaptic weights sampled from ${{{\mathcal{N}}}}(0,1{0}^{-4})$.

#### Train short

During initial training (”train short”), we divided the 12 objects into two distinct sets (context A 1-6 and context B 1-6) and presented in alternating trails. The model only made comparisons within each context and indicate which of two adjacent-ranked objects (e.g., context A 3 and context A 4) was larger or smaller, receiving fully informative feedback. In total, the model is trained on 80 pairs of examples to form the results in Fig. 5F, H. This training allowed our model to infer ranks within each set (i.e., context A 1–6 and context B 1–6) but did not reveal any information about the rank relations between the two sets (e.g., context A 2  < context B 3). We set the learning rate *η* = 0.1. Given that training occurs only between items from the same context, no weight updates to **w**_*c*_ would occur based on the Context Learning Rule.

#### Train long

In ref. ^19^, human participants underwent 20 training trials (each contains a pair of object together with their ranking information) after being informed that the lowest ranked object “6” in context A is ranked lower than the lowest ranked object “1” in context B. We replicated this setting, and reproduced their results by training our model on this boundary pair for 2 times, only 1/10 of the total training trials of the human participants. The result is shown in Fig. 5G and I.

The key of assembling two order is to shift the rank estimation of a whole group together, without altering the in-context ranking, which, in our model, corresponds to prioritize the learning to happen on the context information than the item code. By analyze the contribution by different variables on the rank estimation changes after applying the weight updating in Eq. (5), with an assumption that codes are orthogonal, one gets:

19 $${{\Delta}} r \propto \underbrace{\eta ||{{{\mathbf{a}}}}||_{2}^{2}}_{{{{\mathbf{a}}}}^{\prime }{{{\rm{s}}}}\,{{{\rm{contribution}}}}}+\underbrace{\eta_{c} ||{{{\mathbf{c}}}}||_{2}^{2}}_{{{{\mathbf{c}}}}^{\prime }{{\rm{s}}}\,{{\rm{contribution}}}}.$$

This result from our model suggests that two potential factors may contribute to the context-prior effect: 1. The learning rate for the context-related weights *η*_*c*_ is higher than the learning rate *η* for the object weights. 2. The *l*_2_-norm of the context code **c** is larger than that of the object code **a**. A detailed analysis of how these two factors influence the prioritization of learning based on context information is provided in the Supplementary Information Section D.1. In our experiment, we set *η*_*c*_ = 0.4 and *η* = 0.05; the dimension *D* = 10^4^ of **a** equals to the dimension of **c**; the sparsity of **a** equals to the sparsity of **c** with 0.2% to 1% (sampled from the uniform distribution) neurons fire in each code, which is based on the data from human medial temporal lobe^22,51^.

### Theoretical analysis of the shape of 2D projections of the firing activity of soft rank selective neurons for items in an order, discussed in section “Explaining curved 2D projections of neural representations of linear orders in the human brain”

We analyze the application of PCA for reducing the dimensionality of the representation of *N* items by *M* soft rank selective neurons from *M* to 2. The goal of this section is to derive a closed-form solution for the projection results.

The structure of this section follows the steps of computing PCA:

- Calculation of the covariance matrix
- Approximation and eigenvector calculation of the covariance matrix
- Calculation of the projections of neural representations onto the first two principal components

#### Calculation of the covariance matrix

Assume that we have *M* soft rank selective neurons *s*_1_ to *s*_*M*_ for items **a**_1_ to **a**_*N*_ in an order. We denote in the following the response of neuron *s*_*i*_ to item **a**_*j*_ as **s**_*i*_(*j*).

We now consider the case where

20 $${{{{\bf{s}}}}}_{i}[\,j]=\left\{\begin{array}{ll}1,\quad &\,{{\rm{if}}}\,j\in \{i,i+1,\ldots,i+{O}_{v}\}\\ 0,\quad &\,{\rm{otherwise}}\hfill\,.\end{array}\right.$$

These are the columns in the example matrix **S** as shown in Fig. 6A. In this equation, *O*_*v*_ (overlap) represents the number of items for which a soft rank-selective neuron fires beyond its central rank. This formulation approximates Gaussian tuning curves using rectangular windows, setting responses to zero for ranks distant from each Gaussian center, where the responses are expected to be negligibly small.

The responses of the *M* soft rank-selective neurons to the *N* items are collected in the matrix

21 $${{{\bf{S}}}}=\left[\begin{array}{c}{{{{\bf{s}}}}}_{1}\\ \vdots \\ {{{{\bf{s}}}}}_{N}\end{array}\right]\in {{\mathbb{R}}}^{N\times M},$$

where ${{{{\bf{s}}}}}_{i}\in {{\mathbb{R}}}^{M}$ denotes the population response to item *i*. After mean-centering across items, i.e.,

22 $${{{{\bf{S}}}}}_{c}={{{\bf{S}}}}-{{{\bf{1}}}}{{{{\boldsymbol{\mu }}}}}^{\top },\qquad {{{\boldsymbol{\mu }}}}=\frac{1}{N}\mathop{\sum}_{i=1}^{N}{{{{\bf{s}}}}}_{i},$$

with ${{{\bf{1}}}}\in {{\mathbb{R}}}^{N}$ the all-ones vector, the covariance matrix of neuronal responses is given by

23 $${{{\bf{C}}}}=\frac{1}{N-1}\,{{{{\bf{S}}}}}_{c}^{\top }{{{{\bf{S}}}}}_{c}\in {{\mathbb{R}}}^{M\times M}.$$

The principal components correspond to the eigenvectors of **C** associated with its largest eigenvalues. Without additional structure, these eigenvectors do not admit a closed-form expression, motivating the simplifying assumptions introduced below.

#### Approximation and eigenvector calculation of the covariance matrix

We show that **C** is a banded symmetric Toeplitz matrix, and hence can be approximated by a circulant matrix. Then we use properties of the eigenvectors of circulant matrices.

**Assumption**. Assume *N* is reasonably large so the edge effect can be neglected. Specifically, we assume that:

24 $$\forall j\in [1,M],{{{\rm{we}}}}\,{{{\rm{have}}}}\,{{{\boldsymbol{\mu }}}}[j]\approx \frac{{O}_{v}+1}{N}.$$

This equation holds rigorously for items that are not at an end of an order.

The centered data vectors then can be approximated by:

25 $${s}_{i}^{c}[j]\approx \left\{\begin{array}{ll}1-\frac{{O}_{v}+1}{N},\quad &\,{{\rm{if}}}\,i\le j\le i+{O}_{v}\\ \,\quad &\\ -\frac{{O}_{v}+1}{N},\quad &\,{\rm{otherwise}}\,. \hfill \end{array}\right.$$

Since $\frac{{O}_{v}+1}{N}$ is small for large *N*, we can further approximate:

26 $${s}_{i}^{c}[j]\approx \left\{\begin{array}{ll}1,\quad &\,{{\rm{if}}}\,i\le j\le i+{O}_{v}\\ 0,\quad &\,{\rm{otherwise}}\,. \hfill \end{array}\right.$$

Under our assumption, the covariance matrix **C** is symmetric and Toeplitz, meaning that **C**_*i*,*j*_ depends only on ∣*i* −*j*∣. This assumption captures the dominant bulk structure induced by overlapping tuning, while deviations from exact Toeplitz form arise primarily from boundary effects and do not qualitatively alter the low-dimensional geometry.

The covariance between dimensions *i* and *j* is:

27 $${{{{\bf{C}}}}}_{i,j}=\frac{1}{N-1} \sum_{k=1}^{N}{s}_{k}^{c}[i]{s}_{k}^{c}[j].$$

Given the approximated centered data, ${s}_{k}^{c}[i]{s}_{k}^{c}[j]$ is non-zero only when neural representations of items overlap in dimensions *i* and *j*. Hence, we can approximate **C**_*i*,*j*_ by:

28 $${{{{\bf{C}}}}}_{i,j}=\left\{\begin{array}{ll}({O}_{v}+1)/(N-1),\quad &\,{{\rm{if}}}\,| i-j|=0\\ \,{O}_{v}/(N-1),\hfill &\,{{\rm{if}}}\,| i-j|=1\\ ({O}_{v}-1)/(N-1),\quad &\,{{\rm{if}}}\,| i-j|=2\\ \vdots \hfill \\ 0,\hfill\quad &\,\,\,\,\,{{\rm{if}}}\,| i-j| > {O}_{v}\end{array}\right..$$

The form of the resulting covariance matrix is the **banded symmetric Toeplitz matrix** that satisfies the following properties:

- **Toeplitz structure:** The matrix has constant values along each diagonal, i.e., **C**_*i*,*j*_ = **C**_*i*+*k*,*j*+*k*_ for all *i*, *j*, *k* such that the indices are within bounds.
- **Symmetry:** The matrix is symmetric, meaning **C**_*i*,*j*_ = **C**_*j*,*i*_.
- **Banded structure:** The matrix has nonzero entries only within a fixed number of diagonals. Specifically, there exists a bandwidth *b* ≥ 0 such that **C**_*i*,*j*_ = 0 whenever ∣*i* −*j*∣ > *b*. We have *b* = *O*_*v*_ in our setting as depict in Eq. (28).

An example **C** is provided below:

29 $${{{\bf{C}}}}=\left(\begin{array}{ccccc}{c}_{0}&{c}_{1}&\cdots \,&\cdots \,&{{{\mathbf{0}}}}\\ {c}_{1}&{c}_{0}&{c}_{1}&\cdots \,&\vdots \\ \vdots &{c}_{1}&{c}_{0}&\ddots &\vdots \\ \vdots &\ddots &\ddots &{c}_{0}&{c}_{1}\\ {{{\mathbf{0}}}}&\cdots \,&\cdots \,&{c}_{1}&{c}_{0}\end{array}\right).$$

To obtain analytical insight into the eigen-structure of the covariance matrix, we further approximate the banded symmetric Toeplitz matrix by a circulant matrix. This step removes boundary effects that break exact translational invariance in finite *M* Toeplitz matrices and enables a closed-form characterization of the eigenmodes. A banded symmetric Toeplitz covariance matrix can be approximated by a **circulant matrix **${{{\bf{C}}}}^{\prime} \in {{\mathbb{R}}}^{M\times M}$^43^, which, by definition, has a:

- **Cyclic row structure:** Each row of ${{{\bf{C}}}}^{\prime}$ is obtained by shifting the preceding row one element to the right (wrapping around at the boundaries). If the first row is
  $$(c^{{\prime} }_{0},c^{{\prime} }_{1},\ldots,c^{{\prime} }_{n-1}),$$
  then the *i*-th row is given by
  $$(c^{{\prime} }_{(-i){{\mathrm{mod}}}\,n},\,c^{{\prime} }_{(1-i){{\mathrm{mod}}}\,n},\,\ldots,\,c^{{\prime} }_{(n-1-i){{\mathrm{mod}}}\,n}).$$

If one approximates the example banded symmetric Toeplitz matrix **C** above by a circulant matrix ${{{\bf{C}}}}^{\prime}$, then ${{{\bf{C}}}}^{\prime}$ would have the following form:

30 $${{{{\bf{C}}}}}^{{\prime} }=\left(\begin{array}{cccccc}{c}_{0}&{c}_{1}&0&\cdots \,&0&{c}_{1}\\ {c}_{1}&{c}_{0}&{c}_{1}&0&\cdots \,&0\\ 0&{c}_{1}&{c}_{0}&\ddots &&\vdots \\ \vdots &0&\ddots &\ddots &\ddots &0\\ 0&\cdots \,&&\ddots &{c}_{0}&{c}_{1}\\ {c}_{1}&0&\cdots \,&0&{c}_{1}&{c}_{0}\end{array}\right).$$

The only difference compared to **C** is that ${{{\bf{C}}}}^{\prime}$ requires an additional elements *c*_1_ in the upper right and lower left corner to fulfill the circulant property. Using a circulant matrix to approximate a banded Toeplitz matrix becomes more accurate as the size of the matrix *M* grows and the bandwidth *O*_*v*_ becomes narrower. When this approximation is imperfect, the resulting eigenvectors deviate from exact cosine forms, with discrepancies localized near boundary indices.

Importantly, a circulant matrix has **eigenvectors that can be represented by the columns of the discrete Fourier transform (DFT) matrix**^43^. In the case of a real symmetric circulant matrix, the DFT eigenvectors can be re-expressed in a real-valued form. Specifically, the discrete cosine transform (DCT) provides an orthogonal basis that yields a closed-form representation of the eigenvectors^52^:

31 $${{{{\bf{u}}}}}_{k}[j]=\cos \left(\frac{\pi k(2j+1)}{2M}\right),$$

where *k* = 1, 2, …, *M* is the mode index, and *j* = 1, 2, …, *M* is the position index of each eigenvector. The proof of the validity of this step can be found in the Supplementary Information Section F. Notably, we require overlap between rank selectivities so that the circulant matrix does not degenerate into a purely diagonal form. In that case, the absence of off-diagonal structure implies that, although cosine functions remain valid eigenvectors, the eigenvectors are not constrained to be cosine functions.

We focus on the first two principal components, which correspond to the eigenvectors with the largest two eigenvalues (usually corresponding to *k* = 1 and *k* = 2):

- **First eigenvector:**
  $${{{{\bf{u}}}}}_{1}[j]=\cos \left(\frac{\pi (2j+1)}{2M}\right).$$
- **Second eigenvector:**
  $${{{{\bf{u}}}}}_{2}[j]=\cos \left(\frac{2\pi (2j+1)}{2M}\right).$$

#### Calculation of the projections of neural data onto the first two principal components

The projection of ${{{{\bf{s}}}}}_{i}^{c}$ onto the *k*-th principal component is:

32 $${({{{{\bf{q}}}}}_{i})}_{k}={{{{\bf{u}}}}}_{k}{{{{\bf{s}}}}}_{i}^{c}=\sum_{j=1}^{M}{{{{\bf{u}}}}}_{k}[\,j]{{{{\bf{s}}}}}_{i}^{c}[\,j].$$

Substituting the approximated **u**_*k*_[*j*] and ${{{{\bf{s}}}}}_{i}^{c}[j]$ from eq. (31) and eq. (26), we get:

33 $${({{{{\bf{q}}}}}_{i})}_{k}=\sum_{j=i}^{i+{O}_{v}}\cos \left(\frac{\pi k(2j+1)}{2M}\right).$$

An intuitive observation is that the projection result can be viewed as a windowed sum of cosine values from *i* to *i* + *O*_*v*_, where *O*_*v*_ (overlap) represents the number of items for which a soft rank-selective neuron fires beyond its central rank. With *k* = 1 and under the assumption that the window is short (i.e., small *O*_*v*_), this windowed sum approximates a slightly smoothed cosine function over [0, *π*] for *i* ∈ [0, *N*] (Fig. 7, *x*-axis). Therefore, one gets the projection onto PC1 as:

34 $$x(i)=\sum_{j=i}^{i+{O}_{v}}\cos \left(\frac{\pi (2j+1)}{2M}\right)\approx \cos (\theta ),\,\,{{{\rm{where}}}}\,\,\theta=\frac{\pi (2i+1)}{2M}.$$

Similarly, the projection onto PC2 (*k* = 2) follows a discrete cosine approximation over [0, 2*π*] for *i* ∈ [0, *N*] (Fig. 7, *y*-axis):

35 $$y(i)=\sum_{j=i}^{i+{O}_{v}}\cos \left(\frac{2\pi (2j+1)}{2M}\right)\approx \cos (2\theta ),\,\,{{{\rm{where}}}}\,\,\theta=\frac{\pi (2i+1)}{2M}{{{\rm{as}}}}\,{{{\rm{above}}}}.$$

With $(x,y)\approx (\cos (\theta ),\cos (2\theta ))$, where *θ* ∈ [0, *π*], and using the trigonometric identity $\cos (2\theta )=2{\cos }^{2}(\theta )-1$, one can derive the explicit shape of the PCA results as:

36 $$y=2{x}^{2}-1,$$

which follows a parabolic curve.

In addition, the property of eigenvectors allows the sign of each eigenvector to be chosen freely. To better align with the PCA results in ref. ^19^, we flipped the sign of the projection result on both axes: $(x,y)\approx (-\cos (\theta ),-\cos (2\theta ))$ and get:

37 $$y=1-2{x}^{2},$$

which explains the inflection effect in Fig. 7.

### Comparison with the model of ref. ^21^ for learning linear relations

We are adding here further details to the comparison of our model for learning linear orders with that of ref. ^21^. Our model is consistent with the largely linear dynamics of area LIP according to ref. ^2^, whereas linear neural dynamics does not play a prominent role in their model. Furthermore, even without any meta-learning, our model shows a similar learning speed as their model: It reaches 90 percent accuracy after 19 presentations of adjacent pairs, compared with 20 pair presentations required to learn the order of 8 items in their model. Furthermore, our model generalizes better, since it can be used without changes of any parameter for learning order of any number of items, whereas their model requires another 30k episodes of meta-learning when the number of items becomes larger than 9. Apart from that, both models support the same conclusions with regard to support for transitive inference, the symbolic distance effect, the serial position effect, and reconfiguration when separately learnt linear orders are merged into one.

Obviously there exist drastic difference in the required network size (1 neuron versus 200) and learning complexity: Local learning of input weights of a single neuron with a fixed learning rate, where 10 adjustable weights suffice for learning order with 10 items, versus learning of 40k weights of recurrent connections, involving storage and updating of 40k eligibility traces and 40k multiplications of analog numbers, training of 40k meta-parameters through meta-learning in 30k episodes with modified learning rates for different episodes, and training of an artificial neuromodulatory system through meta-learning in their model. These differences in network size and learning complexity imply substantial differences in the implementation cost and energy efficiency of the two models. We are not aware of any neuromorphic implementation of the model of ref. ^21^.

### Reporting summary

Further information on research design is available in the Nature Portfolio Reporting Summary linked to this article.

## Supplementary information

## Data Availability

All data used in the experiments were synthetically generated. The code used to generate these data is publicly available on GitHub at (https://github.com/superrrpotato/Relationship-learning) and archived on Zenodo^53^.

## Code availability

Example code implementing the proposed learning rule, together with the code used to generate all figures, is publicly available on GitHub at (https://github.com/superrrpotato/Relationship-learning) and archived on Zenodo^53^.

## References

[1] Summerfield, C., Luyckx, F. & Sheahan, H. Structure learning and the posterior parietal cortex. *Prog. Neurobiol.* **184**, 101717 (2020). 31669186 10.1016/j.pneurobio.2019.101717

[2] Ganguli, S. et al. One-dimensional dynamics of attention and decision making in LIP. *Neuron* **58**, 15–25 (2008). 18400159 10.1016/j.neuron.2008.01.038 PMC7204626

[3] Fitzgerald, J. K. et al. Biased associative representations in parietal cortex. *Neuron* **77**, 180–191 (2013). 23312525 10.1016/j.neuron.2012.11.014 PMC3545205

[4] Rutishauser, U., Aflalo, T., Rosario, E. R., Pouratian, N. & Andersen, R. A. Single-neuron representation of memory strength and recognition confidence in left human posterior parietal cortex. *Neuron* **97**, 209–220 (2018). 29249283 10.1016/j.neuron.2017.11.029 PMC5754243

[5] Chafee, M. V. A scalar neural code for categories in parietal cortex: representing cognitive variables as “more” or “less”. *Neuron* **77**, 7–9 (2013). 23312511 10.1016/j.neuron.2012.12.025

[6] Bottini, R. & Doeller, C. F. Knowledge across reference frames: cognitive maps and image spaces. *Trends Cogn. Sci.* **24**, 606–619 (2020). 32586649 10.1016/j.tics.2020.05.008

[7] Nieder, A. & Dehaene, S. Representation of number in the brain. *Annu. Rev. Neurosci.* **32**, 185–208 (2009). 19400715 10.1146/annurev.neuro.051508.135550

[8] Roitman, J. D., Brannon, E. M. & Platt, M. L. Representation of numerosity in posterior parietal cortex. *Front. Integr. Neurosci.* **6**, 25 (2012). 22666194 10.3389/fnint.2012.00025 PMC3364489

[9] Nieder, A. et al. The neuronal code for number. *Nat. Rev. Neurosci.* **17**, 366–382 (2016). 27150407 10.1038/nrn.2016.40

[10] Lyons, I. M., Vogel, S. E. & Ansari, D. On the ordinality of numbers: a review of neural and behavioral studies. *Prog. Brain Res.* **227**, 187–221 (2016). 27339013 10.1016/bs.pbr.2016.04.010

[11] Lorenzi, E., Perrino, M. & Vallortigara, G. Numerosities and other magnitudes in the brains: a comparative view. *Front. Psychol.* **12**, 641994 (2021). 33935896 10.3389/fpsyg.2021.641994 PMC8082025

[12] Luyckx, F., Nili, H., Spitzer, B. & Summerfield, C. Neural structure mapping in human probabilistic reward learning. *elife* **8**, e42816 (2019). 30843789 10.7554/eLife.42816 PMC6405242

[13] Viganò, S., Bayramova, R., Doeller, C. F. & Bottini, R. Spontaneous eye movements reflect the representational geometries of conceptual spaces. *Proc. Natl. Acad. Sci.USA* **121**, e2403858121 (2024). 38635638 10.1073/pnas.2403858121 PMC11046636

[14] Xu, B., Wu, J., Xiao, H., Münte, T. F. & Ye, Z. Inferior parietal cortex represents relational structures for explicit transitive inference. *Cereb. Cortex* **34**, bhae137 (2024). 38584088 10.1093/cercor/bhae137 PMC10999362

[15] Di Antonio, G., Raglio, S. & Mattia, M. A geometrical solution underlies general neural principle for serial ordering. *Nat. Commun.* **15**, 8238 (2024). 39300106 10.1038/s41467-024-52240-6 PMC11413371

[16] Mannella, F. & Pezzulo, G. Transitive inference as probabilistic preference learning. *Psychon. Bull. Rev.* **32**, 674–689 (2025). 39438427 10.3758/s13423-024-02600-6

[17] Merritt, D. J. & Terrace, H. S. Mechanisms of inferential order judgments in humans (homo sapiens) and rhesus monkeys (macaca mulatta). *J. Comp. Psychol.* **125**, 227–238 (2011). 21341909 10.1037/a0021572 PMC3398861

[18] Brunamonti, E. et al. Neuronal modulation in the prefrontal cortex in a transitive inference task: evidence of neuronal correlates of mental schema management. *J. Neurosci.* **36**, 1223–1236 (2016). 26818510 10.1523/JNEUROSCI.1473-15.2016 PMC6604826

[19] Nelli, S., Braun, L., Dumbalska, T., Saxe, A. & Summerfield, C. Neural knowledge assembly in humans and neural networks. *Neuron* **111**, 1504–1516 (2023). 36898375 10.1016/j.neuron.2023.02.014 PMC10618408

[20] Lippl, S., Kay, K., Jensen, G., Ferrera, V. P. & Abbott, L. A mathematical theory of relational generalization in transitive inference. *Proc. Natl. Acad. Sci.* **121**, e2314511121 (2024). 38968113 10.1073/pnas.2314511121 PMC11252811

[21] Miconi, T. & Kay, K. Neural mechanisms of relational learning and fast knowledge reassembly in plastic neural networks. *Nat. Neurosci.* **28**, 406–414 (2025). 39814949 10.1038/s41593-024-01852-8

[22] Fried, I. Neurons as will and representation. *Nat. Rev. Neurosci.* **23**, 104–114 (2022). 34931068 10.1038/s41583-021-00543-8 PMC9359715

[23] Sun, W. et al. Learning produces an orthogonalized state machine in the hippocampus. *Nature* **640**, 165–175 (2025). 39939774 10.1038/s41586-024-08548-w PMC11964937

[24] Xiao, Z. et al. Human hippocampal ripples align new experiences with a grid-like schema. *Neuron* **113**, 3661–3672 (2025). 40865535 10.1016/j.neuron.2025.07.028

[25] Li, C. et al. Large memristor crossbars for analog computing. *IEEE International Symposium on Circuits and Systems*1–4 (2018).

[26] Orchard, G. et al. Efficient neuromorphic signal processing with Loihi 2. *IEEE Workshop on Signal Processing Systems* 254–259 (2021).

[27] Okazawa, G., Hatch, C. E., Mancoo, A., Machens, C. K. & Kiani, R. Representational geometry of perceptual decisions in the monkey parietal cortex. *Cell* **184**, 3748–3761 (2021). 34171308 10.1016/j.cell.2021.05.022 PMC8273140

[28] Moyer, R. S. & Bayer, R. H. Mental comparison and the symbolic distance effect. *Cogn. Psychol.* **8**, 228–246 (1976).

[29] De Falco, E., Ison, M. J., Fried, I. & Quian Quiroga, R. Long-term coding of personal and universal associations underlying the memory web in the human brain. *Nat. Commun.* **7**, 13408 (2016). 27845773 10.1038/ncomms13408 PMC5116073

[30] D’Souza, P., Liu, S.-C. & Hahnloser, R. H. Perceptron learning rule derived from spike-frequency adaptation and spike-time-dependent plasticity. *Proc. Natl. Acad. Sci.* **107**, 4722–4727 (2010). 20167805 10.1073/pnas.0909394107 PMC2842046

[31] Roesch, M. R., Esber, G. R., Li, J., Daw, N. D. & Schoenbaum, G. Surprise! Neural correlates of pearce–hall and Rescorla–Wagner coexist within the brain. *Eur. J. Neurosci.* **35**, 1190–1200 (2012). 22487047 10.1111/j.1460-9568.2011.07986.x PMC3325511

[32] Rumelhart, D. E., McClelland, J. L. & Group, P. R. *Parallel Distributed Processing, Volume 1: Explorations in the Microstructure of Cognition: Foundations.* (MIT Press, Cambridge, MA, 1986).

[33] Kumano, H., Suda, Y. & Uka, T. Context-dependent accumulation of sensory evidence in the parietal cortex underlies flexible task switching. *J. Neurosci.* **36**, 12192–12202 (2016). 27903728 10.1523/JNEUROSCI.1693-16.2016 PMC6601986

[34] Sorscher, B., Mel, G. C., Ocko, S. A., Giocomo, L. M. & Ganguli, S. A unified theory for the computational and mechanistic origins of grid cells. *Neuron* **111**, 121–137 (2023). 36306779 10.1016/j.neuron.2022.10.003

[35] Heald, J. B., Wolpert, D. M. & Lengyel, M. The computational and neural bases of context-dependent learning. *Annu. Rev. Neurosci.* **46**, 233–258 (2023). 36972611 10.1146/annurev-neuro-092322-100402 PMC10348919

[36] Bernardi, S. et al. The geometry of abstraction in the hippocampus and prefrontal cortex. *Cell* **183**, 954–967 (2020). 33058757 10.1016/j.cell.2020.09.031 PMC8451959

[37] Courellis, H. S. et al. Abstract representations emerge in human hippocampal neurons during inference. *Nature* **632**, 841–849 (2024). 39143207 10.1038/s41586-024-07799-x PMC11338822

[38] Zhang, Y. et al. Dendritic computation for rule-based flexible categorization. Preprint at 10.1101/2025.06.03.657766 (2025).

[39] Wright, W. J., Hedrick, N. G. & Komiyama, T. Distinct synaptic plasticity rules operate across dendritic compartments in vivo during learning. *Science* **388**, 322–328 (2025). 40245144 10.1126/science.ads4706 PMC12906676

[40] Marder, E. & Goaillard, J.-M. Variability, compensation and homeostasis in neuron and network function. *Nat. Rev. Neurosci.* **7**, 563–574 (2006). 16791145 10.1038/nrn1949

[41] Mozzachiodi, R. & Byrne, J. H. More than synaptic plasticity: role of nonsynaptic plasticity in learning and memory. *Trends Neurosci.* **33**, 17–26 (2010). 19889466 10.1016/j.tins.2009.10.001 PMC2815214

[42] Buzsáki, G. & Mizuseki, K. The log-dynamic brain: how skewed distributions affect network operations. *Nat. Rev. Neurosci.* **15**, 264–278 (2014). 24569488 10.1038/nrn3687 PMC4051294

[43] Gray, R. M. et al. Toeplitz and circulant matrices: a review. *Found. Trends® Commun. Inf. Theory* **2**, 155–239 (2006).

[44] Munoz, F. et al. Learned representation of implied serial order in posterior parietal cortex. *Sci. Rep.* **10**, 9386 (2020). 32523062 10.1038/s41598-020-65838-9 PMC7287075

[45] Kendall, M. G. A new measure of rank correlation. *Biometrika* **30**, 81–93 (1938).

[46] Cortes, C. & Vapnik, V. Support-vector networks. *Mach. Learn.* **20**, 273–297 (1995).

[47] Shalev-Shwartz, S. & Ben-David, S. *Understanding Machine Learning: From Theory to Algorithms.* (Cambridge University Press, Cambridge, 2014).

[48] Hertz, J., Krogh, A. & Palmer, R. G. *Introduction to the Theory of Neural Computation.* (Addison-Wesley, Redwood City, CA, 1991).

[49] Murphy, C., Gray, P. & Stewart, G. Verified perceptron convergence theorem. *Proceedings of the 1st ACM SIGPLAN International Workshop on Machine Learning and Programming Languages,* 43–50 (2017).

[50] Crammer, K., Dekel, O., Keshet, J., Shalev-Shwartz, S. & Singer, Y. Online passive-aggressive algorithms. *J. Mach. Learn. Res.* **7**, 551–585 (2006).

[51] Waydo, S., Kraskov, A., Quiroga, R. Q., Fried, I. & Koch, C. Sparse representation in the human medial temporal lobe. *J. Neurosci.* **26**, 10232–10234 (2006). 17021178 10.1523/JNEUROSCI.2101-06.2006 PMC6674629

[52] Britanak, V., Yip, P. C. & Rao, K. R. *Discrete Cosine and Sine Transforms: General Properties, Fast Algorithms and Integer Approximations.* (Elsevier, Amsterdam, 2010).

[53] Yang, Y. et al. Relationship-learning. *Zenodo*10.5281/zenodo.20729243 (2026).

[54] Gardner, R. J. et al. Toroidal topology of population activity in grid cells. *Nature* **602**, 123–128 (2022). 35022611 10.1038/s41586-021-04268-7 PMC8810387

