# Supplementary material for: The inherent capacity of neurons to learn order relations and support abstract reasoning
Source: Nat Commun. 2026 Aug 5;17:9436. doi: 10.1038/s41467-026-76102-5 (PMC13538631; doi:10.1038/s41467-026-76102-5)
Supplement: Supplementary file 1 [file 41467_2026_76102_MOESM1_ESM.pdf]

Supplementary Information for

The Inherent Capacity of Neurons to Learn Order Relations and Support Abstract Reasoning

A Additional analysis to the Rank Learning Rule

A.1 Rank Learning Rule as local gradient descent

We show that for a fixed pair of items $\mathbf{a}_i$ and $\mathbf{a}_j$ the Rank Learning Rule from Eq. (1) and Eq. (5) is equivalent to performing gradient descent on a corresponding loss function for these two items. This analysis suggests that the Rank Learning Rule makes sense, but makes no implication for the realistic scenario where many different pairs of items, some with overlap, are presented to the learner.

Given a pair of items $\mathbf{a}_i \prec \mathbf{a}_j$ with rank estimates $r(\mathbf{a}_i) = \mathbf{w}\mathbf{a}_i$ and $r(\mathbf{a}_j) = \mathbf{w}\mathbf{a}_j$, and a margin $\delta > 0$, we define the loss function as:

$$L_{i,j} = \begin{cases} \delta - (r(\mathbf{a}_j) - r(\mathbf{a}_i)), & \text{if } (r(\mathbf{a}_j) - r(\mathbf{a}_i)) < \delta, \\ 0, & \text{otherwise.} \end{cases} \tag{38}$$

When calculating the gradient with respect to $\mathbf{w}$, we obtain:

$$\begin{aligned} \frac{\partial L_{i,j}}{\partial \mathbf{w}} &= \frac{\partial L_{i,j}}{\partial (r(\mathbf{a}_j) - r(\mathbf{a}_i))} \frac{\partial (r(\mathbf{a}_j) - r(\mathbf{a}_i))}{\partial \mathbf{w}} \\ &= \begin{cases} -(\mathbf{a}_j - \mathbf{a}_i) & \text{if } (r(\mathbf{a}_j) - r(\mathbf{a}_i)) < \delta, \\ 0 & \text{otherwise.} \end{cases} \\ &= -e(\mathbf{a}_i - \mathbf{a}_j), \end{aligned} \tag{39}$$

where

$$e = \begin{cases} 1, & \text{if } (r(\mathbf{a}_j) - r(\mathbf{a}_i)) < \delta, \\ 0, & \text{otherwise,} \end{cases} \tag{40}$$

is the error signal.

Hence, applying gradient descent for a fixed pair of items results in an update rule that matches Eq. (5):

$$\Delta \mathbf{w} = -\eta \frac{\partial L_{i,j}}{\partial \mathbf{w}} = \eta e(\mathbf{a}_j - \mathbf{a}_i). \tag{41}$$

A.2 Relation to existing loss functions and sensitivity to the margin parameter

The loss function in Eq. (4) is closely related to pairwise ranking objectives used in machine learning, such as hinge-based formulations that approximate the Kendall tau loss [1, 2]; interested readers may refer to Sections 17.2–17.4 of [2]. These objectives enforce the correct ordering between two items by requiring that the score of the higher-ranked item exceeds that of the lower-ranked item by at least a margin.

A notable difference is the margin parameter δ. In our formulation, δ specifies the required separation between the scalar outputs for two ordered items. The resulting update depends only on the difference $(\mathbf{a}_j - \mathbf{a}_i)$ and an error signal indicating whether the margin constraint is violated, allowing the rule to be implemented using locally available variables.

To examine the influence of δ, we performed a sensitivity analysis across different margin values, code dimensions, and levels of orthogonality between item representations. Figure S1A shows the success rate when training is limited to 1000 pair presentations, averaged over 100 runs. Smaller margins generally lead to faster learning. When training continues for sufficiently many iterations, all tested conditions converge to the correct ranking (Fig. S1B).

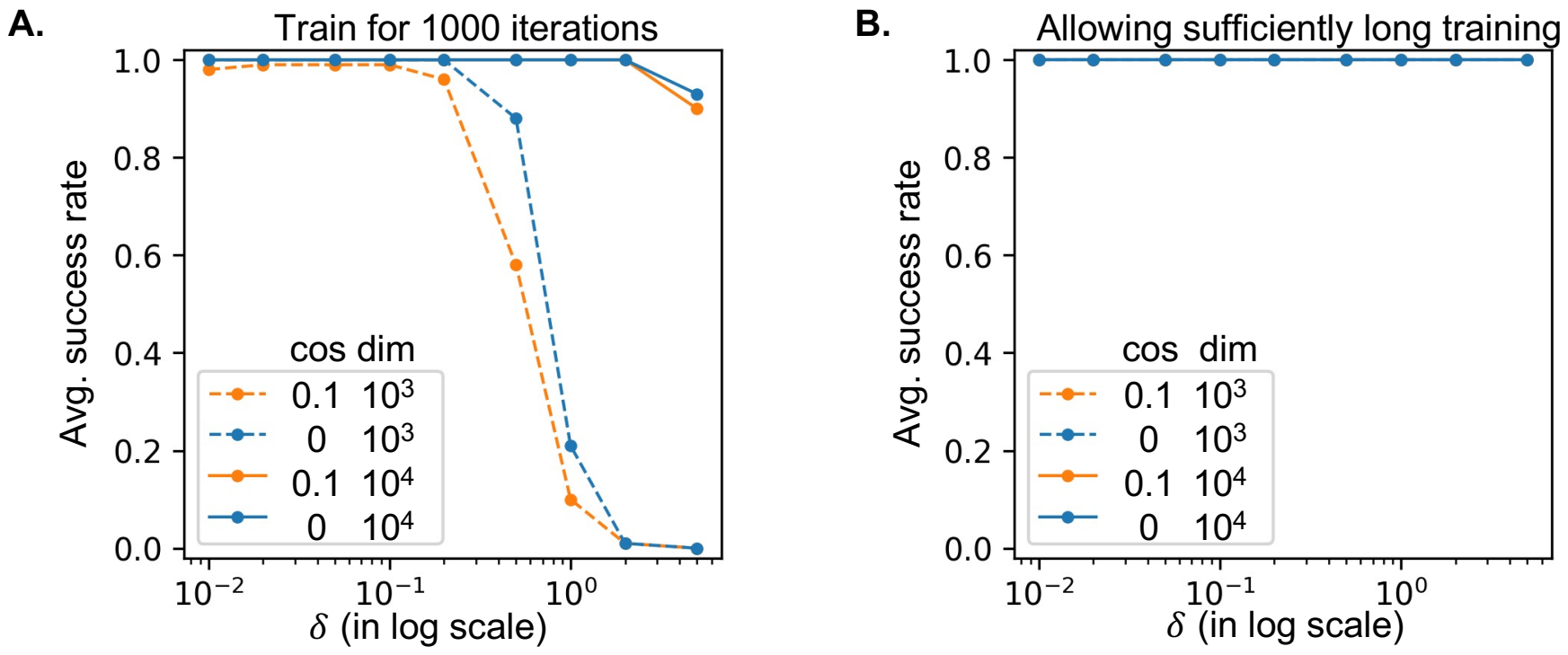

Fig. S1: **Sensitivity analysis for the margin parameter δ. A.** Average success rate for learning a linear ranking of 10 items as a function of δ, under a fixed training budget of 1000 pair presentations. The success rate is defined as in what ratio the correct ranking is learned during 100 independent runs with different random seeds. Smaller values of δ generally lead to higher learning success rates. Learning is also easier in higher-dimensional item codes (10^4 vs. 10^3) and with greater orthogonality between item codes (average cosine similarity 0 vs. 0.1). **B.** Convergence behavior when training is allowed to continue for sufficiently many iterations. Then learning converges for all values of the margin δ and for all conditions to the correct ranking.

B Further details to Sec 2.1

B.1 Impact of relaxed orthogonality of neural codes for items

The brain represents items by assemblies of concept cells, that fire when this item is presented (or mentally considered) [3]. An important question for this study is to what extent these neural presentations of items can be viewed as being represented by orthogonal vectors. These assemblies are sparse (consisting of 0.2% to 1% of neurons in the human medial temporal lobe [4, 5]). But some neurons respond to more than one item. In other words, the assemblies have in general some overlap. The relative size of the overlap between two assemblies of concept cells for two different concepts has been estimated experimentally by the relative frequency that a neuron that responds to one concept also responds to the other concept. More precisely, the overlap between two assemblies is measured as % of neurons in their intersection relative to the size if the union of both assemblies.

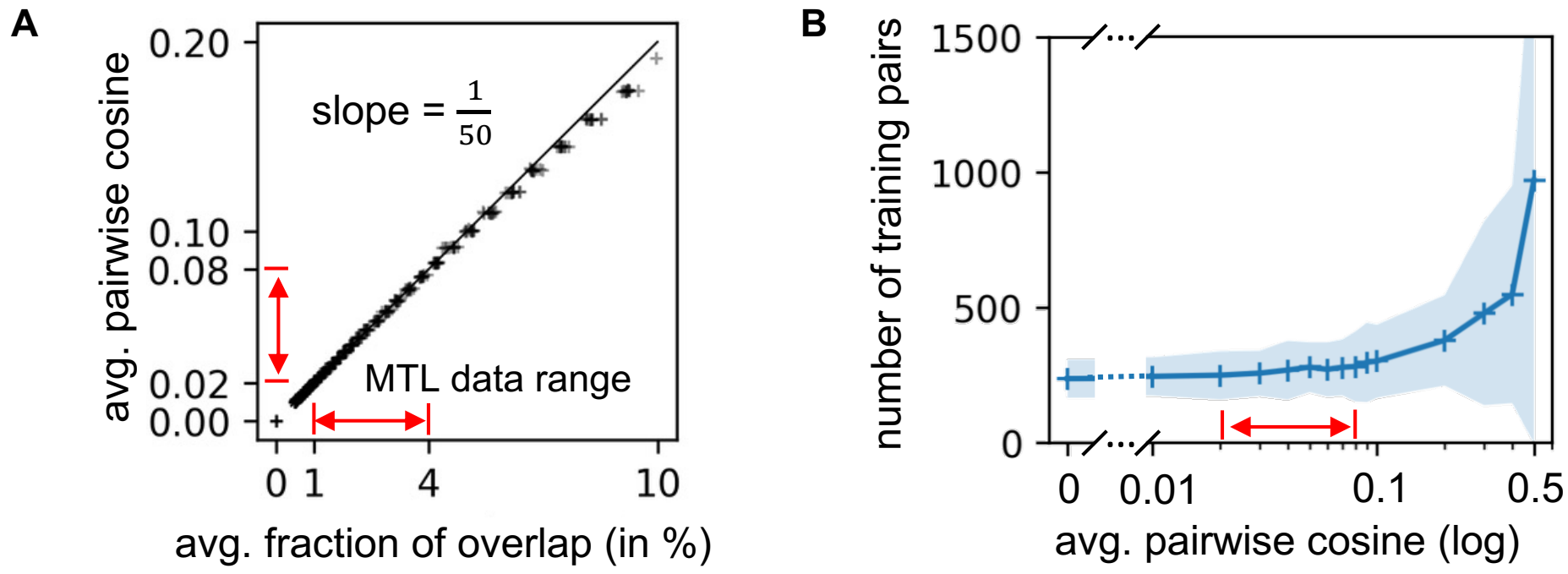

Fig. S2: **A.** The average fraction of overlap and the average pairwise cosine similarity follow a linear relationship in the range of small overlaps that we consider. Data from the human medial temporal lobe (MTL) suggest that the average fraction of overlap between different objects falls within the range of 1% to 4% [3, 5] (marked by the red arrows on the axes, also in panel B). From our simulation results, we observe that this range corresponds to an average cosine similarity between 0.02 and 0.08. A linear regression fit shows that the average cosine similarity is approximately $\frac{1}{50}$ of the average fraction of overlap, as depicted by the black line. **B.** Number of pairs of items presented, required for the agent to reach 100% testing accuracy with varying average cosine similarity, for the task of learning brispiness among 10 items. Lines and shaded areas represent the averages and standard deviations from 100 repeated experiments. Training is relatively fast when the average pairwise cosine is < 0.1, aligning with human MTL data $[0.02, 0.08]$. (Note: The x-axis of **B** is marked with (log), it means the scale of the axis adopts the logarithmic scale. Values shown on its side are still the original values.)

We show in Fig. S2A empirically that the fraction of overlap between assemblies for two different items is (in the range of small overlaps) approximately linearly related to the cosine similarity between the two corresponding binary vectors that encode these assemblies. This empirical analysis was carried out by assigning a binary vector of dimension 10^4 to each assembly (item), with sparseness uniformly sampled from the range of 0.2% to 1% of 1's. In addition, Fig. S2A shows that overlaps between 1 and 4 % yield cosine values between 0.02 and 0.08. The detailed method used to adjust the average fraction of overlap is discussed in Section 4.2.2.

Subsequently, we show in Fig. S2B that the learning speed of our model is for the range of these cosine values between 0.02 and 0.08 almost the same as for perfect orthogonality, i.e. cosine value 0. The task is the same as in Fig. 2B, where a single total order is learned. All settings are the same as depicted in Section 4.2.1.

B.2 Relationship between the number of weight updates in an online learning model and the number of item pairs needed to achieve the same performance in batch training using the Rank Learning Rule

We illustrated the standard online learning approach in Fig. 1A: weights are updated only when there is a "surprise"; otherwise, they remain unchanged. For this online learning rule, we use the number of weight updates (in Fig. 2D and Fig. 3D-E) throughout the paper to evaluate learning speed. However, this measure for online learning differs from the standard measure in batch learning, where one counts the number of training examples (each example consists of a pair of items being compared). In this section we examine empirically the relationship between these two different measures for learning speed for the "brispiness" learning experiment.

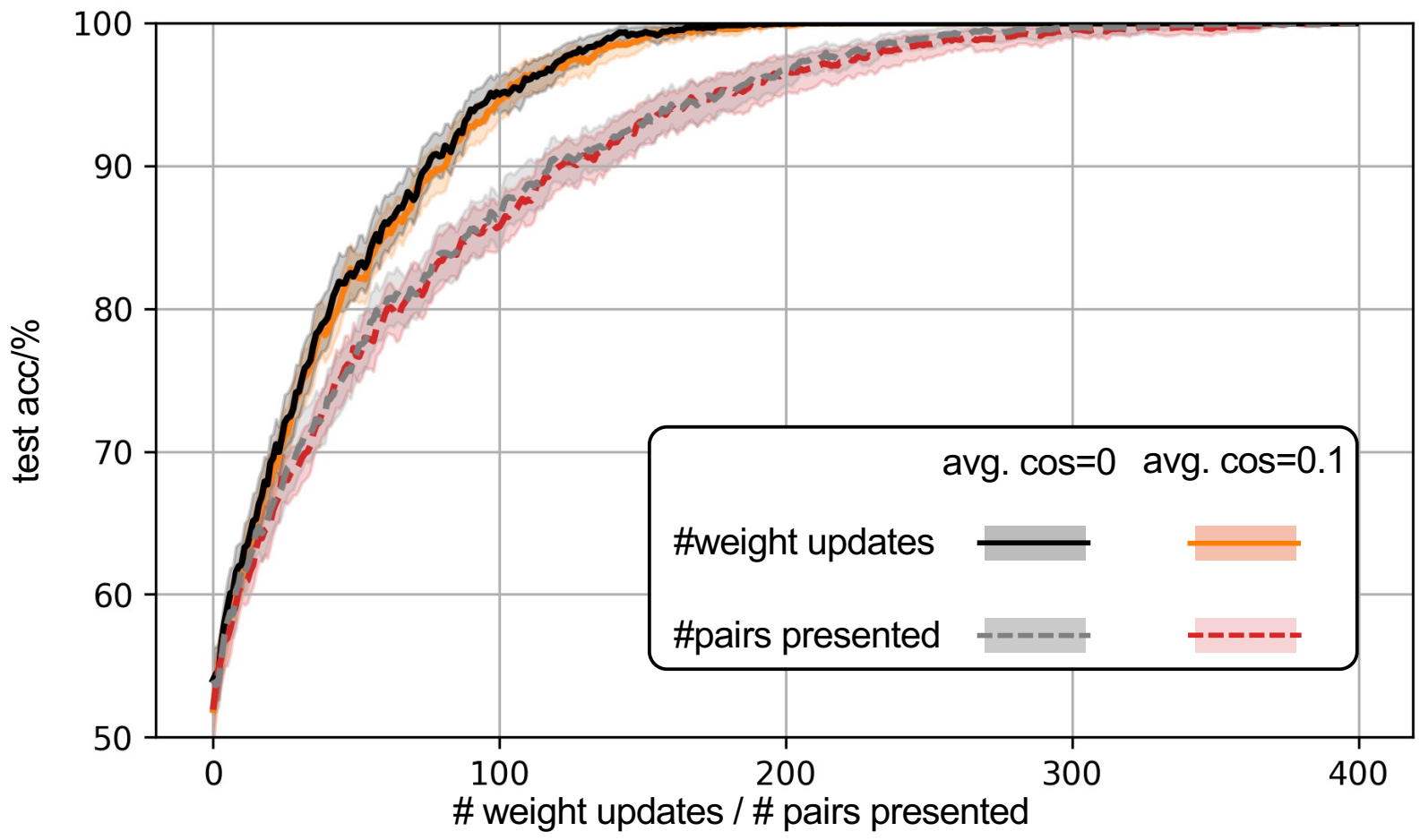

Fig. S3: Relationship between the number of weight updates and the number of pairs presented (shaded area indicates the 99% bootstrap confidence interval). The pairs are accompanied by their relative ranking information, which indicates which item is ranked higher. The comparison is made under conditions of both perfect orthogonality and approximate orthogonality (with an average cosine similarity of 0.1).

From the figure above, it is clear that there is a difference between the two measures. During training from scratch until the results become accurate, only about 55% of the presented pairs trigger weight updates in online learning, meaning that roughly 45% of the pairs do not lead to any update, even when all pairs are presented with equal probability. This indicates that, throughout the training process until accuracy is achieved, weight updates occur for only slightly more than half of the training pairs.

B.3 Learning dynamics for randomly sampled training pairs

In Section 2.1, we include training with both adjacent and non-adjacent pairs. Here we provide additional experimental results.

Figure 2E shows the learning curves in this setting. The model reaches perfect accuracy after fewer training iterations compared with adjacent-pair training. Figure S4 shows the scalar rank estimates at the time when perfect accuracy is first achieved.

We found that training with random pairs leads to faster learning convergence, while the estimated ranks continue to exhibit the same characteristic S-shaped profile observed under adjacent-pair training. This confirms that the qualitative structure of the learned rank representation is robust to the choice of training pairs.

We keep adjacent-pair training as the primary setting in later experiments after Section 2.1 because it isolates transitive generalization. When training is restricted to adjacent pairs, non-adjacent relations are never directly observed, so correct performance requires transitive inference. In contrast, random-pair training provides direct access to long-range relations and reduces the need for inference.

In contrast, when random pairs are used for both training and testing, the learning problem becomes easier for two reasons: (i) transitive inference is no longer required, since long-range relations are directly observed during training; and (ii) the test set necessarily overlaps with, or is statistically dependent on, the training distribution.

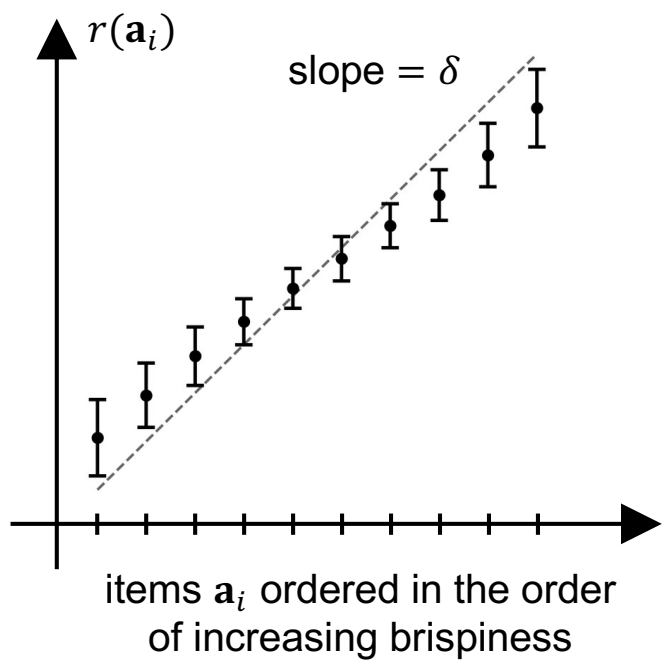

Fig. S4: **Training with randomly sampled item pairs preserves the characteristic S-shaped rank representation.** Rank estimates obtained under random-pair training. Brispiness predictions for all items were evaluated at that stage of learning when perfect accuracy was first achieved. Dots and error bars indicate means and standard deviations, respectively, computed over 100 rounds of training using different random seeds. The resulting rank estimates retain the characteristic S-shaped profile observed under adjacent-pair training in Fig. 2F.

C Further details to Sec 2.4

In order to make the role of the grid-cell layer more transparent, we compare here the learned two ranks with the 2D representation that arises from the PCA of grid-cell responses. As explained in Section 2.4, the outputs of the two rank-estimator neurons already provide for each object a pair of learned rank values, and therefore a location in a 2D relational space. These 2D coordinates are then used as input to the grid-cell model, whose population responses are subsequently projected to two dimensions by PCA.

The result of this comparison is shown in Fig. S5. Panel **A** shows the learned 2D ranking directly, that is, the locations of the objects in the space spanned by the two learned rank estimates. Panel **B** shows the corresponding 2D PCA projection of the grid-cell responses for the same objects. One sees that the relative arrangement of the objects is largely preserved. Hence the grid-cell layer does not create an unrelated geometric structure, but rather transforms the learned 2D relational code into a distributed population code while retaining its underlying geometry.

This comparison clarifies the functional role of the grid-cell stage in our model. The learned rank representation already provides the relevant 2D relational variables, whereas the grid-cell network converts these variables into a population code that can support a cognitive map. Thus the PCA projection of grid-cell responses should be interpreted as a low-dimensional readout of the geometry that is already implicit in the learned pair of rank estimates.

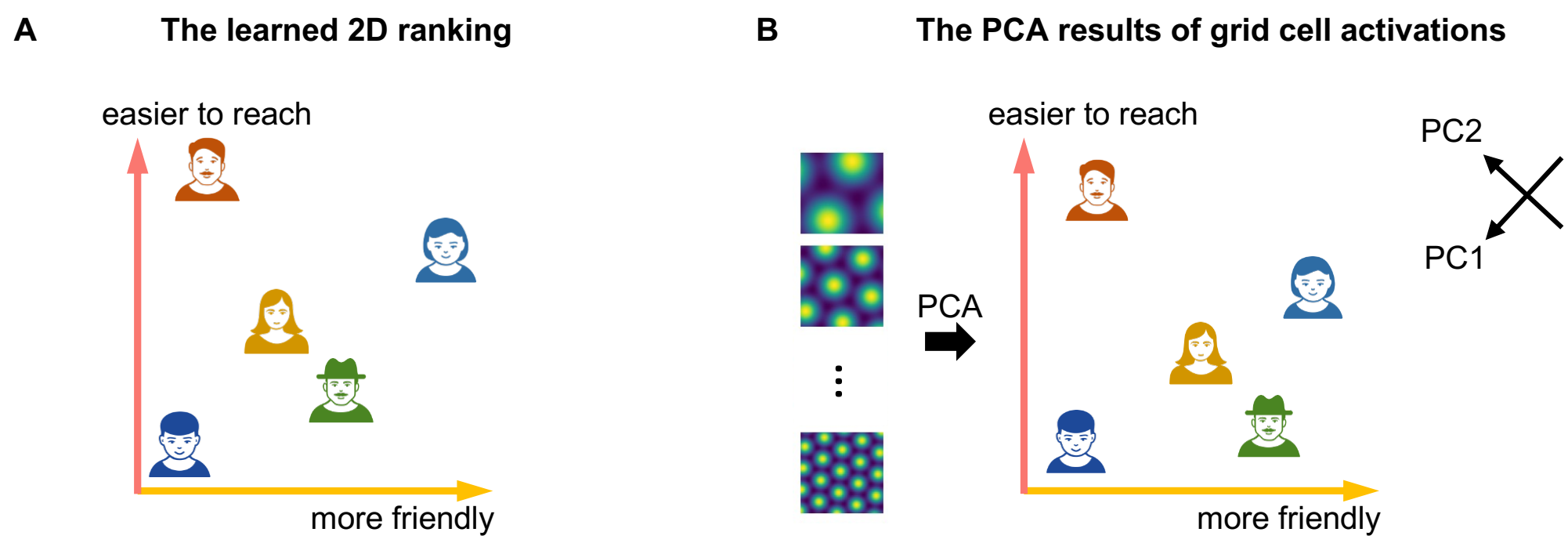

Fig. S5: **Comparison between the learned two ranks and the PCA projection of grid-cell activity. A.** The learned two ranks of the objects, obtained directly from the two learned rank estimates. **B.** The 2D PCA projection of the grid-cell activations for the same objects. The relative arrangement of the objects is largely preserved, showing that the grid-cell population code retains the geometry of the learned two ranks.

D Further details to Section 2.5

D.1 Theoretical analysis

To understand how learning prioritizes the context code $\mathbf{c}$ over the item code $\mathbf{a}$, we analyze how weight updates affect rank estimates.

$$r = \mathbf{w}\mathbf{a} + \mathbf{w}_c\mathbf{c}. \tag{42}$$

After updating the weights according to Eq. (5), and considering different learning rates, we have:

$$\Delta\mathbf{w} = \eta \ e \ (\mathbf{a}_j - \mathbf{a}_i), \quad \Delta\mathbf{w}_c = \eta_c \ e \ (\mathbf{c}_j - \mathbf{c}_i). \tag{43}$$

When the compared objects $\mathbf{a}_i$ and $\mathbf{a}_j$ are from the same context, the second term is zero, indicating that $\mathbf{w}_c$ does not participate in the "train short" phase.

The new rank estimate of objects $\mathbf{a}_i$ and $\mathbf{a}_j$ are:

$$\begin{aligned} r_i' &= [\mathbf{w} + \eta \ e \ (\mathbf{a}_j - \mathbf{a}_i)]\mathbf{a}_i + [\mathbf{w}_c + \eta_c \ e \ (\mathbf{c}_j - \mathbf{c}_i)]\mathbf{c}_i, \\ r_j' &= [\mathbf{w} + \eta \ e \ (\mathbf{a}_j - \mathbf{a}_i)]\mathbf{a}_j + [\mathbf{w}_c + \eta_c \ e \ (\mathbf{c}_j - \mathbf{c}_i)]\mathbf{c}_j. \end{aligned} \tag{44}$$

Because different brain codes are typically orthogonal to each other:

$$\mathbf{a}_i\mathbf{a}_j = \begin{cases} \|\mathbf{a}_i\|_2^2, \text{ if } i = j, \\ \approx 0, \text{otherwise.} \end{cases}, \quad \mathbf{c}_i\mathbf{c}_j = \begin{cases} \|\mathbf{c}_i\|_2^2, \text{ if } i = j, \\ \approx 0, \text{otherwise.} \end{cases} \tag{45}$$

Therefore:

$$\begin{aligned} r_i' &\approx r_i - \eta \ e \ \|\mathbf{a}_i\|_2^2 - \eta_c \ e \ \|\mathbf{c}_i\|_2^2, \\ r_j' &\approx r_j + \eta \ e \ \|\mathbf{a}_j\|_2^2 + \eta_c \ e \ \|\mathbf{c}_j\|_2^2. \end{aligned} \tag{46}$$

When calculating the rank estimate change, the factors affecting the rank estimate changes are:

$$\Delta r \propto \left((\underbrace{\eta\|\mathbf{a}\|_2^2}_{\mathbf{a}'\text{s contribution}} + \underbrace{\eta_c\|\mathbf{c}\|_2^2}_{\mathbf{c}'\text{s contribution}})e \right). \tag{47}$$

The above theoretical analysis clarifies the methods available for prioritizing learning in different representations. Whether learning happens more on the object embedding or context embedding depends on the ratio between:

$$\left(\eta\|\mathbf{a}\|_2^2\right) \text{ v.s. } \left(\eta_c\|\mathbf{c}\|_2^2\right). \tag{48}$$

D.2 Influence of the learning-rate ratio

The figure below shows the model's after-learning behavior for different values of the learning-rate ratio (η_c/η) between the context learning rate η_c and the in-context rank learning rate η. Each panel corresponds to a different ratio.

When the ratio is large, learning mainly updates the context representation. Objects within the same context shift together, so the in-context ordering remains stable.

As the ratio decreases (panels from left to right), updates increasingly act on the item representation. Learning then focuses on satisfying individual boundary training pairs. Because these updates act locally, the relative ordering of objects within the same context can become distorted rather than shifting coherently.

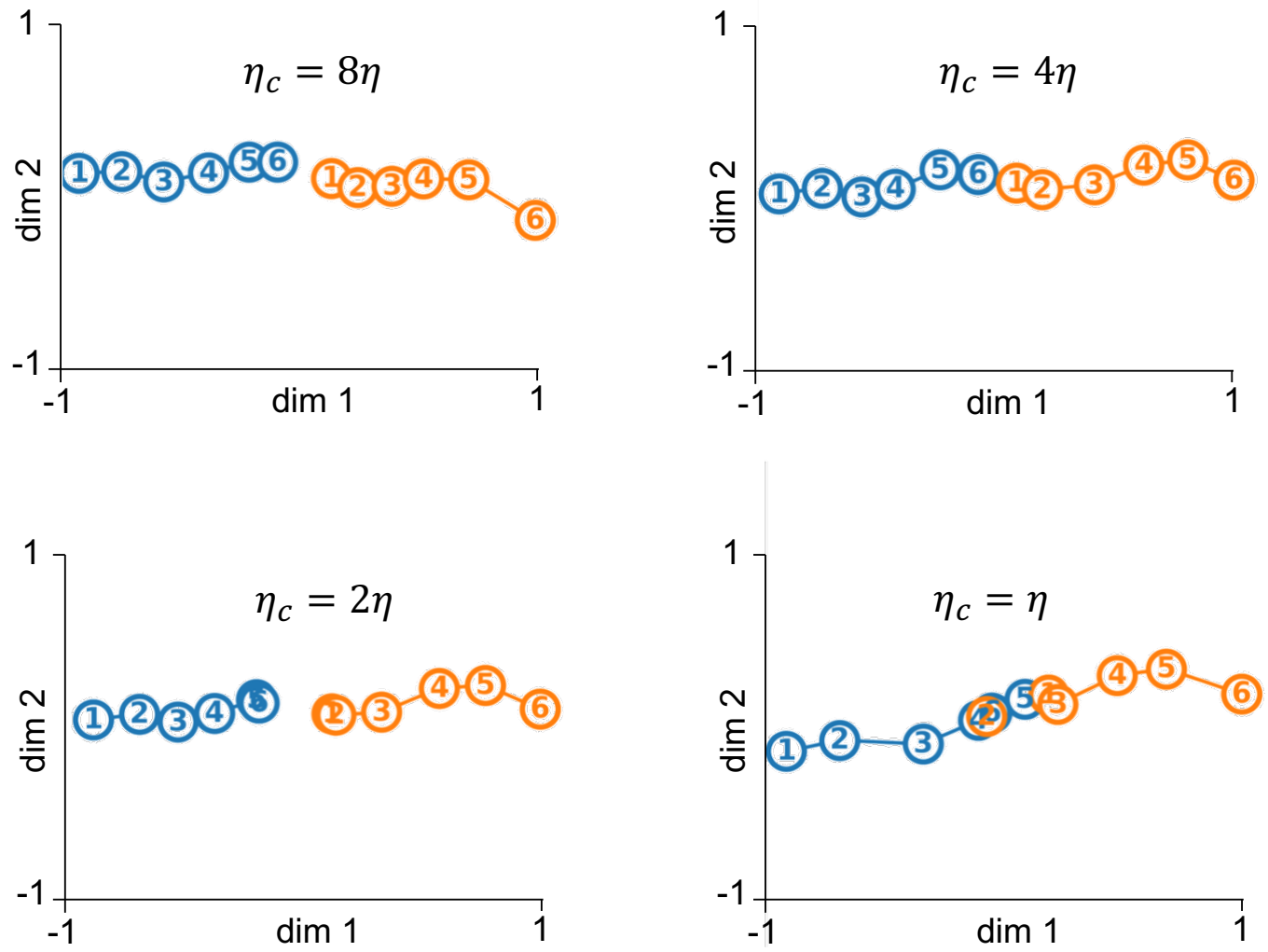

Fig. S6: **Effect of the learning-rate ratio on the learned representation.** Each panel shows the learned positions of objects for a different ratio between the context learning rate η_c and the item learning rate η. Blue and orange points represent objects from two different contexts, and the numbers indicate their rank positions. When the ratio η_c/η is large, updates mainly modify the context representation, so objects within the same context shift together while preserving their internal order. As the ratio decreases, learning increasingly acts on the item representation, which introduces local adjustments driven by the boundary training pairs and can distort the ordering within each context.

To further examine how strongly the model depends on this separation of learning rates, we carried out a systematic sensitivity analysis in which the ratio between the context learning rate η_c and the item learning rate η was varied over a wide range.

For each value of η_c/η, the complete two-phase training protocol was repeated using 100 independent random initializations. A run was counted as successful if the model correctly linked the two contexts after training on the boundary pair while preserving the internal rank structure within each context.

Figure S7 shows the resulting success rate as a function of the learning-rate ratio. Performance improves rapidly as η_c/η increases and approaches perfect reliability once the context learning rate exceeds the item learning rate by roughly an order of magnitude. This indicates that the mechanism does not rely on precise parameter tuning but only requires that context updates occur sufficiently faster than updates of the item weights.

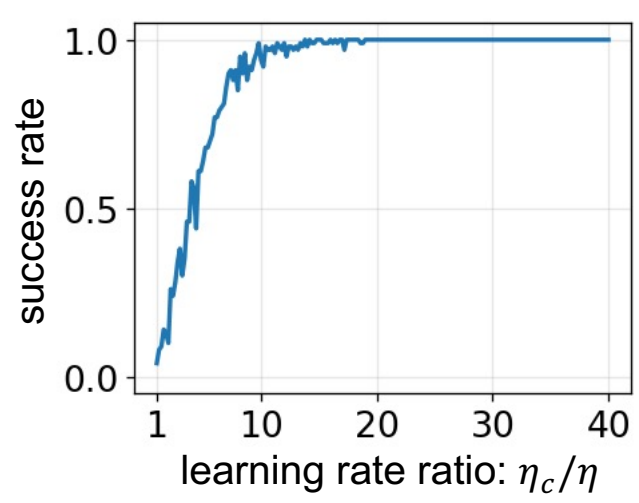

Fig. S7: **Sensitivity of learning performance to the ratio between context and item learning rates.** The plot shows the learning success rate as a function of the ratio between the context learning rate and the item learning rate, η_c/η. Each point shows the fraction of successful runs averaged over 100 independent simulations trained from scratch with different random seeds in both phase 1 (train short) and phase 2 (train long). Successful learning requires the context weights to adapt faster than the item weights, but performance is otherwise robust across a broad range of ratios.

D.3 Extension to three separately learned sets

The same learning principle extends directly from two sets to three sets if each set is assigned a distinct context code **c**. Learning again proceeds in two phases. In the first phase, training examples consist only of adjacent item pairs within the same context. This leads to three separately learned internal rank representations, one for each context. In the second phase, boundary pairs between neighboring contexts are introduced. In the case of three sets, two such boundary pairs suffice to merge the three separately learned ranks into a single coherent order.

The result of this extension is shown in Fig. S8. Panel **A** illustrates the experimental design. Three separate linear orders are first learned under three different contexts. During *train short*, only adjacent item pairs from the same context are presented. During *train long*, two boundary pairs are added in order to link the three sets into one extended rank. Panels **B**–**D** show the corresponding MDS plots, and panels **E**–**G** show the corresponding representational dissimilarity matrices. One sees that after *train short*, the three contexts are represented separately, whereas during *train long* the internal representation reorganizes into a joint structure. After *train long*, the learned representation reflects a single integrated order across all three sets.

Hence the mechanism that we proposed for rapid reconfiguration of separately learned orders is not restricted to two contexts. The same context-dependent learning rule also supports the integration of a larger number of separately learned sets, provided that suitable boundary relations between them are given.

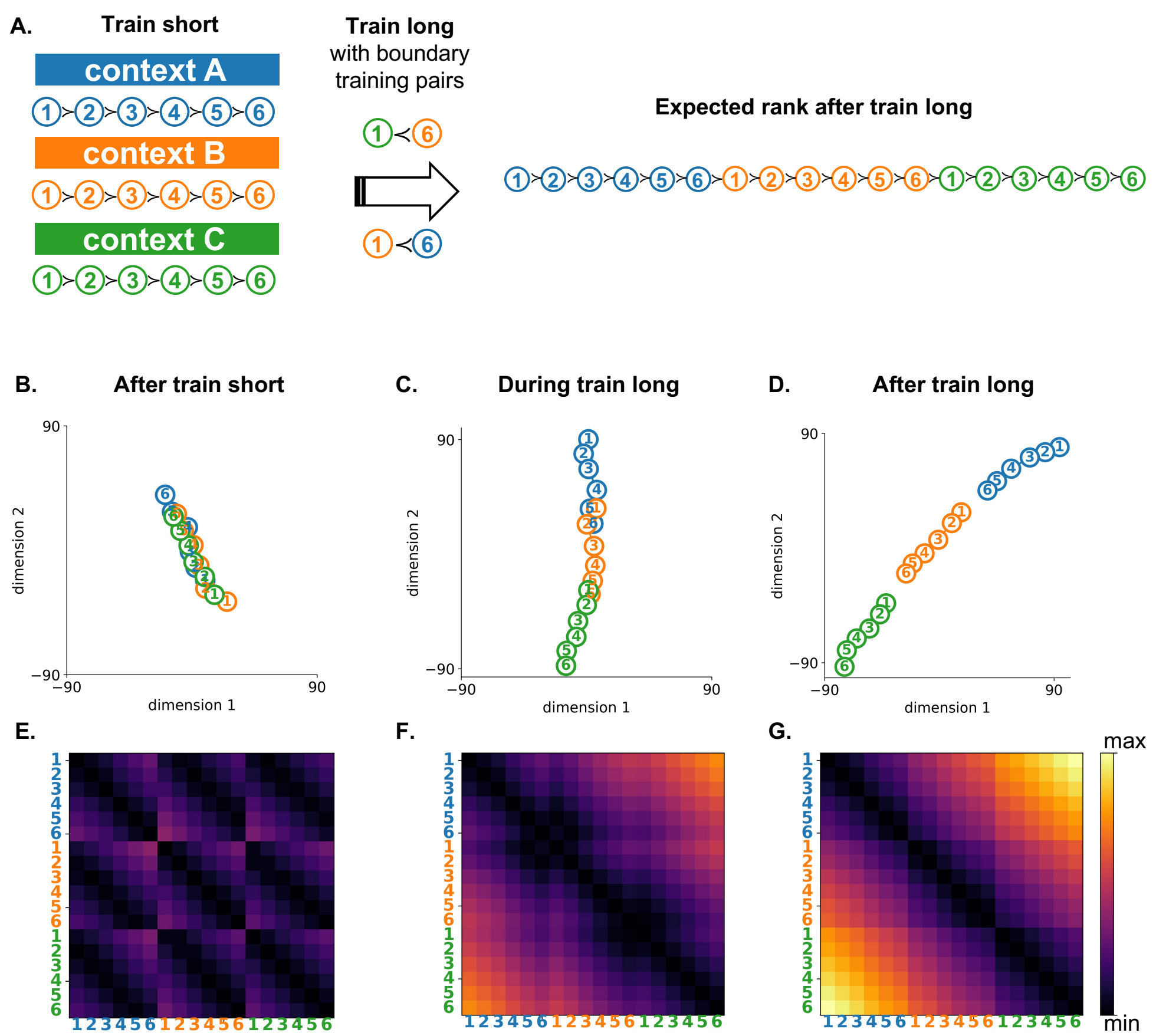

Fig. S8: **Extension of the rank-learning algorithm to contextual learning across three sets. A.** Experimental design. Three separate ranks are defined under three distinct contexts. During the first phase (*train short*), the algorithm is exposed only to adjacent item pairs within the same context. In the second phase (*train long*), two boundary pairs are introduced to link the three sets into a single extended rank. **B**–**D.** MDS plots of the learned representations after *train short*, during *train long*, and after *train long*. **E**–**G.** Corresponding representational dissimilarity matrices.

E Further details to Section 2.6

E.1 Neural network architecture that leads to the emergence of soft rank selective neurons

We show here the architecture that was indicated in Fig. 4A in more detail.

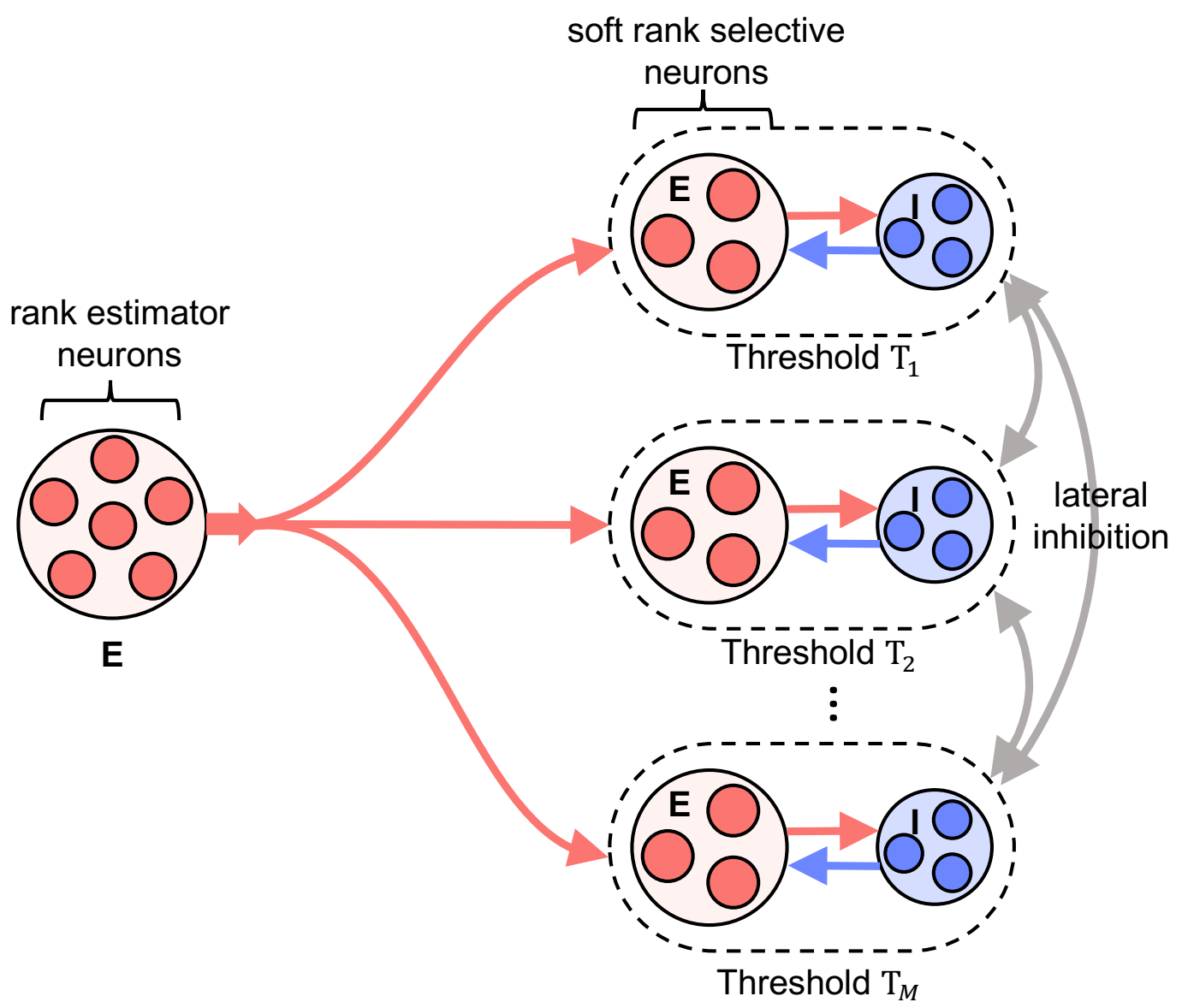

Fig. S9: A population of rank estimator neurons (summation coding neurons) projects to various populations of other neurons. Each population consists of interconnected excitatory and inhibitory neurons, with distinct firing thresholds for excitatory neurons in different populations. Inputs below the threshold value for a population do not activate it. When the inputs exceed the threshold, excitatory neurons are activated, but their firing activity is curtailed by negative feedback from inhibitory neurons. Inhibitory neurons may also provide lateral inhibition between populations in order to ensure that the overlaps in their tuning curves are limited.

E.2 Robustness analysis of of the U-shaped representation

We examine how the qualitative structure of the representation depends on three factors: the number of objects, the level of noise, and the population size of soft rank-selective neurons. To do this, we repeat the simulations while varying one factor at a time and keeping all other parameters fixed. We then analyze the resulting representations using the same dimensionality reduction method as in the main text.

First, Fig. S10 examines how the projected representations evolve as the number of objects increases. We find that the characteristic U-shaped geometry is preserved as object number grows, with a gradual fine-graded sampling on the manifold rather than a qualitative breakdown, indicating that the model scales smoothly to larger set sizes. Second, Fig. S11 evaluates the robustness of the model under increasing noise levels. Even with substantial perturbations, the ordering and low-dimensional structure of the representations remain stable. The effect of noise also helps explain why the decoded geometry in noisy cortical circuits often deviates from a perfect parabola [6, 7]. Finally, Fig. S12 shows how the geometry depends on the number of soft rank-selective neurons contributing to the encoding of the same rank. Varying the population size changes the shape of the code but does not eliminate the U-shaped structure, demonstrating that the effect does not rely on a finely tuned or minimal number of neurons.

Together, these additional results show that the model remains robust to noise, scales gracefully with increasing numbers of objects, and does not depend on a specific or fragile choice of population size.

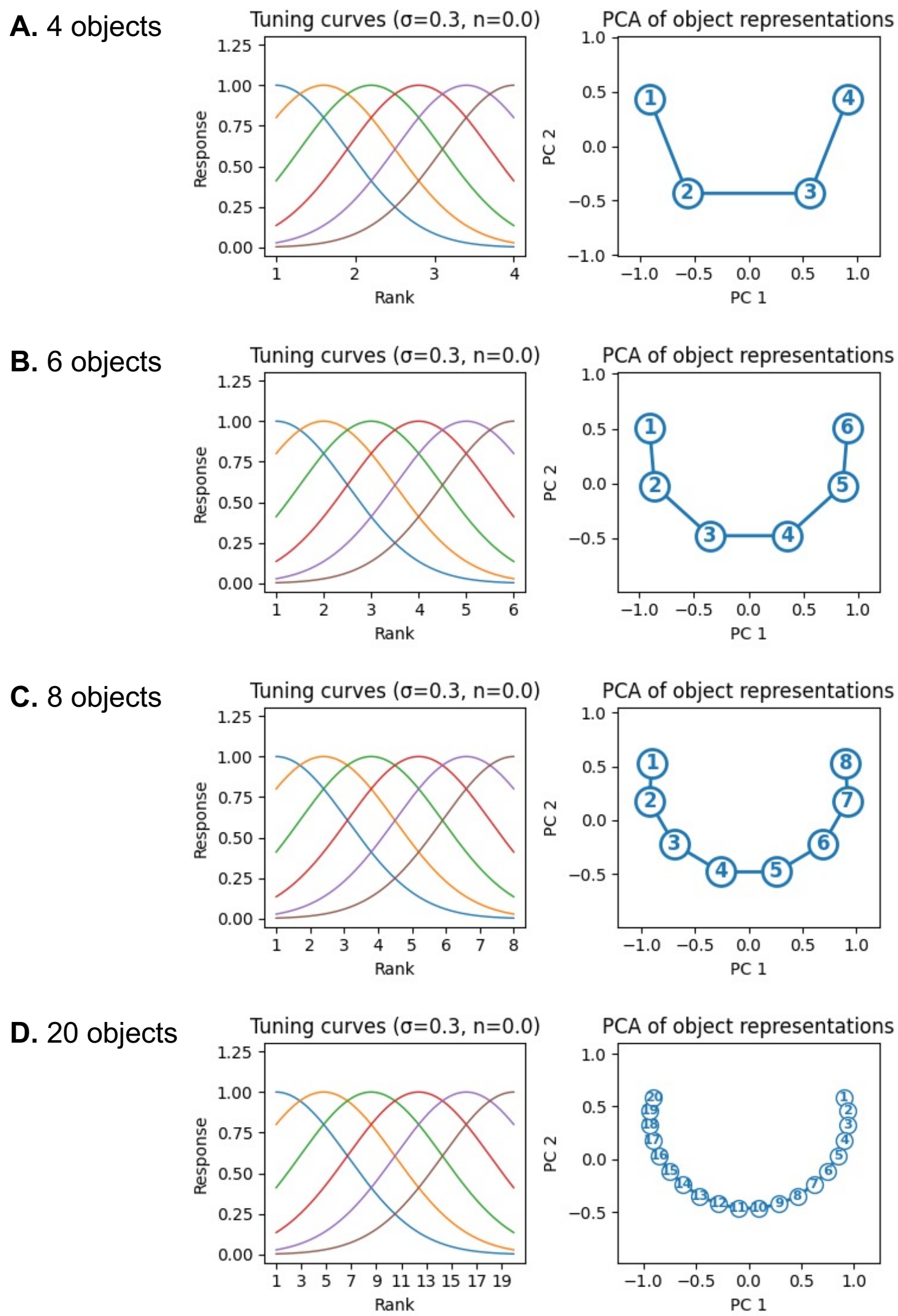

Fig. S10: **Effect of increasing the number of objects on the geometry of object representations.** Panels A–D correspond to $N = 4$, 6, 8, and 20 objects, respectively. Left subpanels show the Gaussian tuning curves of soft rank-selective neurons as a function of object rank, with fixed normalized tuning width ($\sigma = 0.3$) and no noise. Right subpanels show the first two principal components (PC1–PC2) of the resulting object representations, with each point labeled by object rank and connected in rank order. As the number of objects increases, the projected representations preserve a characteristic U-shaped manifold, which gradually becomes more finely sampled rather than collapsing or fragmenting. This indicates that the low-dimensional structure induced by the population code scales smoothly with object number.

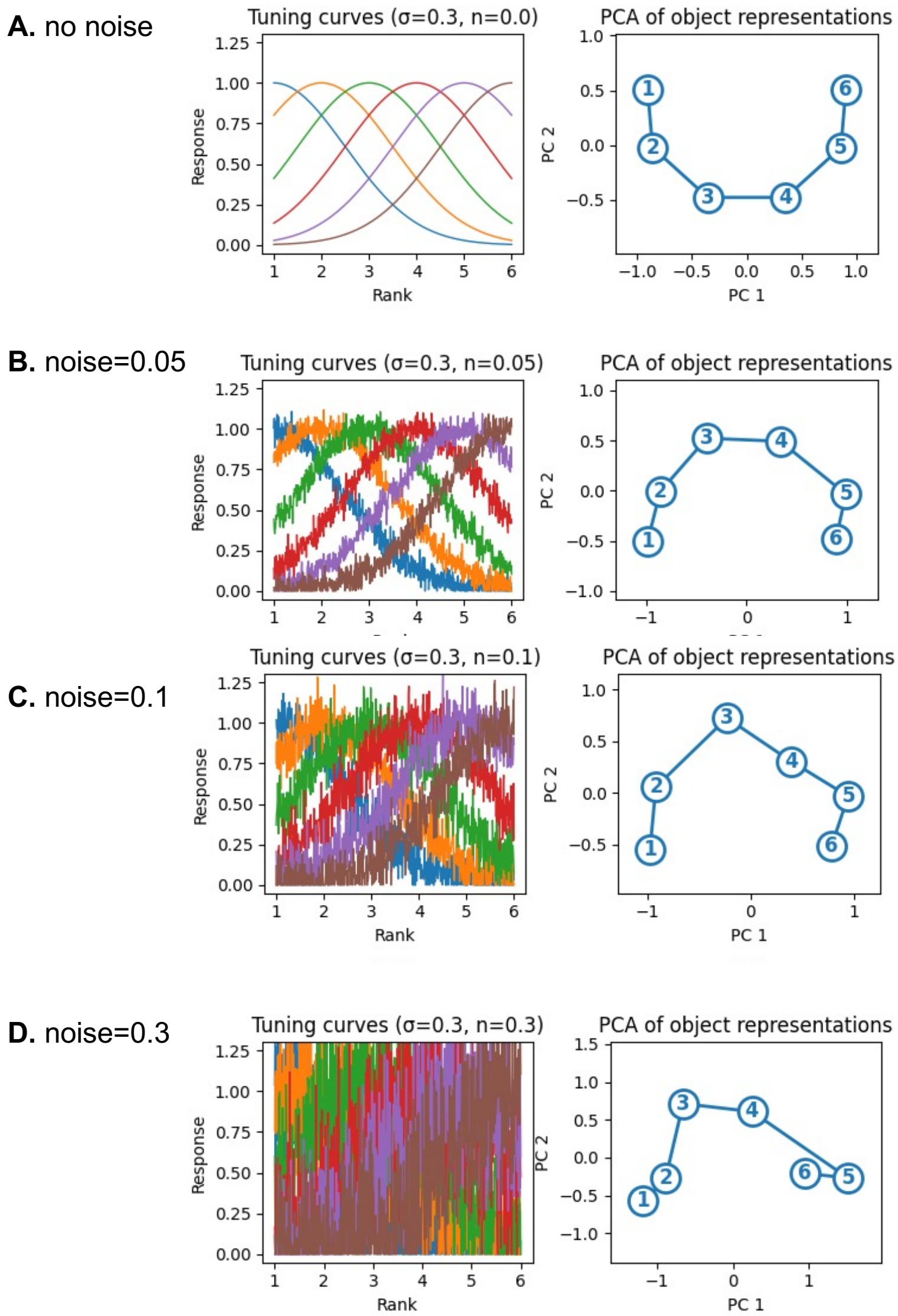

Fig. S11: **Robustness of the model to increasing noise levels.** Panels A–D correspond to noise levels $n = 0$, 0.05, 0.1, and 0.3, respectively. An i.i.d Gaussian noise $\mathcal{N}(0, n)$ is added onto the tuning curves. Left subpanels show the tuning curves of soft rank-selective neurons with additive Gaussian noise applied to neuronal responses, while right subpanels show the corresponding PCA projections of object representations. Despite substantial perturbations at higher noise levels, the relative ordering of objects and the overall U-shaped geometry in the low-dimensional projection are preserved, with distortions increasing gradually rather than abruptly. The effect of noise also helps explain why the decoded geometry in noisy cortical circuits often deviates from a perfect parabola [6, 7].

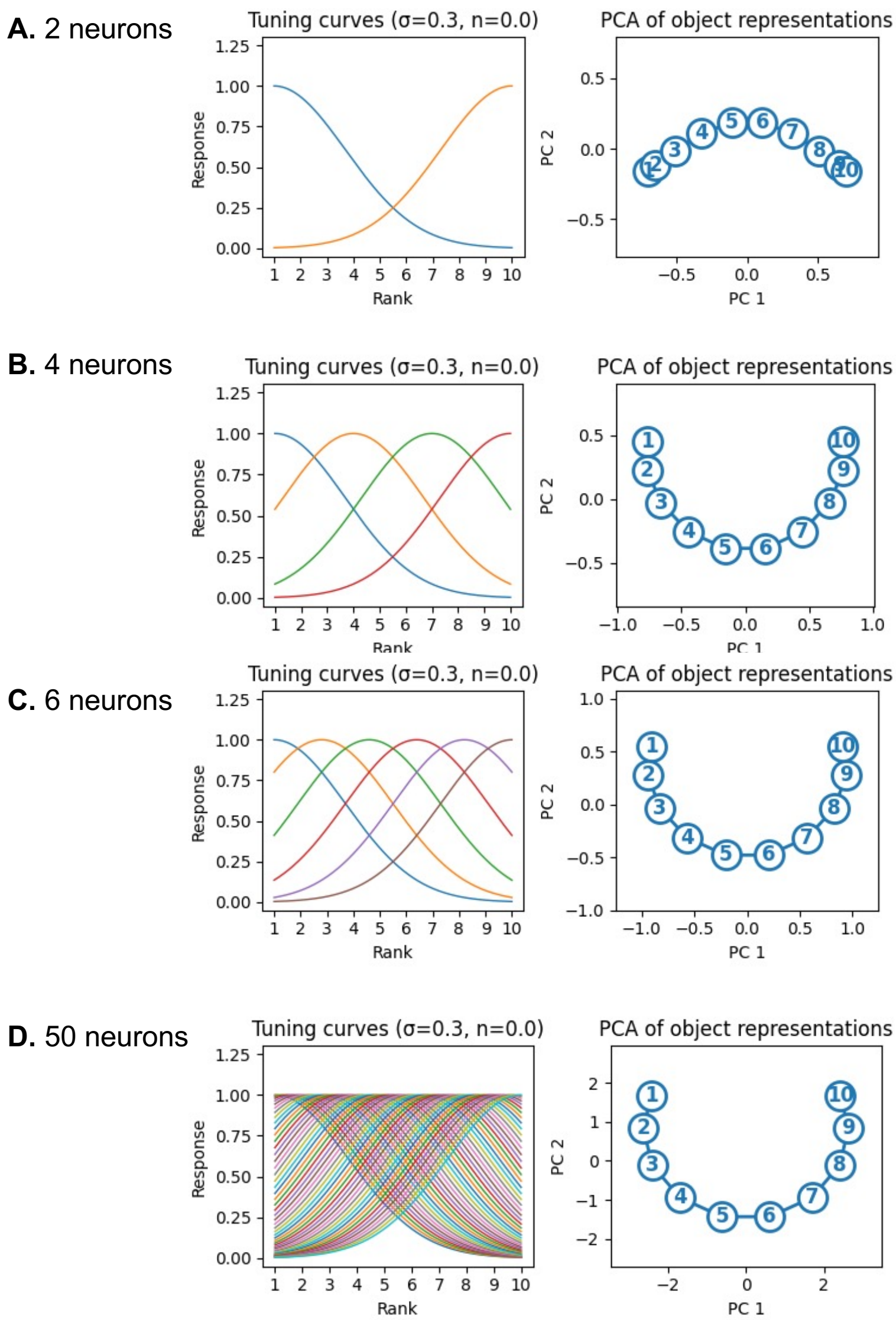

Fig. S12: **Effect of varying the number of soft rank-selective neurons encoding the same set of objects.** Panels A–D correspond to $M = 2$, 4, 6, and 50 neurons, respectively. Left subpanels show the corresponding tuning curves as a function of rank, illustrating increasing overlap and redundancy as the population size grows. Right subpanels show the PCA projections of object representations. While the shape of the code changes with different neuron number, the qualitative U-shaped geometry of the projected representations remains stable across conditions. This demonstrates that the observed structure does not rely on a finely tuned or minimal population size of the soft rank-selective neurons.

F Proof of Validity for the Eigenvector Approximations in Section 4.6

We need to show that:

$$(\mathbf{C}\mathbf{u}_k)[i] = \lambda_k \mathbf{u}_k[i]. \tag{49}$$

Using the Toeplitz structure:

$$(\mathbf{C}\mathbf{u}_k)[i] = \sum_{j=1}^{M} \mathbf{C}_{ij}\mathbf{u}_k[j] = \sum_{n=1}^{M} c(|i-n|)\mathbf{u}_k[n]. \tag{50}$$

Substitute $\mathbf{u}_k[n]$:

$$(\mathbf{C}\mathbf{u}_k)[i] = \sum_{n=1}^{M} c(|i-n|)\cos\left(\frac{\pi k(2n+1)}{2M}\right). \tag{51}$$

Let $m = n - i$. Then $n = i + m$, and m ranges from $-(i-1)$ to $M - i$. Substituting:

$$(\mathbf{C}\mathbf{u}_k)[i] = \sum_{m=-(i-1)}^{M-i} c(|m|)\cos\left(\frac{\pi k(2(i+m)+1)}{2M}\right). \tag{52}$$

Use the Trigonometric identity:

$$\cos(A+B) = \cos A\cos B - \sin A\sin B. \tag{53}$$

Set $A = \frac{\pi k(2i+1)}{2M}$ and $B = \frac{\pi k(2m)}{2M}$, so:

$$\begin{aligned}\cos\left(\frac{\pi k(2(i+m)+1)}{2M}\right) &= \cos\left(\frac{\pi k(2i+1)}{2M}\right)\cos\left(\frac{\pi k(2m)}{2M}\right)\\ &\quad - \sin\left(\frac{\pi k(2i+1)}{2M}\right)\sin\left(\frac{\pi k(2m)}{2M}\right).\end{aligned} \tag{54}$$

Substitute this back:

$$\begin{aligned}(\mathbf{C}\mathbf{u}_k)[i] &= \cos\left(\frac{\pi k(2i+1)}{2M}\right)\sum_{m=-(i-1)}^{M-i} c(|m|)\cos\left(\frac{\pi k(2m)}{2M}\right)\\ &\quad - \sin\left(\frac{\pi k(2i+1)}{2M}\right)\sum_{m=-(i-1)}^{M-i} c(|m|)\sin\left(\frac{\pi k(2m)}{2M}\right).\end{aligned} \tag{55}$$

Since $c(|m|)$ is an even function:

- $\cos\left(\frac{\pi k(2m)}{2M}\right)$ is even, so the sum involving it is nonzero.
- $\sin\left(\frac{\pi k(2m)}{2M}\right)$ is odd, so the sum involving it is zero:

$$\sum_{m=-(i-1)}^{M-i} c(|m|)\sin\left(\frac{\pi k(2m)}{2M}\right) = 0. \tag{56}$$

Thus:

$$(\mathbf{C}\mathbf{u}_k)[i] = \cos\left(\frac{\pi k(2i+1)}{2M}\right)\sum_{m=-(i-1)}^{M-i} c(|m|)\cos\left(\frac{\pi k(2m)}{2M}\right). \tag{57}$$

Assume M is large and i is not near the edges. Approximate the sum over all m from $-\infty$ to ∞:

$$(\mathbf{C}\mathbf{u}_k)[i] \approx \cos\left(\frac{\pi k(2i+1)}{2M}\right)\sum_{m=-\infty}^{\infty} c(|m|)\cos\left(\frac{\pi k(2m)}{2M}\right). \tag{58}$$

Define the part without index i as a constant λ_k:

$$\lambda_k = \sum_{m=-\infty}^{\infty} c(|m|) \cos\left(\frac{\pi k(2m)}{2M}\right). \tag{59}$$

We have:

$$(\mathbf{C}\mathbf{u}_k)[i] = \lambda_k \mathbf{u}_k[i]. \tag{60}$$

This shows that $\mathbf{u}_k$ is an eigenvector of $\mathbf{C}$, with eigenvalue λ_k.

References

[1] Kendall, M. G. A new measure of rank correlation. *Biometrika* **30**, 81–93 (1938).

[2] Shalev-Shwartz, S. & Ben-David, S. *Understanding Machine Learning: From Theory to Algorithms* (Cambridge University Press, Cambridge, 2014).

[3] De Falco, E., Ison, M. J., Fried, I. & Quian Quiroga, R. Long-term coding of personal and universal associations underlying the memory web in the human brain. *Nature communications* **7**, 13408 (2016).

[4] Waydo, S., Kraskov, A., Quiroga, R. Q., Fried, I. & Koch, C. Sparse representation in the human medial temporal lobe. *Journal of Neuroscience* **26**, 10232–10234 (2006).

[5] Fried, I. Neurons as will and representation. *Nature Reviews Neuroscience* **23**, 104–114 (2022).

[6] Nelli, S., Braun, L., Dumbalska, T., Saxe, A. & Summerfield, C. Neural knowledge assembly in humans and neural networks. *Neuron* **111**, 1504–1516 (2023).

[7] Okazawa, G., Hatch, C. E., Mancoo, A., Machens, C. K. & Kiani, R. Representational geometry of perceptual decisions in the monkey parietal cortex. *Cell* **184**, 3748–3761 (2021).
