# Supplementary material for: The inherent capacity of neurons to learn order relations and support abstract reasoning
Source: Nat Commun. 2026 Aug 5;17:9436. doi: 10.1038/s41467-026-76102-5 (PMC13538631; doi:10.1038/s41467-026-76102-5)
Supplement: Supplementary file 2 [file 41467_2026_76102_MOESM2_ESM.pdf]

nature portfolio

Peer Review File

The Inherent Capacity of Neurons to Learn Order Relations and Support Abstract Reasoning

Corresponding Author: Professor Wolfgang Maass

This file contains all reviewer reports in order by version, followed by all author rebuttals in order by version.

Attachments originally included by the reviewers as part of their assessment can be found at the end of this file.

Version 0:

Reviewer comments:

Reviewer #1

(Remarks to the Author)
This paper presents a simple, biologically plausible model of rank order learning in a neural system. The basic idea is to linearly project high-dimensional codes to an approximately one-dimensional code that respects rank-order relations. This can explain some forms of transitive inference, cognitive map formation, and curvature properties of neural representations. Importantly, the authors present rigorous mathematical proofs of convergence.

Overall, I thought this was an interesting and well-written paper. It was hard for me to tell exactly how ground-breaking it is because there's not really a head-to-head comparison with alternative models in terms of explanations and predictions (see below). I think the learning theory is valuable, an aspect that goes beyond previous work in this area.

Comments:

The biological plausibility of the context learning rule is unclear to me.

The authors make a good case for some of the computational advantages of their approach relative to that of Nelli et al. (2023). I'm wondering whether these models also make different empirical predictions? Same question for the modeling of Lippl et al. (2024).

The model doesn't directly predict the symbolic distance effect becaused it lacks a mapping to response time, and lacks a representation of noise that presumably is needed to explain the effect. I don't think it's necessary to show this in a revision, but the authors could clarify what is needed to show this.

At the end of the Discussion, the authors suggest that this form of learning rule is relevant for energy-efficient devices. Because they have not demonstrated energy efficiency, this claim is not well validated.

Typos:
"experimental show" -> "experiments show"
"this simple arguments" -> "this simple argument"
"a 2D cognitive maps" -> "a 2D cognitive map"
"configuration of internal" -> "configuration of internal codes"

(Remarks on code availability)

Reviewer #2

(Remarks to the Author)
This work computationally studied the neural mechanisms by which the brain learns order relationships (like ranks) of multiple objects and uses them for reasoning, typically transitive inference. The authors proposed a simple Rank Learning

Rule, which enables single neurons in a feedforward network to adjust their afferent connections when they make an error in predicting the order of paired items. The authors proved mathematically that this rule always converges to a correct solution within finite learning steps when the neurons are informed of the order relationships between some pairs sampled from multiple items. Their model explains well-known effects in human and animal reasoning, such as the symbolic distance effect (easier comparisons for items far apart) and the terminal item effect (better performance for items at the ends of a sequence). The model also explains why neural representations for rank order projected onto the two-dimensional space spanned by the leading principal components often look U-shaped rather than straight in experiments.

Overall, the work suggests an interesting possibility that neurons have an inherent ability to learn and represent order relationships, supporting abstract reasoning without requiring complex deep learning. The authors also claimed that their findings have implications both for neuroscience and for building more efficient neuromorphic hardware. However, this reviewer considers that several points should be clarified before the manuscript is accepted.

Major comments:

1. At ll. 89-93: In the introduction, the authors stated, "We then address experimental data which show how the brain integrates information from different learnt order relations into a cognitive map (Xiao et al., 2025), or into a larger order (Nelli et al., 2023). We are not aware of any model for the former, and only of a multi-layer perceptron model trained through stochastic gradient descent with different learning rates for each pair for the latter." If my understanding is correct, Miconi and Kay (Nat Neurosci, 2025) proposed another neural mechanism that can integrate shorter relational representations into a larger representation. The above statements need revision.

2. On page 4: The output firing rate of each LIP neuron, r(a_i), should be defined explicitly near Equation 1. Is r(a_i) given as the inner product w*a_i?

3. Fig. S1: The results shown in the figure seem to compare simulation results and experimental data. These data should be clearly indicated in the figure itself or the legend.

4. On ll. 244-256: The authors compared the convergence of their learning rule with that of the Perceptron convergence theorem. However, the form of their Rank Learning Rule more resembles the classic Competitive Learning Rule, which also converges on the surface of a high-dimensional sphere due to weight renormalization following weight update. What is a surprise if the convergence of the Rank Learning Rule is compared with that of the Competitive Learning Rule?

5. On ll. 299-303: Phases 1 and 2 of learning are called "train short" and "train long" in the next paragraph. However, this writing is confusing as these names are not defined before their use.

6. On ll. 318-322: It was difficult for me to understand the definition of Contextual Learning Rule given in Equation 3. Please explain it more clearly.

7. On ll. 323-324: The authors used an 8 times larger rate for learning the context weights compared to learning rank weights. Is this fast learning of context essential for the model's performance? What happens if the two learning rates are comparable? Please explain these points in more detail.

8. On ll. 330-331: The authors mentioned "One sees in Fig. 4E that in general 2 presentations of the boundary pair suffice for adjusting all rank estimates for the long order." The reason was not immediately clear to me. Please explain why learning the boundary pair is sufficient in the proposed model.

9. Fig. 5: To account for the U-shaped 2-dimensional representations, the authors assumed an array of readout neurons with staggered firing thresholds. Theoretically, the hypothesis sounds interesting. However, considering the highly noisy features of cortical circuits, decisions by such an array of neurons may not be robust enough, especially when the number of objects becomes large. Is there any evidence for neurons with staggered thresholds? How does the U-shape of the projected representations behave in the model when the number of objects is increased? Are the errors generated by the model consistent with those observed in experiments?

10. On ll. 332-340: The authors compared their model with a deep network by Neilli et al. (2023), which can also combine separately learned orders into a long representation. In a recent paper, Miconi and Kay (Nat Neurosci, 2025) proposed another mechanism to integrate shorter relational representations into a larger one. The paper is cited in the manuscript, but the authors are asked to argue the differences and similarities between their model and Miconi's model.

Minor comments:

11. On ll. 440-441: The authors stated, "Our model for the experimental data of (Xiao et al., 2025) predicts that outputs of LIP neurons that learn rank estimates are routed to the hippocampal formation, and have a similar impact on grid cell firing as velocity vectors in the case of locomotion." Why do the rank estimates by LIP need to be routed back to the hippocampus? How could the present results be related to Grid cells for spatial navigation? I ask the authors to explain these points, which are unclear to me.

12. The proposed ability of single cells to learn order information is interesting. The manuscript would be stronger if the authors could propose experiments on single cells to validate the Rank Learning Rule.

(Remarks on code availability)

Reviewer #3

(Remarks to the Author)
I co-reviewed this manuscript with one of the reviewers who provided the listed reports. This is part of the Nature Communications initiative to facilitate training in peer review and to provide appropriate recognition for Early Career Researchers who co-review manuscripts.

(Remarks on code availability)

Reviewer #4

(Remarks to the Author)
The submitted paper addresses the problem of learning arbitrary orderings and abstract reasoning, and shows how the ordering relationship can be learned by single linear neurons. The paper was well written, and theoretically elegant, showing the ability to capture a complex skill by exploiting high-dimensional embeddings of data. The paper identifies six major contributions, by my reading:

1. A new loss function for rank learning and the associated learning rule.
2. A proof of convergence for the rank learning algorithm.
3. An algorithm that is implementable in neuromorphic hardware.
4. The emergence of cognitive maps from multiple learned orderings.
5. Extension of the rank-learning algorithms to contextual learning.
6. An explanation for the differences between the low-dimensional embeddings of models of biological data (linear embeddings) and the low-dimensional embeddings of data (parabolic or horse-shoe shaped embeddings).

My comments are organzied around these contributions.

1. A new loss function for rank learning and the associated learning rule.

The loss function proposed in equation 4 is a clever method of inducing an ordering for arbitrary objects. However, there is prior work on learning rankings in linear predictors. Specifically, this learning rule should be discussed in the context of the Kendall Tau loss function, and the associated hinge loss function. Section 17.4 of Shalev-Shwartz & Ben-David's "Understanding Machine Learning" (2014) would be a good reference point. That algorithm technically has access to the ranks of individual elements, in the form of y_j and y_i in the Shalev-Shwartz & Ben-David notation, but it is only ever used in the sign function, and, I think, could be replaced with the correct ordering that is used in the proposed algorithm.

A notable difference here is the parameter \delta (instead of the default 1 used in Kendall Tau). I suspect that there is likely to be interaction between the dimensionality and sparsity of the vector embeddings and the values of \delta where this learning rule will be successful. It would be interesting to see a sensitivity analysis exploring these parameters.

It is also mentioned that the learning algorithm is only ever trained on consecutive pairs of objects in the ordering. I would be interested to see the learning curve if random pairs are presented, and how that effects the estimated ranks in Figure 2.E.

2. A proof of convergence for the rank learning algorithm.

The authors provide a proof of convergence of the proposed learning rule. I found the proof itself to be somewhat challenging to follow. WRT the proof itself, I had the specific comments/questions:

- Line 508: Explain why only finitely many updates are made for a_k < a_min.
- Line 514: Does a_k having the property that w_t a_k -> -\infty contradict observation 1?
- Line 515: If a_q is the next larger item (specify larger than which, l or k? Assumed l)
- lines 516-518. a_r should be a_l? Are there some errors in the subscripts?
- Shouldn't \delta show up in this proof?

More generally, I wasn't certain why the authors couldn't just appeal to the Perceptron convergence theorem. Given the statements on lines 213-215, it seems like a perceptron with a fixed threshold of \delta, trained on samples (a_j - a_i) and correct labellings of the ordering, should converge to a correct solution. The learning rule itself operates in a delta-rule like fashion, which implies a perceptron-like structure, whether that is accounted for in the neuron model itself or the loss function. I don't think it diminishes the work in this paper to rely on the perceptron convergence theorem. Conversely, if it is known that the perceptron convergence theory can't satisfy the learning rule, then it is worth demonstrating that.

I don't mean to suggest that this learning rule should be implemented with perceptrons. That could limit the ability to use the output of the neurons as rank values, as in setion 2.5, or to construct cognitive maps, in section 2.3. It could also have similar negative consequences when implementing the algorithm in neuromorphic hardware.

3. An algorithm that is implementable in neuromorphic hardware.

The authors assert that this learning rule can be implemented in neuromorphic hardware. On the one hand, it is fairly uncontroversial that the neuromorphic hardware that supports on-chip learning can implement delta-rule style learning algorithms, and the proposed learning algorithm is compatible. However, there are a lot of implicit assumptions that relate to the structure around the network that is performing the learning. Specifically, how the inputs and the teaching signal reach the network are not specified, and neither are the structures required to implement the learning rule itself.

If the inputs were to be presented sequentially, then there is a question of how the learning rule will have access to the first pattern that is presented to the neuron, and how to ensure that the teaching signal is delivered at the appropriate time to ensure the correct relationship is learned. As presented, in the sequential form, there is an implicit assumption of random access to memory representing both a_i and a_j, which can't be guaranteed in neuromorphic hardware. Likewise, making ranking decisions with this system would require a memory to keep track of the ranks associated with the different objects presented over time.

Depending on the choice of chip, the learning rule may be limited by the neuron model itself. If that is the case, one might have to use a neuron model with a Heaviside activation function to induce the learning described by the proposed rule. This would be true for either the sequential or simultaneous presentation of the input pairs. If this is the case, one might have to employ shared weights to make sure that a linear model can be used in the applications where rank values are required.

To make the argument that this model is compatible with neuromorphic hardware, it would be necessary to see it tested in a simulated neuromorphic environment. The available learning rules will depend on hardware, so a target platform will need to be picked. The discussion of this point is fairly cursory, and warrants more detailed discussion and at least a simulated demonstration, as neuromorphic hardware can be hard to come by. Successfully demonstrated, this will be a powerful result.

4. The emergence of cognitive maps from multiple learned orderings.

I quite enjoyed this discussion, and I would like to see it expanded. I think this represents a promising discussion of how cognitive maps in arbitrary spaces could be constructed. This has important implications for cognitive neuroscience as has potentially significant applications in autonomous systems.

That said, the discussion of grid cells in section 4.3.2 seemed fairly divorced from the rest of the text, and it's only really discussed in the caption of Figure 3. Additionally, it seems like the rank learning cells in the simulated LIP project the objects into a 2D space, which are then fed into a grid-cell structure, which then has PCA performed on it to recover the 2D structure. I don't know that we should be terribly surprised that the embedding was recovered? At the very least, it would be good to compare that plot to the 2D coordinates from the learned ranks.

Lines 295-296 state "But the geometry of this cognitive map also supports completely different inference tasks, such as finding the person that has the largest mismatch between friendliness and reachability.". I think this is true, but it would also be true of other cognitive maps, or at least it isn't obvious how this is uniquely true for the proposed method.

5. Extension of the rank-learning algorithms to contextual learning.

As with the spatial relationships, this provides an interesting result. The discussion of context selecting sub-members of the population in section 4.3.1 addressed a question I had around lines 322-331, it would be good to forward reference to it to indicate how the contexts are kept separate.

That said, reading this section, I had a number of questions occurr to me:

- How much does w and w_c change during phase 2?
- Related: What are the range of values learned by w for the distinct data sets, and what are the learned values for the distinct contexts? i.e., is phase 2 just shifting context A to be offset by a large negative value or vice versa? The change in colorbar range in Figures 4H and 4I would suggest so, and this is discussed later in the paper, but it warrants mentioning here.
- How would this extend to 3 sets?
- How would/could you deal with non-disjoint sets in different contexts?

And, importantly:
- What is the sensitivity to the context learning rate?
- What does learning look like without the context?

Clearly, the much larger learning rate on the context is necessary to capture the effect of rapid learning. It would be valuable to produce a sensitivity analysis to the relative learning rates. Additionally, I would be interested in seeing how much longer it takes to learn the associations if no context was provided, and how that may compare to human learning.

6. An explanation for the differences between the low-dimensional embeddings of models of biological data.

This material provides a very elegant explanation for differences between models and biological data, I quite enjoyed this,

and I think it represents an important contribution. That said, a couple of thoughts:

A) In figure 5 the tuning curves of the soft rank selective neurons seem to have infinite support, but the derivation in section 4.5 seems to depend on neuron's response tuning curves to have finite support (e.g., equation 15). If the Gaussian curve is true, then the banded symmetric Toeplitz structure doesn't necessarily hold, and in that case, it should be stated that responses are being approximated by 0, since they are likely to be small.
B) In the main text, the motivation to approximate the covariance matrix by first the Toeplitz and then circulant matrices is pretty limited. It would improve the text if it were to say that the motivation for this approximation was to provide a tractable analysis of the low-dimensional projection that is not available directly from operating on the covariance matrix.
C) The description of how to compute a covariance matrix seems fairly straightforward, and reproducing it here (lines 629-635) is not a valuable use of space. Furthermore, as someone who frequently, albeit numerically, gets the eigevectors of covariance matrices, the reason why operating directly on the covariance matrix is not sufficient is not well motivated. What happens to the analysis in the following sections if the matrix does not have the banded symmetric Toeplitz structure? What happens if the circulant approximation is a bad approximation?

Minor Notes

- line 33 "In fact, experimental show that rank" -> experiments show?
- Figure 2 caption: Benedict and Ebbinghaus are enclosed only by closed quotation marks.
- line 181 "labeled A, ..., F" -> "labeled A through F" or "labelled A to F"?
- line 231 "But this simple arguments" -> argument
- line 254 "...proof the the Perceptron..." -> "proof of the Perceptron"?

- Figure 4: I think "Train short" and "train long" map onto phase 1 and phase 2 in the text? I got a little confused with this terminology, if possible just stick to one.
- Figure 4 F&G: the caption states it's a 2D projection of the LIP population, but the paper has been presenting learning rules for single linear neurons. Why not present this data as in Figure 2.E? Would the MDS plots be more appropriate for Figure 5?
- line 378: "...cosine function over 180 degree." -> degrees
- lines 373-386:
- I would keep the language here and in Figure 6's caption consistent (eigenvectors vs principle components)
- Projections of what onto the eigenvectors (I inferred the activities of the soft rank selective neurons' activity, but saying it would be useful to the reader)
- Make it explicit that there is a relationship between theta and the ordering of the stimuli.
- In lines 378 and 379 I would denote cosine over 180 degrees as cos(\theta), cosine over 360 degrees as cos(2\theta), which is more how it's described in Figure 6 caption, and makes it more obvious why the double angle formula is relevant.
- Figure S2. I would just increase the transparency of the shaded region and present one graph. I would also use bootstrap confidence intervals here, so you don't show better than 100% accuracy.

(Remarks on code availability)

Version 1:

Reviewer comments:

Reviewer #1

(Remarks to the Author)
The authors have done a good job addressing my comments. I'm happy to recommend acceptance.

(Remarks on code availability)

Reviewer #2

(Remarks to the Author)
The authors have addressed most of my comments and revised the manuscript adequately. Therefore, I have only a minor comment regarding my previous comment 4, which was not clear enough:

On ll. 244-256: The authors compared the convergence of their learning rule with that of the Perceptron convergence theorem. However, the form of their Rank Learning Rule more resembles the classic Competitive Learning Rule, which also converges on the surface of a high-dimensional sphere due to weight renormalization following weight update. What is a surprise if the convergence of the Rank Learning Rule is compared with that of the Competitive Learning Rule?

The authors are correct."Competitive learning rule" is a learning rule for WTA circuits. In classic PDF literature, however, WTA circuits are often not modeled explicitly. Instead, feature detection through competition is modeled by a simple learning rule for feature-selective neurons, as in chapter 5 of the following textbook: https://gwern.net/doc/ai/nn/1986-rumelhart-pdp-

v1.pdf

I observe some mathematical similarity between this and their learning rules. In their reply, the authors clarified that their Rank Learning Rule is supervised, whereas this competitive learning rule is unsupervised. It would be nice if the authors could comment on similarity and/or dissimilarity between the two rules. However, I do not insist on this point: the authors may include some comments in the manuscript if they consider such a comparison valuable.

(Remarks on code availability)

Reviewer #3

(Remarks to the Author)
I co-reviewed this manuscript with one of the reviewers who provided the listed reports. This is part of the Nature Communications initiative to facilitate training in peer review and to provide appropriate recognition for Early Career Researchers who co-review manuscripts.

(Remarks on code availability)

Reviewer #4

(Remarks to the Author)
I thank the authors for their responsiveness to my comments. I've threaded my responses (RC) to the author's responses (AR) below.

WRT "A new loss function for rank learning and the associated learning rule

RC: The loss function proposed in equation 4 ... It would be interesting to see a sensitivity analysis exploring these parameters.

AR: Thank you for pointing us to Kendall Tau loss and its upper bounded hinge loss form. The chapter in the book has a great insight why such a loss function is convex, which we believe is also applicable to our loss function. We have added a discussion after eq. (4) on ll 594. In addition, we added a parameter sensitivity analysis for δ in a new Section A.2 and Fig. S1 of the Supplement.

RC: Thank you for your response here. I concur re: convexity. I feel this matter has been completely addressed.

RC: It is also mentioned that the learning algorithm is only ever trained on consecutive pairs of objects in the ordering ... how that effects the estimated ranks in Figure 2.E.

AR: We thank the reviewer for this insightful suggestion. Following this comment, we have added additional simulations in which training pairs are sampled randomly from all possible object pairs. We now report both the corresponding learning curves in Fig. 2 D and E, and the resulting estimated ranks in Fig. 2F and Fig. S4. These new results show that training with random pairs leads to faster convergence, while the estimated ranks continue to exhibit the same characteristic S-shaped profile observed under adjacent-pair training. The latter implies that the qualitative structure of the learned rank representation is robust to the choice of training pairs.

RC: Thank you for these additional experiments. I agree with your conclusions.

AR: In the main experiments we deliberately restricted training to adjacent pairs because it provides an opportunity to probe directly the generalization capability, i.e., the transitive inference capability, of the model: we train on adjacent pairs and test it on non-adjacent pairs.

In contrast, when random pairs are used for both training and testing, the learning problem becomes easier for two reasons: (i) transitive inference is no longer required, since long-range relations are directly observed during training; and (ii) the training and test sets overlap. These issues are addressed in the new Section B.3 of the Supplement.

RC: Agreed, and I think it would be appropriate to state this more clearly around lines 168-171, in addition to your statement in the supplementary material. But I'll defer to the authors as to whether or not they think it would break the flow of the document to do so. Otherwise, I consider this matter fully addressed.

2. A proof of convergence for the rank learning algorithm

RC: Line 508: Explain why only finitely many updates are made for $a_k < a_{min}$.
AR: Done (in the proof, on ll 622).
RC: Thank you. Concern resolved.

RC: Line 514: Does a_k having the property that w_t a_k -> -\infty contradict observation 1?
AR: No, Observation 1 shows that a_k := a_min has this property.
RC: Apologies for misreading. Concern resolved.

RC: Line 515: If a_q is the next larger item (specify larger than which, l or k? Assumed l)
AR: larger than a_k is meant (added in the proof on ll 630)
RC: Thank you. Concern resolved.

RC: lines 516-518. a_r should be a_l? Are there some errors in the subscripts?
AR: The indices r and l are bound variables, that are bound by separate quantifiers, an “for all” quantifier in the preceding statement of Observation 3 and an existential quantifier in the proof on the lines to which the reviewer refers.
RC: Apologies for misreading. Concern resolved.

RC: Shouldn't \delta show up in this proof?
AR: Yes, and it does show up in the proof of Observation 3
RC: Apologies for misreading. Concern resolved.

RC: More generally, I wasn't certain ... when implementing the algorithm in neuromorphic hardware.
AR: We would like to thank the reviewer for the insightful remark on a link to the Perceptron Convergence Theorem. This does in fact provide an alternative proof of the Rank Convergence Theorem for the case where the margin δ has value 0, that we have added on ll 641.

RC: Noted, very nice description. This matter has been addressed.

AR: We would be happy to acknowledge the reviewer by name for pointing out this link

RC: I would be delighted to take the authors up on the offer, thank you. I'll reach out to the editor so they can communicate the name once the blind review process is completed.

3. An algorithm that is implementable in neuromorphic hardware.

RC: The authors assert that this learning rule can be implemented in neuromorphic hardware .. Successfully demonstrated, this will be a powerful result.

AR: We thank the reviewer for this suggestion, and the ... All computations rely on local traces and programmable synaptic plasticity that is supported by Loihi 2.

RC: Thank you for this comprehensive response. I consider this matter completely addressed.

4. The emergence of cognitive maps from multiple learned orderings

RC: I quite enjoyed this discussion ... in autonomous systems.

AR: Several models for constructing cognitive maps in abstract concept spaces have already been proposed, see e.g.

Stöckl, C., Yang, Y., & Maass, W. (2024). Local prediction-learning in high-dimensional spaces enables neural networks to plan. Nature Communications, 15(1), 2344.

Therefore we have addressed here only the case where the underlying variables are ranks of linear orders (as announced by the title of this section 2.4: „Emergence of cognitive maps from several learnt order relationships“).

RC: Point well taken. My concern is addressed.

RC: That said ... 2D coordinates from the learned ranks.

AR: We agree that the discussion of grid cells in section 4.3.2 could be better integrated with the rest of the text. To address

this, we've added a new Section C to the Supplement, which contains a comparison between the learned 2D ranks and the PCA result of the grid-cell activations for each object in Fig. S5, as suggested by the reviewer.

RC: Thank you for this additional discussion. My concerns here are addressed.

RC: Lines 295-296 state ... for the proposed method.

AR: We did not claim, or want to claim, that this inference can only be carried out via the cognitive map that emerges in this model. But we are not aware of any previous model for the emergence of a cognitive map from learning two order relations over a set of items. Only in that sense our model is “unique”. We also made an effort to keep this section 2.4 short because we wanted to focus on other material.

RC: Point well taken. I am likewise unaware of a prior demonstration. My concerns here have been resolved.

5. Extension of the rank-learning algorithms to contextual learning.

RC: As with the spatial relationships ... are kept separate.

AR: Several brain mechanisms have been elucidated that keep different contexts separate. We have added on ll 383 a reference to a recent paper to provides insight into neural mechanisms for that:

Zhang, Y., Liu, Y., Hu, Y., Zhang, Z., Du, J., Cui, L., ... & Xu, N. L. (2025). Dendritic computation for rule-based flexible categorization. bioRxiv, 2025-06.

RC: Thank you, my concerns here have been addressed.

RC: That said, reading this section ... but it warrants mentioning here.

AR: Thank you for raising this point. We have added a few sentences on ll 369 to clarify it. As shown in the new Section D.2 of the Supplementary Material ... all remaining items within each context at their correct relative rankings.

RC: Thank you for the comprehensive response. My concerns here have been addressed.

RC: How would this extend to 3 sets??

AR: The algorithm extends directly to three sets by assigning to each set a suitable context code $\mathbf{c}$. Learning proceeds in the same two-phase manner as in the two-set case. During the first phase, the model is trained only on adjacent item pairs within each context, resulting in three independently learned ranks. In the second phase, 2 boundary pairs linking the contexts are introduced, which is sufficient to merge the three sets into a single coherent ranking. The new Section D.3 and Figure S8 discuss the experimental setup and the learning process for the three-set case.

RC: Thank you for the response. My concerns here have been addressed. Additionally, apologies for the double question mark. It was a typo and not meant to imply incredulity.

RC: How would/could you deal with non-disjoint sets in different contexts

AR: An example of non-disjoint sets is already discussed in section 2.4: There the two sets on which different orders are defined in different context are in fact the same. When sets in different contexts are non-disjoint, our algorithm requires different contexts gating for different sub-groups of rank estimator neurons (as shown in Figure. 4).

RC: Apologies for misreading. My concerns were already addressed.

RC: Clearly, the much larger learning rate on the context is necessary to capture the effect of rapid learning. It would be valuable to produce a sensitivity analysis to the relative learning rates.

AR: We thank the reviewer for this suggestion and have added a sensitivity analysis of the relative learning rates in a new Figure S7. Specifically, we systematically varied the ratio η_c / η and quantified learning success across repeated simulations. Success was defined as correctly linking the two ranks across contexts during the second phase using only the boundary pair, while preserving the correct ordering within each context and across contexts.

For each ratio, the success rate was averaged over 100 independent runs, each trained from scratch with different random seeds in both phase 1 (train short) and phase 2 (train long). We find that learning stability increases monotonically with the ratio η_c/η: the success rate rises rapidly as the ratio increases, approaches unity around $\eta_c/\eta \approx 10$, and becomes consistently successful (success rate =1) for ratios larger than approximately 20. These results confirm that a larger learning rate for
the context pathway is critical for rapid and robust contextual integration and demonstrate that the effect is stable over a broad range of parameter values rather than relying on fine tuning.

RC: Thank you for the response to my concern here. I concur re: the robustness of the proposed model.

RC: Additionally, I would be interested in seeing how much longer it takes to learn the associations if no context was provided, and how that may compare to human learning.

AR: As far as we understand, it is impossible to learn several orders over the same items if no context is provided. Without context, the given information about the relative orders of individual pairs is highly contradictory.

RC: I don't know that that is strictly true, if one provided a ranking for the upper element from one set to the lower element of the other set. But this would basically be forcing the model to re-learn a global ranking, and it would likely follow the same process as learning from scratch, albeit with some inertia to over-come from the pre-training. That said, I think pursuing this matter would not add to the paper in any meaningful way. So I take the author's point, and consider my concerns resolved.

6. An explanation for the differences between the low-dimensional embeddings of models of biological data.

RC: 6. An explanation for the differences between the low-dimensional embeddings of models of biological data.

AR: We have shown in Section 2.6 that our model, as well as theory, provide the same parabolic shape of ... characterized in the review (Summerfield et al., 2020).

RC: Apologies. This was meant to be a section heading and not a comment that required a direct response. I concur with the statements the authors made in response.

RC: A) In figure 5 the tuning curves ... are likely to be small.

AR: Good point, we have added on ll 808 after eq. (20) a sentence to clarify this.

RC: Thank you. My concern here is addressed.

RC: B) In the main text, the motivation to approximate the covariance matrix by first the Toeplitz and then circulant matrices is pretty limited. It would improve the text if it were to say that the motivation for this approximation was to provide a tractable analysis of the low-dimensional projection that is not available directly from operating on the covariance matrix.
C) The description of how to compute a covariance matrix seems fairly straightforward, and reproducing it here (lines 629-635) is not a valuable use of space. Furthermore, as someone who frequently, albeit numerically, gets the eigevectors of covariance matrices, the reason why operating directly on the covariance matrix is not sufficient is not well motivated. What happens to the analysis in the following sections if the matrix does not have the banded symmetric Toeplitz structure? What happens if the circulant approximation is a bad approximation?

AR: We thank the reviewer for this suggestion and have compressed the section accordingly. The Toeplitz assumption captures the correlation structure induced by overlapping rank-selective tuning that leads to low-dimensional curvature, while the circulant approximation removes boundary effects and allows a closed-form characterization of the eigenvectors. When the covariance matrix deviates from
a banded symmetric Toeplitz form, eigenvectors must be computed numerically, but the low-
dimensional ordered geometry persists with smooth distortions. When the circulant approximation is poor, boundary effects prevent exact cosine-form eigenvectors, yet the projected representations remain low-dimensional and curved. Clarifying sentences were added after Eqs. (26), (29), and (30), and the explicit description of covariance computation was removed.

RC: Thank you, the clarifying sentences were most beneficial. My concerns here have been addressed.

Minor notes

line 660: "(with arbitrary..." -> an open parenthesis that doesn't seem to have a matching close parenthesis.
line 1057-1057: Maybe cite section 17.2 of Schalev-Schwartz & Ben-David for the interested reader?

(Remarks on code availability)

Version 2:

Reviewer comments:

Reviewer #4

(Remarks to the Author)
I am satisfied with the authors' responses and believe the paper is ready for publication.

(Remarks on code availability)
All of the code runs. There seems to be an off-by-one error in naming the files:
- The figure 3 notebook produces the hex grid pattern in figure 4.A
- the figure 4 notebook produces panels 5.D-5.I
- the Figure 5 notebook produces panels 6.B & 6.D

The code for the long-training that generates panel 5.G seems to be rotated by 90 degrees compared to the document. There also appears to be a little more overlap in the notebook than the figure in the paper. The figure for panel 5.I seems to have a little more structure than in the main document. See attached screenshot.

Open Access This Peer Review File is licensed under a Creative Commons Attribution 4.0 International License, which permits use, sharing, adaptation, distribution and reproduction in any medium or format, as long as you give appropriate credit to the original author(s) and the source, provide a link to the Creative Commons license, and indicate if changes were made.
In cases where reviewers are anonymous, credit should be given to 'Anonymous Referee' and the source.
The images or other third party material in this Peer Review File are included in the article's Creative Commons license, unless indicated otherwise in a credit line to the material. If material is not included in the article's Creative Commons license and your intended use is not permitted by statutory regulation or exceeds the permitted use, you will need to obtain permission directly from the copyright holder.
To view a copy of this license, visit https://creativecommons.org/licenses/by/4.0/

3.4. 2026

Rebuttal to the reviews of our submission:

The Inherent Capacity of Neurons to Learn Order Relations and Support Abstract Reasoning: A Theoretical Foundation for Key Features of Intelligence

REVIEWER COMMENTS

We would like to thank the reviewers for their careful reading and constructive comments. We have addressed all of them, as detailed below.

Reviewer #1 (Remarks to the Author):

This paper presents a simple, biologically plausible model of rank order learning in a neural system. The basic idea is to linearly project high-dimensional codes to an approximately one-dimensional code that respects rank-order relations. This can explain some forms of transitive inference, cognitive map formation, and curvature properties of neural representations. Importantly, the authors present rigorous mathematical proofs of convergence.

Overall, I thought this was an interesting and well-written paper. It was hard for me to tell exactly how ground-breaking it is because there's not really a head-to-head comparison with alternative models in terms of explanations and predictions (see below). I think the learning theory is valuable, an aspect that goes beyond previous work in this area.

Comments:

The biological plausibility of the context learning rule is unclear to me.

This is a good point!

We added on ll 377 the following explanation:

The context learning rule is basically the same as the Rank Learning Rule: It adds a fraction of the synaptic inputs for the item whose rank is too low to the current synaptic weights, and correspondingly applies LTD if the rank is too high. But it employs a separate learning rate for those components of the synaptic inputs that carry information about the context. This separate learning rate is biologically plausible because context information from higher brain areas typically arrives in L1 of a cortical column, through synapses on distal apical dendrites of pyramidal cells, see e.g. the experimental data of (Zhang et al., 2025). Hence it arrives in a different dendritic compartment than bottom-up sensory inputs. It is commonly assumed that different compartments of a biological neuron are subject to different plasticity processes (see e.g. (Wright et al., 2025)). Hence, the assumption that plasticity of synapses that carry context information is subject to a separate learning rate appears to be biologically plausible.

We have added in addition on ll 229 references to the literature which support the biological plausibility of the Rank Learning Rule in general.

The authors make a good case for some of the computational advantages of their approach relative to that of Nelli et al. (2023). I'm wondering whether these models also make different empirical predictions?

Thank you for raising this important question! In response we have added on ll 519 the following remarks (that also integrate some text from the original submission):

The primary empirically testable prediction of our model is that learnt ranks of an order are represented by firing rates of neurons, integrated into the dominant 1D dynamics of area LIP (which had been elucidated by \citep{ganguli2008one}). In other words, our model predicts that these ranks are represented in the brain in a similar fashion as numbers and other types of magnitudes, whose neural representation had been elucidated by \citep{nieder2009representation, roitman2012representation, nieder2016neuronal, lyons2016ordinality, lorenzi2021numerosities, luyckx2019neural, summerfield2020structure, bottini2020knowledge, vigano2024spontaneous, xu2024inferior}. The data of \citep{munoz2020learned} suggest that this prediction is likely to become true. They used an order learning task that was very similar to the one we used, but with the difference that two items of the order were always presented simultaneously. They found that for this type of presentation the sum of ranks of the two simultaneously presented items was represented by the firing rate of LIP neurons. Our model predicts that also for a presentation of a single item its learnt rank is likely to be represented by the firing rate of a neuron.

The experimental data of \citep{Xiao2025human} suggest that explicit representations of this latent scalar variable also emerge in the human brain, since the formation of a 2D cognitive map for items that are characterized by values of 2 scalar variables was so far only observed when explicit representations of the underlying two scalar variables were formed in the brain. But further experiments are needed to verify this for the case of rank representations in the human brain.

The models of \citep{nelli2023neural, lippl2024mathematical, miconi2025neural} did not predict that explicit representations of learnt ranks of items are formed in the brain, since these models consisted of neural networks with hidden units that were trained to solve a decision problem that does not require explicit representations of learnt ranks.

In addition, these preceding models predicted that complex top-down learning signals can be found, since these as needed for an implementation of BPTT in a neural network. In contrast, our model predicts that only the ground truth about the order between two presented items, that is explicitly given to the subject, is transmitted as top-down learning signal, and a local synaptic plasticity process suffices.

Same question for the modeling of Lippl et al. (2024).

We have added in response to this question on ll 502 the following sentences:

The work \citep{lippl2024mathematical} had previously studied general properties of neural networks that are trained to solve the decision problem “does X come after Y in the order”,

especially with regard to their resulting transitive inference capability. They focus on neural networks with non-additive representations, whereas our model uses an additive representation according to their terminology. In fact, our model seems to provide the simplest possible model for solving this decision task (by comparing the learnt ranks of X and Y), since it consists of just a single linear neuron. Interestingly, they had shown that any model with additive representation necessarily implements a ranking system.

The model doesn't directly predict the symbolic distance effect because it lacks a mapping to response time, and lacks a representation of noise that presumably is needed to explain the effect. I don't think it's necessary to show this in a revision, but the authors could clarify what is needed to show this.

We are grateful for this remark, and have added on ll 179 the following remark:

In order to demonstrate this rigorously one has to transform the rank estimates of our model into rates of Poisson spike trains, and analyze the time it takes to compare the firing rates for two adjacent ranks with that for two items with a larger rank difference.

At the end of the Discussion, the authors suggest that this form of learning rule is relevant for energy-efficient devices. Because they have not demonstrated energy efficiency, this claim is not well validated.

Thank you for this important comment. We agree that our original statement was insufficiently supported. In the revised manuscript, we address this concern by implementing our model on Intel Loihi 2, a neuromorphic processor designed for sparse event driven computation, and local learning, all of which are closely linked to energy efficient execution. Intel's technical documentation describe Loihi 2 as an energy efficient neuromorphic platform, and report substantial efficiency gains for appropriate workloads. We therefore revised the Discussion to ground our claim in this actual hardware implementation, rather than leaving it as a speculative extrapolation.

We are discussing the implementation on Loihi 2 in a new section 2.3 and a new Fig. 3 in Results section, together with a new section 4.3 in Methods section.

Typos:
"experimental show" -> "experiments show"
"this simple arguments" -> "this simple argument"
"a 2D cognitive maps" -> "a 2D cognitive map"
"configuration of internal" -> "configuration of internal codes"

Thank you for your careful reading, we have fixed these errors.

Reviewer #2 (Remarks to the Author):

This work computationally studied the neural mechanisms by which the brain learns order relationships (like ranks) of multiple objects and uses them for reasoning, typically transitive inference. The authors proposed a simple Rank Learning Rule, which enables single neurons in a

feedforward network to adjust their afferent connections when they make an error in predicting the order of paired items. The authors proved mathematically that this rule always converges to a correct solution within finite learning steps when the neurons are informed of the order relationships between some pairs sampled from multiple items. Their model explains well-known effects in human and animal reasoning, such as the symbolic distance effect (easier comparisons for items far apart) and the terminal item effect (better performance for items at the ends of a sequence). The model also explains why neural representations for rank order projected onto the two-dimensional space spanned by the leading principal components often look U-shaped rather than straight in experiments.

Overall, the work suggests an interesting possibility that neurons have an inherent ability to learn and represent order relationships, supporting abstract reasoning without requiring complex deep learning. The authors also claimed that their findings have implications both for neuroscience and for building more efficient neuromorphic hardware. However, this reviewer considers that several points should be clarified before the manuscript is accepted.

Major comments:

1. At ll. 89-93: In the introduction, the authors stated, "We then address experimental data which show how the brain integrates information from different learnt order relations into a cognitive map (Xiao et al., 2025), or into a larger order (Nelli et al., 2023). We are not aware of any model for the former, and only of a multi-layer perceptron model trained through stochastic gradient descent with different learning rates for each pair for the latter." If my understanding is correct, Miconi and Kay (Nat Neurosci, 2025) proposed another neural mechanism that can integrate shorter relational representations into a larger representation. The above statements need revision.

We agree, and we have added a reference to (Miconi et al., 2025) to this sentence on ll 93.

2. On page 4: The output firing rate of each LIP neuron, r(a_i), should be defined explicitly near Equation 1. Is r(a_i) given as the inner product w*a_i?

Thanks for catching this. We have added a clear definition on ll 142.

3. Fig. S1: The results shown in the figure seem to compare simulation results and experimental data. These data should be clearly indicated in the figure itself or the legend.

Thanks.

Former Fig. S1 is now Fig S2. We have stated there now in the caption that the range that is defined by the red arrows is from experimental data, and we have cited the sources. All data points are from our model.

4. On ll. 244-256: The authors compared the convergence of thueir learning rule with that of the Perceptron convergence theorem. However, the form of their Rank Learning Rule more resembles the classic Competitive Learning Rule, which also converges on the surface of a high-dimensional sphere due to weight renormalization following weight update. What is a surprise if the convergence of the Rank Learning Rule is compared with that of the Competitive Learning Rule?

To the best of our knowledge (and that of Wikipedia and Google AI) the “competitive learning rule” is a learning rule for WTA circuits, like the one that we had analyzed in

Nessler, B., Pfeiffer, M., Buesing, L., & Maass, W. (2013). Bayesian computation emerges in generic cortical microcircuits through spike-timing-dependent plasticity. PLoS computational biology, 9(4), e1003037.

But we do not manage to find similarities between these competitive learning rules and the rank order learning rule for a single neuron. One fundamental difference is that competitive learning rules are rules for a neural circuit with lateral inhibition, rather than for a single neuron. Also on the abstract mathematical level these rules are very different, since competitive learning rules do not require any teacher input, whereas the rank learning rule requires that.

Finally, we are not aware of theoretical convergence results for competitive learning rules.

5. On ll. 299-303: Phases 1 and 2 of learning are called “train short” and “train long” in the next paragraph. However, this writing is confusing as these names are not defined before their use.

Thank you. These two terms are directly borrowed from (Nelli et al., 2023). We have added an explanation in the Figure caption where these two terms are firstly introduced (this is now Fig. 5).

6. On ll. 318-322: It was difficult for me to understand the definition of Contextual Learning Rule given in Equation 3. Please explain it more clearly.

Thank you. We have added on ll 369 after eq. (3) some sentences to enhance both the mathematical clarity and the intuitive understanding:

The context learning rule is basically the same as the rank learning rule: It adds a fraction of the synaptic inputs for the item whose rank is too low to the current synaptic weights, and correspondingly applies LTD if the rank is too high. But it employs a separate learning rate for those components of the synaptic inputs that carry information about the context. A separate learning rate for these synaptic inputs is biologically plausible because context information from higher brain areas typically arrives in L1 of a cortical column, through synapses on distal apical dendrites of pyramidal cells, see the data of \citep{zhang2025dendritic}. Thus context information arrives in a different dendritic compartment than bottom-up sensory inputs, that typically arrive closer to the soma. It is commonly assumed that different compartments of a biological neuron are subject to different plasticity processes (see e.g. \citep{wright2025distinct}. These are likely to have also different learning rates.

7. On ll. 323-324: The authors used an 8 times larger rate for learning the context weights compared to learning rank weights. Is this fast learning of context essential for the model's performance? What happens if the two learning rates are comparable? Please explain these points in more detail.

This is a good question! We have added a new section D2 and a new Fig. S6 to the Supplement that provide an answer. Figure S6 shows results for 4 different ratios, (η_c/η), between the context learning rate η_c and the in-context rank learning rate η. When this ratio is reduced (panels from left to right), the in-context order tends to become distorted, since the model

prioritizes the satisfaction of individual boundary training pairs, rather than jointly shifting all objects within the same context.

8. On ll. 330-331: The authors mentioned "One sees in Fig. 4E that in general 2 presentations of the boundary pair suffice for adjusting all rank estimates for the long order." The reason was not immediately clear to me. Please explain why learning the boundary pair is sufficient in the proposed model.

We have added on ll 371 the following explanation:

Since the context information $\mathbf{c}$ is shared by all objects within a context, adjustments to $\mathbf{w}_c$ shift the rank estimates of all objects together. When the ratio between η_c and η is sufficiently high, learning on boundary pairs is prioritized by shifting each group as a whole, rather than adjusting only the boundary pair.

9. Fig. 5: To account for the U-shaped 2-dimensional representations, the authors assumed an array of readout neurons with staggered firing thresholds. Theoretically, the hypothesis sounds interesting. However, considering the highly noisy features of cortical circuits, decisions by such an array of neurons may not be robust enough, especially when the number of objects becomes large. Is there any evidence for neurons with staggered thresholds?

We have added the following explanation and references on ll 418:

In order to model the emergence of a heterogeneous code for ranks it suffices to route the output of neurons that encode ranks in a summation code to a population of excitatory neurons with staggered firing thresholds, see Fig. \ref{fig:figure6}A. It is well known that different biological neurons tend to have different excitability , see e.g. \citep{ marder2006variability, mozzachiodi2010more, buzsaki2014log}. The excitability of a biological neuron corresponds to the firing threshold of a simple neuron model. But also other factors, such as synaptic gain and inhibitory feedback regulate the firing response of a biological neuron.

Recordings from neural circuits of the brain have shown that scalar variables are not only encoded by a "summation code", but also by a "heterogeneous code" \citep{summerfield2020structure}, where each neuron responds only to values of the scalar variable from some bounded interval; see our ll 410

How does the U-shape of the projected representations behave in the model when the number of objects is increased? Are the errors generated by the model consistent with those observed in experiments?

These are very good questions!

To address them, we have added a new section E.2 with three news Figures S10–S12 to the Supplement. First, Fig. S10 examines how the projected representations evolve as the number of objects increases. We find that the characteristic U-shaped geometry is preserved when the number of objects grows, and a gradual finer-graded sampling, indicating that the model scales smoothly to

larger numbers of objects. Second, Fig. S11 evaluates the robustness of the model under increasing noise levels. Despite substantial perturbations, the ordering and low-dimensional structure of the representations remain stable, consistent with the behavior of population codes in noisy cortical circuits. Finally, Fig. S12 shows how the geometry depends on the number of soft rank-selective neurons contributing to the encoding of the same rank. Varying the number of these neurons changes the shape of the code but does not eliminate the U-shaped structure, demonstrating that the effect does not rely on a finely tuned or minimal number of neurons.}

We have added a pointer to these supplementary figures on ll 436 of the Results section.

10. On ll. 332-340: The authors compared their model with a deep network by Neilli et al. (2023), which can also combine separately learned orders into a long representation. In a recent paper, Miconi and Kay (Nat Neurosci, 2025) proposed another mechanism to integrate shorter relational representations into a larger one. The paper is cited in the manuscript, but the authors are asked to argue the differences and similarities between their model and Miconi's model.

We have added a discussion of differences and similarities with the model of (Miconi et al., 2025) on ll 489 of the Discussion, and provide further comparison details on ll 886 of Methods.

Minor comments:

11. On ll. 440-441: The authors stated, “Our model for the experimental data of (Xiao et al., 2025) predicts that outputs of LIP neurons that learn rank estimates are routed to the hippocampal formation, and have a similar impact on grid cell firing as velocity vectors in the case of locomotion.” Why do the rank estimates by LIP need to be routed back to the hippocampus? How could the present results be related to Grid cells for spatial navigation? I ask the authors to explain these points, which are unclear to me.

This is a good point!

We have expanded this sentence as follows in order to provide a better explanation (it is now on ll 533):

The experimental data of \citep{xiao2025human} suggest that explicit representations of this latent scalar variable also emerge in the human brain, since the formation of a 2D cognitive map for items that are characterized by values of 2 scalar variables was so far only observed when explicit representations of the underlying two scalar variables were formed in the brain. But further experiments are needed to verify this for the case of rank representations in the human brain. Our model also predicts that outputs of LIP neurons that encode learnt rank estimates are routed to the entorhinal cortex/, and play there a similar role for advancing grid cell firing as efferent copies of motor commands in the case of locomotion.

12. The proposed ability of single cells to learn order information is interesting. The manuscript would be stronger if the authors could propose experiments on single cells to validate the Rank Learning Rule.

This is a very good suggestion! We have added on ll 520:

The primary empirically testable prediction of our model is that learnt ranks of an order are represented by firing rates of neurons, integrated into the dominant 1D dynamics of area LIP (which had been elucidated by \citep{ganguli2008one}). In other words, our model predicts that these ranks are represented in the brain in a similar fashion as numbers and other types of magnitudes, whose neural representation had been elucidated by \citep{nieder2009representation, roitman2012representation, nieder2016neuronal, lyons2016ordinality, lorenzi2021numerosities, luyckx2019neural, summerfield2020structure, bottini2020knowledge, vigano2024spontaneous, xu2024inferior}. The data of \citep{munoz2020learned} suggest that this prediction has a good chance to be verified. They used an order learning task that was very similar to the one we used, but with the difference that two items of the order were always presented simultaneously. They found that for this type of presentation the sum of ranks of the two simultaneously presented items was represented by the firing rate of LIP neurons. Our model predicts that also for a presentation of a single item its learnt rank is likely to be represented by the firing rate of a neuron.

Reviewer #3 (Remarks to the Author):

I co-reviewed this manuscript with one of the reviewers who provided the listed reports. This is part of the Nature Communications initiative to facilitate training in peer review and to provide appropriate recognition for Early Career Researchers who co-review manuscripts.

Reviewer #4 (Remarks to the Author):

The submitted paper addresses the problem of learning arbitrary orderings and abstract reasoning, and shows how the ordering relationship can be learned by single linear neurons. The paper was well written, and theoretically elegant, showing the ability to capture a complex skill by exploiting high-dimensional embeddings of data. The paper identifies six major contributions, by my reading:

1. A new loss function for rank learning and the associated learning rule.
2. A proof of convergence for the rank learning algorithm.
3. An algorithm that is implementable in neuromorphic hardware.
4. The emergence of cognitive maps from multiple learned orderings.
5. Extension of the rank-learning algorithms to contextual learning.
6. An explanation for the differences between the low-dimensional embeddings of models of biological data (linear embeddings) and the low-dimensional embeddings of data (parabolic or horse-shoe shaped embeddings).

My comments are organzied around these contributions.

1. A new loss function for rank learning and the associated learning rule.

The loss function proposed in equation 4 is a clever method of inducing an ordering for arbitrary objects. However, there is prior work on learning rankings in linear predictors. Specifically, this learning rule should be discussed in the context of the Kendall Tau loss function, and the associated hinge loss function. Section 17.4 of Shalev-Shwartz & Ben-David's "Understanding Machine Learning" (2014) would be a good reference point. That algorithm technically has access to the ranks of individual elements, in the form of y_j and y_i in the Shalev-Shwartz & Ben-David notation, but it is only ever used in the sign function, and, I think, could be replaced with the correct ordering that is used in the proposed algorithm.

A notable difference here is the parameter \delta (instead of the default 1 used in Kendall Tau). I suspect that there is likely to be interaction between the dimensionality and sparsity of the vector embeddings and the values of \delta where this learning rule will be successful. It would be interesting to see a sensitivity analysis exploring these parameters.

Thank you for pointing us to Kendall Tau loss and its upper bounded hinge loss form. The chapter in the book has a great insight why such a loss function is convex, which we believe is also applicable to our loss function. We have added a discussion after eq. (4) on ll 594. In addition, we added a parameter sensitivity analysis for δ in a new Section A.2 and Fig. S1 of the Supplement.

It is also mentioned that the learning algorithm is only ever trained on consecutive pairs of objects in the ordering. I would be interested to see the learning curve if random pairs are presented, and how that effects the estimated ranks in Figure 2.E.

We thank the reviewer for this insightful suggestion. Following this comment, we have added additional simulations in which training pairs are sampled randomly from all possible object pairs. We now report both the corresponding learning curves in Fig. 2 D and E, and the resulting estimated ranks in Fig. 2F and Fig. S4. These new results show that training with random pairs leads to faster convergence, while the estimated ranks continue to exhibit the same characteristic S-shaped profile observed under adjacent-pair training. The latter implies that the qualitative structure of the learned rank representation is robust to the choice of training pairs.

In the main experiments we deliberately restricted training to adjacent pairs because it provides an opportunity to probe directly the generalization capability, i.e., the transitive inference capability, of the model: we train on adjacent pairs and test it on non-adjacent pairs.

In contrast, when random pairs are used for both training and testing, the learning problem becomes easier for two reasons: (i) transitive inference is no longer required, since long-range relations are directly observed during training; and (ii) the training and test sets overlap. These issues are addressed in the new Section B.3 of the Supplement.

2. A proof of convergence for the rank learning algorithm.

The authors provide a proof of convergence of the proposed learning rule. I found the proof itself to

be somewhat challenging to follow. WRT the proof itself, I had the specific comments/questions:

- Line 508: Explain why only finitely many updates are made for a_k < a_min.

Done (in the proof, on ll 622).

- Line 514: Does a_k having the property that w_t a_k -> -\infty contradict observation 1?

No, Observation 1 shows that a_k := a_min has this property.

- Line 515: If a_q is the next larger item (specify larger than which, l or k? Assumed l)

larger than a_k is meant (added in the proof on ll 630)

- lines 516-518. a_r should be a_l? Are there some errors in the subscripts?

The indices r and l are bound variables, that are bound by separate quantifiers, an "for all" quantifier in the preceding statement of Observation 3 and an existential quantifier in the proof on the lines to which the reviewer refers.

- Shouldn't \delta show up in this proof?

Yes, and it does show up in the proof of Observation 3

More generally, I wasn't certain why the authors couldn't just appeal to the Perceptron convergence theorem. Given the statements on lines 213-215, it seems like a perceptron with a fixed threshold of \delta, trained on samples (a_j - a_i) and correct labellings of the ordering, should converge to a correct solution. The learning rule itself operates in a delta-rule like fashion, which implies a perceptron-like structure, whether that is accounted for in the neuron model itself or the loss function. I don't think it diminishes the work in this paper to rely on the perceptron convergence theorem. Conversely, if it is known that the perceptron convergence theory can't satisfy the learning rule, then it is worth demonstrating that.

I don't mean to suggest that this learning rule should be implemented with perceptrons. That could limit the ability to use the output of the neurons as rank values, as in setion 2.5, or to construct cognitive maps, in section 2.3. It could also have similar negative consequences when implementing the algorithm in neuromorphic hardware.

We would like to thank the reviewer for the insightful remark on a link to the Perceptron Convergence Theorem. This does in fact provide an alternative proof of the Rank Convergence Theorem for the case where the margin δ has value 0, that we have added on ll 641.

We would be happy to acknowledge the reviewer by name for pointing out this link.

3. An algorithm that is implementable in neuromorphic hardware.

The authors assert that this learning rule can be implemented in neuromorphic hardware. On the one hand, it is fairly uncontroversial that the neuromorphic hardware that supports on-chip learning can implement delta-rule style learning algorithms, and the proposed learning algorithm is compatible. However, there are a lot of implicit assumptions that relate to the structure around the network that is performing the learning. Specifically, how the inputs and the teaching signal reach the network are not specified, and neither are the structures required to implement the learning rule itself.

If the inputs were to be presented sequentially, then there is a question of how the learning rule will have access to the first pattern that is presented to the neuron, and how to ensure that the teaching signal is delivered at the appropriate time to ensure the correct relationship is learned. As presented, in the sequential form, there is an implicit assumption of random access to memory representing both a_i and a_j, which can't be guaranteed in neuromorphic hardware. Likewise, making ranking decisions with this system would require a memory to keep track of the ranks associated with the different objects presented over time.

Depending on the choice of chip, the learning rule may be limited by the neuron model itself. If that is the case, one might have to use a neuron model with a Heaviside activation function to induce the learning described by the proposed rule. This would be true for either the sequential or simultaneous presentation of the input pairs. If this is the case, one might have to employ shared weights to make sure that a linear model can be used in the applications where rank values are required.

To make the argument that this model is compatible with neuromorphic hardware, it would be necessary to see it tested in a simulated neuromorphic environment. The available learning rules will depend on hardware, so a target platform will need to be picked. The discussion of this point is fairly cursory, and warrants more detailed discussion and at least a simulated demonstration, as neuromorphic hardware can be hard to come by. Successfully demonstrated, this will be a powerful result.
We thank the reviewer for this suggestion, and the careful analysis of the architectural assumptions underlying our learning rule. In the revised manuscript, we now provide in a new Section 2.3 both (i) an explicit hardware realization and (ii) a concrete experimental demonstration on Intel Loihi 2. The new Figure 3 details the full signal routing, timing, and local state structure required for implementation.

We chose Intel Loihi 2 as target neuromorphic platform because it is a state-of-the-art neuromorphic chip, and because it supports programmable neuron models and on-chip synaptic plasticity. The algorithm has been implemented and executed on a physical Loihi 2 hardware system (8-chip configuration). Learning occurs fully on-chip.

Figure 3A shows the hardware platform. Figure 3B–C specifies the architectural components required for implementation. Fig 3D shows the successful on-chip learning results. This replaces the previous high-level claim with a concrete demonstration.

Importantly, the neuron does not store the presynaptic patterns. During the first pass, the neuromorphic core computes only the predicted rank difference $\Delta r = r(aj) - r(ai)$ and outputs it. The error signal eij is computed off-core and injected through a dedicated input channel. Learning is

triggered by re-presenting the same input pair together with the injected error. Therefore, the model does not assume random-access retrieval of ai or aj inside the neuromorphic core.

The timing concern is addressed through explicit micro-time scheduling, shown in Figure 3C. The sequence is: present aj, present ij, read Δr, inject eij, then re-present the pair to induce the local weight update Δw = η eij (aj – ai). All computations rely on local traces and programmable synaptic plasticity that is supported by Loihi 2.

4. The emergence of cognitive maps from multiple learned orderings.

I quite enjoyed this discussion, and I would like to see it expanded. I think this represents a promising discussion of how cognitive maps in arbitrary spaces could be constructed. This has important implications for cognitive neuroscience as has potentially significant applications in autonomous systems.

Several models for constructing cognitive maps in abstract concept spaces have already been proposed, see e.g.

Stöckl, C., Yang, Y., & Maass, W. (2024). Local prediction-learning in high-dimensional spaces enables neural networks to plan. *Nature Communications*, *15*(1), 2344.

Therefore we have addressed here only the case where the underlying variables are ranks of linear orders (as announced by the title of this section 2.4: „Emergence of cognitive maps from several learnt order relationships“).

That said, the discussion of grid cells in section 4.3.2 seemed fairly divorced from the rest of the text, and it's only really discussed in the caption of Figure 3. Additionally, it seems like the rank learning cells in the simulated LIP project the objects into a 2D space, which are then fed into a grid-cell structure, which then has PCA performed on it to recover the 2D structure. I don't know that we should be terribly surprised that the embedding was recovered? At the very least, it would be good to compare that plot to the 2D coordinates from the learned ranks.

We agree that the discussion of grid cells in section 4.3.2 could be better integrated with the rest of the text. To address this, we've added a new Section C to the Supplement, which contains a comparison between the learned 2D ranks and the PCA result of the grid-cell activations for each object in Fig. S5, as suggested by the reviewer.

Lines 295-296 state "But the geometry of this cognitive map also supports completely different inference tasks, such as finding the person that has the largest mismatch between friendliness and reachability.". I think this is true, but it would also be true of other cognitive maps, or at least it isn't obvious how this is uniquely true for the proposed method.

We did not claim, or want to claim, that this inference can only be carried out via the cognitive map that emerges in this model. But we are not aware of any previous model for the emergence of a cognitive map from learning two order relations over a set of items. Only in that sense our model is “unique”. We also made an effort to keep this section 2.4 short because we wanted to focus on other material.

5. Extension of the rank-learning algorithms to contextual learning.

As with the spatial relationships, this provides an interesting result. The discussion of context selecting sub-members of the population in section 4.3.1 addressed a question I had around lines 322-331, it would be good to forward reference to it to indicate how the contexts are kept separate.

Several brain mechanisms have been elucidated that keep different contexts separate. We have added on ll 383 a reference to a recent paper to provides insight into neural mechanisms for that:

Zhang, Y., Liu, Y., Hu, Y., Zhang, Z., Du, J., Cui, L., ... & Xu, N. L. (2025). Dendritic computation for rule-based flexible categorization. bioRxiv, 2025-06.

That said, reading this section, I had a number of questions occurr to me:

- How much does w and w_c change during phase 2?
- Related: What are the range of values learned by w for the distinct data sets, and what are the learned values for the distinct contexts? i.e., is phase 2 just shifting context A to be offset by a large negative value or vice versa? The change in colorbar range in Figures 4H and 4I would suggest so, and this is discussed later in the paper, but it warrants mentioning here.

Thank you for raising this point. We have added a few sentences on ll 369 to clarify it. As shown in the new Section D.2 of the Supplementary Material, the updates of w and w_c are proportional to the ratio between $\eta||\boldsymbol{a}||_2^2$ and $\eta_c||\boldsymbol{c}||_2^2$. In all experiments, we set $||\boldsymbol{a}||_2^2 = ||\boldsymbol{c}||_2^2$, so the relative magnitude of the updates to w and w_c is determined solely by the learning rates. We use $\eta_c = 8\eta$ throughout. In Phase 2, updates to w_c shift the two contexts in opposite directions, one with a positive offset and the other with a negative offset. This separation of the boundary pairs simultaneously places all remaining items within each context at their correct relative rankings.

- How would this extend to 3 sets??

The algorithm extends directly to three sets by assigning to each set a suitable context code $\mathbf{c}$. Learning proceeds in the same two-phase manner as in the two-set case. During the first phase, the model is trained only on adjacent item pairs within each context, resulting in three independently learned ranks. In the second phase, 2 boundary pairs linking the contexts are introduced, which is sufficient to merge the three sets into a single coherent ranking. The new Section D.3 and Figure S8 discuss the experimental setup and the learning process for the three-set case.

- How would/could you deal with non-disjoint sets in different contexts

An example of non-disjoint sets is already discussed in section 2.4: There the two sets on which different orders are defined in different context are in fact the same. When sets in different contexts are non-disjoint, our algorithm requires different contexts gating for different sub-groups of rank estimator neurons (as shown in Figure. 4).

And, importantly:
- What is the sensitivity to the context learning rate?
- What does learning look like without the context?

Clearly, the much larger learning rate on the context is necessary to capture the effect of rapid learning. It would be valuable to produce a sensitivity analysis to the relative learning rates.

We thank the reviewer for this suggestion and have added a sensitivity analysis of the relative learning rates in a new Figure S7. Specifically, we systematically varied the ratio η_c/η and quantified learning success across repeated simulations. Success was defined as correctly linking the two ranks across contexts during the second phase using only the boundary pair, while preserving the correct ordering within each context and across contexts.

For each ratio, the success rate was averaged over 100 independent runs, each trained from scratch with different random seeds in both phase 1 (train short) and phase 2 (train long). We find that learning stability increases monotonically with the ratio η_c/η: the success rate rises rapidly as the ratio increases, approaches unity around $\eta_c/\eta \approx 10$, and becomes consistently successful (success rate =1) for ratios larger than approximately 20. These results confirm that a larger learning rate for the context pathway is critical for rapid and robust contextual integration and demonstrate that the effect is stable over a broad range of parameter values rather than relying on fine tuning.

Additionally, I would be interested in seeing how much longer it takes to learn the associations if no context was provided, and how that may compare to human learning.

As far as we understand, it is impossible to learn several orders over the same items if no context is provided. Without context, the given information about the relative orders of individual pairs is highly contradictory.

6. An explanation for the differences between the low-dimensional embeddings of models of biological data.

We have shown in Section 2.6 that our model, as well as theory, provide the same parabolic shape of 2D projections of neural representations of items as the experimental data from the human brain in (Nelli et al., 2023). Hence we are not sure which difference the reviewer wants to address here. The difference to the quasi-linear representations in Fig. 5F, G arises from the presence of heterogeneously coding neurons in the model of Section 2.6, as characterized in the review (Summerfield et al., 2020).

This material provides a very elegant explanation for differences between models and biological data, I quite enjoyed this, and I think it represents an important contribution. That said, a couple of thoughts:

A) In figure 5 the tuning curves of the soft rank selective neurons seem to have infinite support, but the derivation in section 4.5 seems to depend on neuron's response tuning curves to have finite

support (e.g., equation 15). If the Gaussian curve is true, then the banded symmetric Toeplitz structure doesn't necessarily hold, and in that case, it should be stated that responses are being approximated by 0, since they are likely to be small.

Good point, we have added on ll 808 after eq. (20) a sentence to clarify this.

B) In the main text, the motivation to approximate the covariance matrix by first the Toeplitz and then circulant matrices is pretty limited. It would improve the text if it were to say that the motivation for this approximation was to provide a tractable analysis of the low-dimensional projection that is not available directly from operating on the covariance matrix.
C) The description of how to compute a covariance matrix seems fairly straightforward, and reproducing it here (lines 629-635) is not a valuable use of space. Furthermore, as someone who frequently, albeit numerically, gets the eigevectors of covariance matrices, the reason why operating directly on the covariance matrix is not sufficient is not well motivated. What happens to the analysis in the following sections if the matrix does not have the banded symmetric Toeplitz structure? What happens if the circulant approximation is a bad approximation?

We thank the reviewer for this suggestion and have compressed the section accordingly. The Toeplitz assumption captures the correlation structure induced by overlapping rank-selective tuning that leads to low-dimensional curvature, while the circulant approximation removes boundary effects and allows a closed-form characterization of the eigenvectors. When the covariance matrix deviates from a banded symmetric Toeplitz form, eigenvectors must be computed numerically, but the low-dimensional ordered geometry persists with smooth distortions. When the circulant approximation is poor, boundary effects prevent exact cosine-form eigenvectors, yet the projected representations remain low-dimensional and curved. Clarifying sentences were added after Eqs. (26), (29), and (30), and the explicit description of covariance computation was removed.

Minor Notes

- line 33 "In fact, experimental show that rank" -> experiments show?
- Figure 2 caption: Benedict and Ebbinghaus are enclosed only by closed quotation marks.
- line 181 "labeled A, ..., F" -> "labeled A through F" or "labelled A to F"?
- line 231 "But this simple arguments" -> argument
- line 254 "...proof the the Perceptron..." -> "proof of the Perceptron"?

- Figure 4: I think "Train short" and "train long" map onto phase 1 and phase 2 in the text? I got a little confused with this terminology, if possible just stick to one.
- Figure 4 F&G: the caption states it's a 2D projection of the LIP population, but the paper has been presenting learning rules for single linear neurons. Why not present this data as in Figure 2.E? Would the MDS plots be more appropriate for Figure 5?
- line 378: "...cosine function over 180 degree." -> degrees
- lines 373-386:
- I would keep the language here and in Figure 6's caption consistent (eigenvectors vs principle components)
- Projections of what onto the eigenvectors (I inferred the activities of the soft rank selective

neurons' activity, but saying it would be useful to the reader)
- Make it explicit that there is a relationship between theta and the ordering of the stimuli.
- In lines 378 and 379 I would denote cosine over 180 degrees as cos(\theta), cosine over 360 degrees as cos(2\theta), which is more how it's described in Figure 6 caption, and makes it more obvious why the double angle formula is relevant.
- Figure S2. I would just increase the transparency of the shaded region and present one graph. I would also use bootstrap confidence intervals here, so you don't show better than 100% accuracy.

All done, thank you for your careful reading!

14.5.2026

Rebuttal to the 2nd review of Nature Communications manuscript NCOMMS-25-56574A by Yukun Yang and Wolfgang Maass

We would like to thank the reviewers for their helpful comments!

In this 2nd round two (optional) additions of comments were suggested by Reviewers #2 and #4, and two minor points were pointed out by Reviewer #4. We have implemented all of these suggestions, as detailed below.

REVIEWER COMMENTS

Reviewer #1 (Remarks to the Author):

The authors have done a good job addressing my comments. I'm happy to recommend acceptance.

Reviewer #2 (Remarks to the Author):

The authors have addressed most of my comments and revised the manuscript adequately. Therefore, I have only a minor comment regarding my previous comment 4, which was not clear enough:

On ll. 244-256: The authors compared the convergence of their learning rule with that of the Perceptron convergence theorem. However, the form of their Rank Learning Rule more resembles the classic Competitive Learning Rule, which also converges on the surface of a high-dimensional sphere due to weight renormalization following weight update. What is a surprise if the convergence of the Rank Learning Rule is compared with that of the Competitive Learning Rule?

The authors are correct.“Competitive learning rule" is a learning rule for WTA circuits. In classic PDF literature, however, WTA circuits are often not modeled explicitly. Instead, feature detection through competition is modeled by a simple learning rule for feature-selective neurons, as in chapter 5 of the following textbook: https://gwern.net/doc/ai/nn/1986-rumelhart-pdp-v1.pdf

I observe some mathematical similarity between this and their learning rules. In their reply, the authors clarified that their Rank Learning Rule is supervised, whereas this competitive learning rule is unsupervised. It would be nice if the authors could comment on similarity and/or dissimilarity between the two rules. However, I do not insist on this point: the authors may include some comments in the manuscript if they consider such a comparison valuable.

A: Thank you for pointing out this similarity. We have addressed it on ll 240 by adding the following explanation:

The weight dynamics under the Rank Learning Rule can intuitively be understood as follows: The weight vector moves toward the higher ranked input vector and away from the lower ranked input vector. This is somewhat analogous to the competitive learning of neurons in a WTA circuit, as discussed by \citep[Ch.~5]{rumelhart1986parallel}, where each weight vector moves toward the cluster of inputs that the corresponding neuron will represen

Reviewer #3 (Remarks to the Author):

I co-reviewed this manuscript with one of the reviewers who provided the listed reports. This is part of the Nature Communications initiative to facilitate training in peer review and to provide appropriate recognition for Early Career Researchers who co-review manuscripts.

Reviewer #4 (Remarks to the Author):

I thank the authors for their responsiveness to my comments. I've threaded my responses (RC) to the author's responses (AR) below.

WRT "A new loss function for rank learning and the associated learning rule

RC: The loss function proposed in equation 4 ... It would be interesting to see a sensitivity analysis exploring these parameters.

AR: Thank you for pointing us to Kendall Tau loss and its upper bounded hinge loss form. The chapter in the book has a great insight why such a loss function is convex, which we believe is also applicable to our loss function. We have added a discussion after eq. (4) on ll 594. In addition, we added a parameter sensitivity analysis for δ in a new Section A.2 and Fig. S1 of the Supplement.

RC: Thank you for your response here. I concur re: convexity. I feel this matter has been completely addressed.

RC: It is also mentioned that the learning algorithm is only ever trained on consecutive pairs of objects in the ordering ... how that effects the estimated ranks in Figure 2.E.

AR: We thank the reviewer for this insightful suggestion. Following this comment, we have added additional simulations in which training pairs are sampled randomly from all possible object pairs. We now report both the corresponding learning curves in Fig. 2 D and E, and the resulting estimated ranks in Fig. 2F and Fig. S4. These new results show that training with random pairs leads to faster convergence, while the estimated ranks continue to exhibit the same characteristic S-shaped profile observed under adjacent-pair training. The latter implies that the qualitative structure of the learned rank representation is robust to the choice of training pairs.

RC: Thank you for these additional experiments. I agree with your conclusions.

AR: In the main experiments we deliberately restricted training to adjacent pairs because it provides an opportunity to probe directly the generalization capability, i.e., the transitive inference capability, of the model: we train on adjacent pairs and test it on non-adjacent pairs.

In contrast, when random pairs are used for both training and testing, the learning problem becomes easier for two reasons: (i) transitive inference is no longer required, since long-range relations are directly observed during training; and (ii) the training and test sets overlap. These issues are addressed in the new Section B.3 of the Supplement.

RC: Agreed, and I think it would be appropriate to state this more clearly around lines 168-171, in addition to your statement in the supplementary material. But I'll defer to the authors as to whether or not they think it would break the flow of the document to do so. Otherwise, I consider this matter fully addressed.

A: Thank you for this feedback. We have included the following discussion on ll 169:

By design, the main experiments in Fig. \ref{fig:figure2}D restrict training to adjacent pairs and evaluate the learned ranks on non-adjacent pairs. This setting provides a direct test of transitive inference, since the model has to infer long-range order relations that were never presented during training. We further show in Fig. \ref{fig:figure2}E that the Rank Learning Rule also learns correct ranks when order relations between randomly chosen pairs are provided during learning, including non-adjacent pairs. The faster convergence in this setting is expected, because long-range relations are then observed directly during training and the training and test pair distributions overlap. Thus, this experiment serves as a complementary control, whereas the adjacent-pair setting provides the main test of transitive inference. A more detailed analysis is provided in Supplementary Information Section \ref{sec:random_pairs}.

2. A proof of convergence for the rank learning algorithm

RC: Line 508: Explain why only finitely many updates are made for a_k < a_min.
AR: Done (in the proof, on ll 622).
RC: Thank you. Concern resolved.

RC: Line 514: Does a_k having the property that w_t a_k -> -\infty contradict observation 1?
AR: No, Observation 1 shows that a_k := a_min has this property.
RC: Apologies for misreading. Concern resolved.

RC: Line 515: If a_q is the next larger item (specify larger than which, l or k? Assumed l)

AR: larger than a_k is meant (added in the proof on ll 630)
RC: Thank you. Concern resolved.

RC: lines 516-518. a_r should be a_l? Are there some errors in the subscripts?
AR: The indices r and l are bound variables, that are bound by separate quantifiers, an “for all” quantifier in the preceding statement of Observation 3 and an existential quantifier in the proof on the lines to which the reviewer refers.
RC: Apologies for misreading. Concern resolved.

RC: Shouldn't \delta show up in this proof?
AR: Yes, and it does show up in the proof of Observation 3
RC: Apologies for misreading. Concern resolved.

RC: More generally, I wasn't certain ... when implementing the algorithm in neuromorphic hardware.
AR: We would like to thank the reviewer for the insightful remark on a link to the Perceptron Convergence Theorem. This does in fact provide an alternative proof of the Rank Convergence Theorem for the case where the margin δ has value 0, that we have added on ll 641.

RC: Noted, very nice description. This matter has been addressed.

AR: We would be happy to acknowledge the reviewer by name for pointing out this link

RC: I would be delighted to take the authors up on the offer, thank you. I'll reach out to the editor so they can communicate the name once the blind review process is completed.

A: We have also contacted the editor regarding the question how the contribution of the reviewer can be acknowledged in the paper.

3. An algorithm that is implementable in neuromorphic hardware.

RC: The authors assert that this learning rule can be implemented in neuromorphic hardware .. Successfully demonstrated, this will be a powerful result.

AR: We thank the reviewer for this suggestion, and the ... All computations rely on local traces and programmable synaptic plasticity that is supported by Loihi 2.

RC: Thank you for this comprehensive response. I consider this matter completely addressed.

4. The emergence of cognitive maps from multiple learned orderings

RC: I quite enjoyed this discussion ... in autonomous systems.

AR: Several models for constructing cognitive maps in abstract concept spaces have already been proposed, see e.g.

Stöckl, C., Yang, Y., & Maass, W. (2024). Local prediction-learning in high-dimensional spaces enables neural networks to plan. Nature Communications, 15(1), 2344.

Therefore we have addressed here only the case where the underlying variables are ranks of linear orders (as announced by the title of this section 2.4: „Emergence of cognitive maps from several learnt order relationships“).

RC: Point well taken. My concern is addressed.

RC: That said ... 2D coordinates from the learned ranks.

AR: We agree that the discussion of grid cells in section 4.3.2 could be better integrated with the rest of the text. To address this, we've added a new Section C to the Supplement, which contains a comparison between the learned 2D ranks and the PCA result of the grid-cell activations for each object in Fig. S5, as suggested by the reviewer.

RC: Thank you for this additional discussion. My concerns here are addressed.

RC: Lines 295-296 state ... for the proposed method.

AR: We did not claim, or want to claim, that this inference can only be carried out via the cognitive map that emerges in this model. But we are not aware of any previous model for the emergence of a cognitive map from learning two order relations over a set of items. Only in that sense our model is “unique”. We also made an effort to keep this section 2.4 short because we wanted to focus on other material.

RC: Point well taken. I am likewise unaware of a prior demonstration. My concerns here have been resolved.

5. Extension of the rank-learning algorithms to contextual learning.

RC: As with the spatial relationships ... are kept separate.

AR: Several brain mechanisms have been elucidated that keep different contexts separate. We have added on ll 383 a reference to a recent paper to provides insight into neural mechanisms for that:

Zhang, Y., Liu, Y., Hu, Y., Zhang, Z., Du, J., Cui, L., ... & Xu, N. L. (2025). Dendritic computation for rule-

based flexible categorization. bioRxiv, 2025-06.

RC: Thank you, my concerns here have been addressed.

RC: That said, reading this section ... but it warrants mentioning here.

AR: Thank you for raising this point. We have added a few sentences on ll 369 to clarify it. As shown in the new Section D.2 of the Supplementary Material ... all remaining items within each context at their correct relative rankings.

RC: Thank you for the comprehensive response. My concerns here have been addressed.

RC: How would this extend to 3 sets??

AR: The algorithm extends directly to three sets by assigning to each set a suitable context code $\mathbf{c}$. Learning proceeds in the same two-phase manner as in the two-set case. During the first phase, the model is trained only on adjacent item pairs within each context, resulting in three independently learned ranks. In the second phase, 2 boundary pairs linking the contexts are introduced, which is sufficient to merge the three sets into a single coherent ranking. The new Section D.3 and Figure S8 discuss the experimental setup and the learning process for the three-set case.

RC: Thank you for the response. My concerns here have been addressed. Additionally, apologies for the double question mark. It was a typo and not meant to imply incredulity.

RC: How would/could you deal with non-disjoint sets in different contexts

AR: An example of non-disjoint sets is already discussed in section 2.4: There the two sets on which different orders are defined in different context are in fact the same. When sets in different contexts are non-disjoint, our algorithm requires different contexts gating for different sub-groups of rank estimator neurons (as shown in Figure. 4).

RC: Apologies for misreading. My concerns were already addressed.

RC: Clearly, the much larger learning rate on the context is necessary to capture the effect of rapid learning. It would be valuable to produce a sensitivity analysis to the relative learning rates.

AR: We thank the reviewer for this suggestion and have added a sensitivity analysis of the relative

learning rates in a new Figure S7. Specifically, we systematically varied the ratio η_c / η and quantified learning success across repeated simulations. Success was defined as correctly linking the two ranks across contexts during the second phase using only the boundary pair, while preserving the correct ordering within each context and across contexts.

For each ratio, the success rate was averaged over 100 independent runs, each trained from scratch with different random seeds in both phase 1 (train short) and phase 2 (train long). We find that learning stability increases monotonically with the ratio η_c / η: the success rate rises rapidly as the ratio increases, approaches unity around $\eta_c / \eta \approx 10$, and becomes consistently successful (success rate =1) for ratios larger than approximately 20. These results confirm that a larger learning rate for the context pathway is critical for rapid and robust contextual integration and demonstrate that the effect is stable over a broad range of parameter values rather than relying on fine tuning.

RC: Thank you for the response to my concern here. I concur re: the robustness of the proposed model.

RC: Additionally, I would be interested in seeing how much longer it takes to learn the associations if no context was provided, and how that may compare to human learning.

AR: As far as we understand, it is impossible to learn several orders over the same items if no context is provided. Without context, the given information about the relative orders of individual pairs is highly contradictory.

RC: I don't know that that is strictly true, if one provided a ranking for the upper element from one set to the lower element of the other set. But this would basically be forcing the model to re-learn a global ranking, and it would likely follow the same process as learning from scratch, albeit with some inertia to over-come from the pre-training. That said, I think pursuing this matter would not add to the paper in any meaningful way. So I take the author's point, and consider my concerns resolved.

6. An explanation for the differences between the low-dimensional embeddings of models of biological data.

RC: 6. An explanation for the differences between the low-dimensional embeddings of models of biological data.

AR: We have shown in Section 2.6 that our model, as well as theory, provide the same parabolic shape of ... characterized in the review (Summerfield et al., 2020).

RC: Apologies. This was meant to be a section heading and not a comment that required a direct response. I concur with the statements the authors made in response.

RC: A) In figure 5 the tuning curves ... are likely to be small.

AR: Good point, we have added on ll 808 after eq. (20) a sentence to clarify this.

RC: Thank you. My concern here is addressed.

RC: B) In the main text, the motivation to approximate the covariance matrix by first the Toeplitz and then circulant matrices is pretty limited. It would improve the text if it were to say that the motivation for this approximation was to provide a tractable analysis of the low-dimensional projection that is not available directly from operating on the covariance matrix.
C) The description of how to compute a covariance matrix seems fairly straightforward, and reproducing it here (lines 629-635) is not a valuable use of space. Furthermore, as someone who frequently, albeit numerically, gets the eigevectors of covariance matrices, the reason why operating directly on the covariance matrix is not sufficient is not well motivated. What happens to the analysis in the following sections if the matrix does not have the banded symmetric Toeplitz structure? What happens if the circulant approximation is a bad approximation?

AR: We thank the reviewer for this suggestion and have compressed the section accordingly. The Toeplitz assumption captures the correlation structure induced by overlapping rank-selective tuning that leads to low-dimensional curvature, while the circulant approximation removes boundary effects and allows a closed-form characterization of the eigenvectors. When the covariance matrix deviates from
a banded symmetric Toeplitz form, eigenvectors must be computed numerically, but the low-dimensional ordered geometry persists with smooth distortions. When the circulant approximation is poor, boundary effects prevent exact cosine-form eigenvectors, yet the projected representations remain low-dimensional and curved. Clarifying sentences were added after Eqs. (26), (29), and (30), and the explicit description of covariance computation was removed.

RC: Thank you, the clarifying sentences were most beneficial. My concerns here have been addressed.

Minor notes

line 660: "(with arbitrary..." -> an open parenthesis that doesn't seem to have a matching close parenthesis.
line 1057-1057: Maybe cite section 17.2 of Schalev-Schwartz & Ben-David for the interested reader?

A: Thank you for the suggestion. We have fixed these points.

14.5.2026

Rebuttal to the 2nd review of Nature Communications manuscript NCOMMS-25-56574A by Yukun Yang and Wolfgang Maass

We would like to thank the reviewers for their helpful comments!

In this 2nd round two (optional) additions of comments were suggested by Reviewers #2 and #4, and two minor points were pointed out by Reviewer #4. We have implemented all of these suggestions, as detailed below.

REVIEWER COMMENTS

Reviewer #1 (Remarks to the Author):

The authors have done a good job addressing my comments. I'm happy to recommend acceptance.

Reviewer #2 (Remarks to the Author):

The authors have addressed most of my comments and revised the manuscript adequately. Therefore, I have only a minor comment regarding my previous comment 4, which was not clear enough:

On ll. 244-256: The authors compared the convergence of their learning rule with that of the Perceptron convergence theorem. However, the form of their Rank Learning Rule more resembles the classic Competitive Learning Rule, which also converges on the surface of a high-dimensional sphere due to weight renormalization following weight update. What is a surprise if the convergence of the Rank Learning Rule is compared with that of the Competitive Learning Rule?

The authors are correct."Competitive learning rule" is a learning rule for WTA circuits. In classic PDF literature, however, WTA circuits are often not modeled explicitly. Instead, feature detection through competition is modeled by a simple learning rule for feature-selective neurons, as in chapter 5 of the following textbook: https://gwern.net/doc/ai/nn/1986-rumelhart-pdp-v1.pdf

I observe some mathematical similarity between this and their learning rules. In their reply, the authors clarified that their Rank Learning Rule is supervised, whereas this competitive learning rule is unsupervised. It would be nice if the authors could comment on similarity and/or dissimilarity between the two rules. However, I do not insist on this point: the authors may include some comments in the manuscript if they consider such a comparison valuable.

A: Thank you for pointing out this similarity. We have addressed it on ll 240 by adding the following explanation:

The weight dynamics under the Rank Learning Rule can intuitively be understood as follows: The weight vector moves toward the higher ranked input vector and away from the lower ranked input vector. This is somewhat analogous to the competitive learning of neurons in a WTA circuit, as discussed by \citep[Ch.~5]{rumelhart1986parallel}, where each weight vector moves toward the cluster of inputs that the corresponding neuron will represen

Reviewer #3 (Remarks to the Author):

I co-reviewed this manuscript with one of the reviewers who provided the listed reports. This is part of the Nature Communications initiative to facilitate training in peer review and to provide appropriate recognition for Early Career Researchers who co-review manuscripts.

Reviewer #4 (Remarks to the Author):

I thank the authors for their responsiveness to my comments. I've threaded my responses (RC) to the author's responses (AR) below.

WRT "A new loss function for rank learning and the associated learning rule

RC: The loss function proposed in equation 4 ... It would be interesting to see a sensitivity analysis exploring these parameters.

AR: Thank you for pointing us to Kendall Tau loss and its upper bounded hinge loss form. The chapter in the book has a great insight why such a loss function is convex, which we believe is also applicable to our loss function. We have added a discussion after eq. (4) on ll 594. In addition, we added a parameter sensitivity analysis for δ in a new Section A.2 and Fig. S1 of the Supplement.

RC: Thank you for your response here. I concur re: convexity. I feel this matter has been completely addressed.

RC: It is also mentioned that the learning algorithm is only ever trained on consecutive pairs of objects in the ordering ... how that effects the estimated ranks in Figure 2.E.

AR: We thank the reviewer for this insightful suggestion. Following this comment, we have added additional simulations in which training pairs are sampled randomly from all possible object pairs. We now report both the corresponding learning curves in Fig. 2 D and E, and the resulting estimated ranks in Fig. 2F and Fig. S4. These new results show that training with random pairs leads to faster convergence, while the estimated ranks continue to exhibit the same characteristic S-shaped profile observed under adjacent-pair training. The latter implies that the qualitative structure of the learned rank representation is robust to the choice of training pairs.

RC: Thank you for these additional experiments. I agree with your conclusions.

AR: In the main experiments we deliberately restricted training to adjacent pairs because it provides an opportunity to probe directly the generalization capability, i.e., the transitive inference capability, of the model: we train on adjacent pairs and test it on non-adjacent pairs.

In contrast, when random pairs are used for both training and testing, the learning problem becomes easier for two reasons: (i) transitive inference is no longer required, since long-range relations are directly observed during training; and (ii) the training and test sets overlap. These issues are addressed in the new Section B.3 of the Supplement.

RC: Agreed, and I think it would be appropriate to state this more clearly around lines 168-171, in addition to your statement in the supplementary material. But I'll defer to the authors as to whether or not they think it would break the flow of the document to do so. Otherwise, I consider this matter fully addressed.

A: Thank you for this feedback. We have included the following discussion on ll 169:

By design, the main experiments in Fig. \ref{fig:figure2}D restrict training to adjacent pairs and evaluate the learned ranks on non-adjacent pairs. This setting provides a direct test of transitive inference, since the model has to infer long-range order relations that were never presented during training. We further show in Fig. \ref{fig:figure2}E that the Rank Learning Rule also learns correct ranks when order relations between randomly chosen pairs are provided during learning, including non-adjacent pairs. The faster convergence in this setting is expected, because long-range relations are then observed directly during training and the training and test pair distributions overlap. Thus, this experiment serves as a complementary control, whereas the adjacent-pair setting provides the main test of transitive inference. A more detailed analysis is provided in Supplementary Information Section \ref{sec:random_pairs}.

2. A proof of convergence for the rank learning algorithm

RC: Line 508: Explain why only finitely many updates are made for a_k < a_min.
AR: Done (in the proof, on ll 622).
RC: Thank you. Concern resolved.

RC: Line 514: Does a_k having the property that w_t a_k -> -\infty contradict observation 1?
AR: No, Observation 1 shows that a_k := a_min has this property.
RC: Apologies for misreading. Concern resolved.

RC: Line 515: If a_q is the next larger item (specify larger than which, l or k? Assumed l)

AR: larger than a_k is meant (added in the proof on ll 630)
RC: Thank you. Concern resolved.

RC: lines 516-518. a_r should be a_l? Are there some errors in the subscripts?
AR: The indices r and l are bound variables, that are bound by separate quantifiers, an “for all” quantifier in the preceding statement of Observation 3 and an existential quantifier in the proof on the lines to which the reviewer refers.
RC: Apologies for misreading. Concern resolved.

RC: Shouldn't \delta show up in this proof?
AR: Yes, and it does show up in the proof of Observation 3
RC: Apologies for misreading. Concern resolved.

RC: More generally, I wasn't certain ... when implementing the algorithm in neuromorphic hardware.
AR: We would like to thank the reviewer for the insightful remark on a link to the Perceptron Convergence Theorem. This does in fact provide an alternative proof of the Rank Convergence Theorem for the case where the margin δ has value 0, that we have added on ll 641.

RC: Noted, very nice description. This matter has been addressed.

AR: We would be happy to acknowledge the reviewer by name for pointing out this link

RC: I would be delighted to take the authors up on the offer, thank you. I'll reach out to the editor so they can communicate the name once the blind review process is completed.

A: We have also contacted the editor regarding the question how the contribution of the reviewer can be acknowledged in the paper.

3. An algorithm that is implementable in neuromorphic hardware.

RC: The authors assert that this learning rule can be implemented in neuromorphic hardware .. Successfully demonstrated, this will be a powerful result.

AR: We thank the reviewer for this suggestion, and the ... All computations rely on local traces and programmable synaptic plasticity that is supported by Loihi 2.

RC: Thank you for this comprehensive response. I consider this matter completely addressed.

4. The emergence of cognitive maps from multiple learned orderings

RC: I quite enjoyed this discussion ... in autonomous systems.

AR: Several models for constructing cognitive maps in abstract concept spaces have already been proposed, see e.g.

Stöckl, C., Yang, Y., & Maass, W. (2024). Local prediction-learning in high-dimensional spaces enables neural networks to plan. Nature Communications, 15(1), 2344.

Therefore we have addressed here only the case where the underlying variables are ranks of linear orders (as announced by the title of this section 2.4: „Emergence of cognitive maps from several learnt order relationships“).

RC: Point well taken. My concern is addressed.

RC: That said ... 2D coordinates from the learned ranks.

AR: We agree that the discussion of grid cells in section 4.3.2 could be better integrated with the rest of the text. To address this, we've added a new Section C to the Supplement, which contains a comparison between the learned 2D ranks and the PCA result of the grid-cell activations for each object in Fig. S5, as suggested by the reviewer.

RC: Thank you for this additional discussion. My concerns here are addressed.

RC: Lines 295-296 state ... for the proposed method.

AR: We did not claim, or want to claim, that this inference can only be carried out via the cognitive map that emerges in this model. But we are not aware of any previous model for the emergence of a cognitive map from learning two order relations over a set of items. Only in that sense our model is “unique”. We also made an effort to keep this section 2.4 short because we wanted to focus on other material.

RC: Point well taken. I am likewise unaware of a prior demonstration. My concerns here have been resolved.

5. Extension of the rank-learning algorithms to contextual learning.

RC: As with the spatial relationships ... are kept separate.

AR: Several brain mechanisms have been elucidated that keep different contexts separate. We have added on ll 383 a reference to a recent paper to provides insight into neural mechanisms for that:

Zhang, Y., Liu, Y., Hu, Y., Zhang, Z., Du, J., Cui, L., ... & Xu, N. L. (2025). Dendritic computation for rule-

based flexible categorization. bioRxiv, 2025-06.

RC: Thank you, my concerns here have been addressed.

RC: That said, reading this section ... but it warrants mentioning here.

AR: Thank you for raising this point. We have added a few sentences on ll 369 to clarify it. As shown in the new Section D.2 of the Supplementary Material ... all remaining items within each context at their correct relative rankings.

RC: Thank you for the comprehensive response. My concerns here have been addressed.

RC: How would this extend to 3 sets??

AR: The algorithm extends directly to three sets by assigning to each set a suitable context code $\mathbf{c}$. Learning proceeds in the same two-phase manner as in the two-set case. During the first phase, the model is trained only on adjacent item pairs within each context, resulting in three independently learned ranks. In the second phase, 2 boundary pairs linking the contexts are introduced, which is sufficient to merge the three sets into a single coherent ranking. The new Section D.3 and Figure S8 discuss the experimental setup and the learning process for the three-set case.

RC: Thank you for the response. My concerns here have been addressed. Additionally, apologies for the double question mark. It was a typo and not meant to imply incredulity.

RC: How would/could you deal with non-disjoint sets in different contexts

AR: An example of non-disjoint sets is already discussed in section 2.4: There the two sets on which different orders are defined in different context are in fact the same. When sets in different contexts are non-disjoint, our algorithm requires different contexts gating for different sub-groups of rank estimator neurons (as shown in Figure. 4).

RC: Apologies for misreading. My concerns were already addressed.

RC: Clearly, the much larger learning rate on the context is necessary to capture the effect of rapid learning. It would be valuable to produce a sensitivity analysis to the relative learning rates.

AR: We thank the reviewer for this suggestion and have added a sensitivity analysis of the relative

learning rates in a new Figure S7. Specifically, we systematically varied the ratio η_c / η and quantified learning success across repeated simulations. Success was defined as correctly linking the two ranks across contexts during the second phase using only the boundary pair, while preserving the correct ordering within each context and across contexts.

For each ratio, the success rate was averaged over 100 independent runs, each trained from scratch with different random seeds in both phase 1 (train short) and phase 2 (train long). We find that learning stability increases monotonically with the ratio η_c / η: the success rate rises rapidly as the ratio increases, approaches unity around $\eta\_c / \eta \approx 10$, and becomes consistently successful (success rate =1) for ratios larger than approximately 20. These results confirm that a larger learning rate for the context pathway is critical for rapid and robust contextual integration and demonstrate that the effect is stable over a broad range of parameter values rather than relying on fine tuning.

RC: Thank you for the response to my concern here. I concur re: the robustness of the proposed model.

RC: Additionally, I would be interested in seeing how much longer it takes to learn the associations if no context was provided, and how that may compare to human learning.

AR: As far as we understand, it is impossible to learn several orders over the same items if no context is provided. Without context, the given information about the relative orders of individual pairs is highly contradictory.

RC: I don't know that that is strictly true, if one provided a ranking for the upper element from one set to the lower element of the other set. But this would basically be forcing the model to re-learn a global ranking, and it would likely follow the same process as learning from scratch, albeit with some inertia to over-come from the pre-training. That said, I think pursuing this matter would not add to the paper in any meaningful way. So I take the author's point, and consider my concerns resolved.

6. An explanation for the differences between the low-dimensional embeddings of models of biological data.

RC: 6. An explanation for the differences between the low-dimensional embeddings of models of biological data.

AR: We have shown in Section 2.6 that our model, as well as theory, provide the same parabolic shape of ... characterized in the review (Summerfield et al., 2020).

RC: Apologies. This was meant to be a section heading and not a comment that required a direct response. I concur with the statements the authors made in response.

RC: A) In figure 5 the tuning curves ... are likely to be small.

AR: Good point, we have added on ll 808 after eq. (20) a sentence to clarify this.

RC: Thank you. My concern here is addressed.

RC: B) In the main text, the motivation to approximate the covariance matrix by first the Toeplitz and then circulant matrices is pretty limited. It would improve the text if it were to say that the motivation for this approximation was to provide a tractable analysis of the low-dimensional projection that is not available directly from operating on the covariance matrix.
C) The description of how to compute a covariance matrix seems fairly straightforward, and reproducing it here (lines 629-635) is not a valuable use of space. Furthermore, as someone who frequently, albeit numerically, gets the eigevectors of covariance matrices, the reason why operating directly on the covariance matrix is not sufficient is not well motivated. What happens to the analysis in the following sections if the matrix does not have the banded symmetric Toeplitz structure? What happens if the circulant approximation is a bad approximation?

AR: We thank the reviewer for this suggestion and have compressed the section accordingly. The Toeplitz assumption captures the correlation structure induced by overlapping rank-selective tuning that leads to low-dimensional curvature, while the circulant approximation removes boundary effects and allows a closed-form characterization of the eigenvectors. When the covariance matrix deviates from
a banded symmetric Toeplitz form, eigenvectors must be computed numerically, but the low-dimensional ordered geometry persists with smooth distortions. When the circulant approximation is poor, boundary effects prevent exact cosine-form eigenvectors, yet the projected representations remain low-dimensional and curved. Clarifying sentences were added after Eqs. (26), (29), and (30), and the explicit description of covariance computation was removed.

RC: Thank you, the clarifying sentences were most beneficial. My concerns here have been addressed.

Minor notes

line 660: "(with arbitrary..." -> an open parenthesis that doesn't seem to have a matching close parenthesis.
line 1057-1057: Maybe cite section 17.2 of Schalev-Schwartz & Ben-David for the interested reader?

A: Thank you for the suggestion. We have fixed these points.

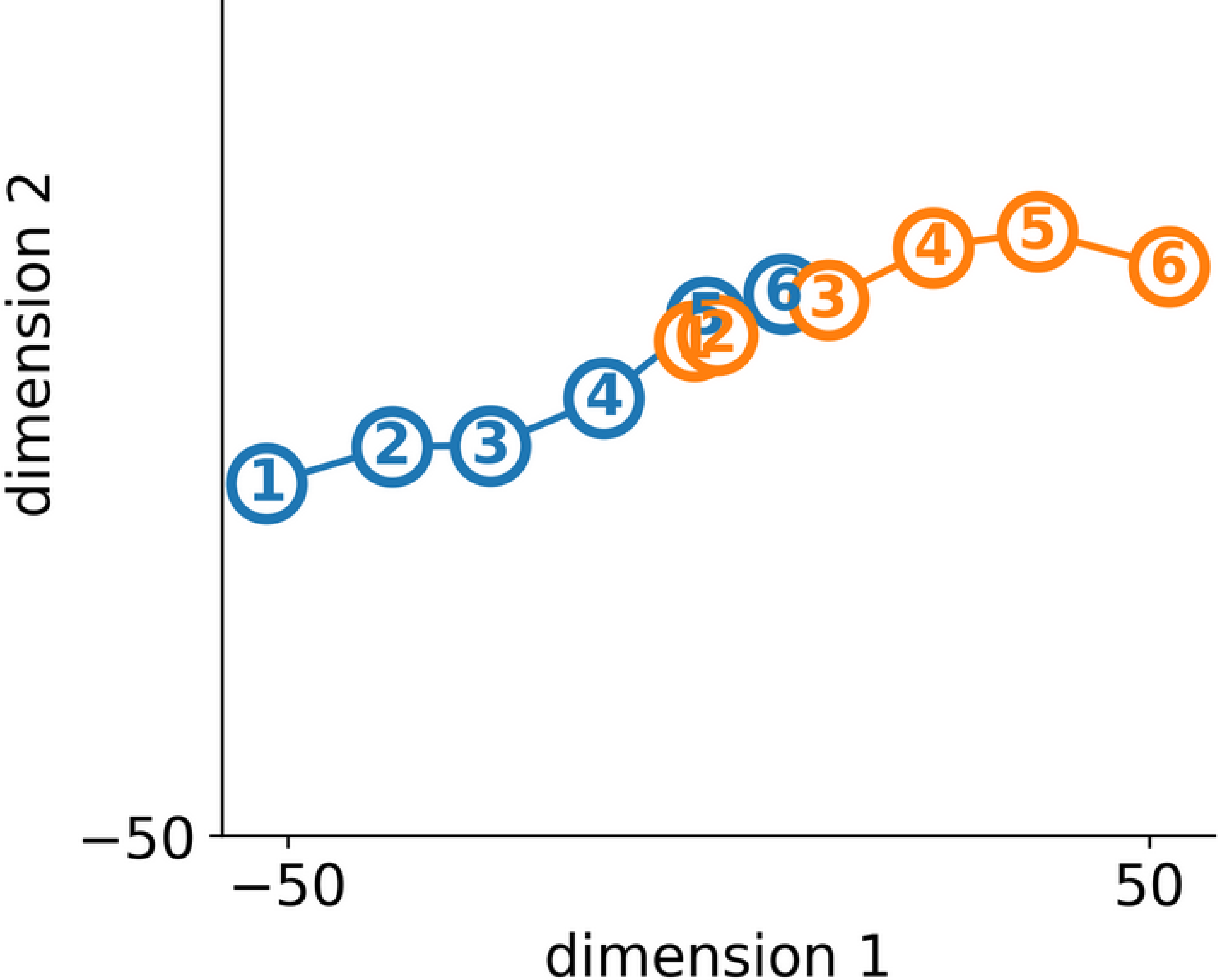

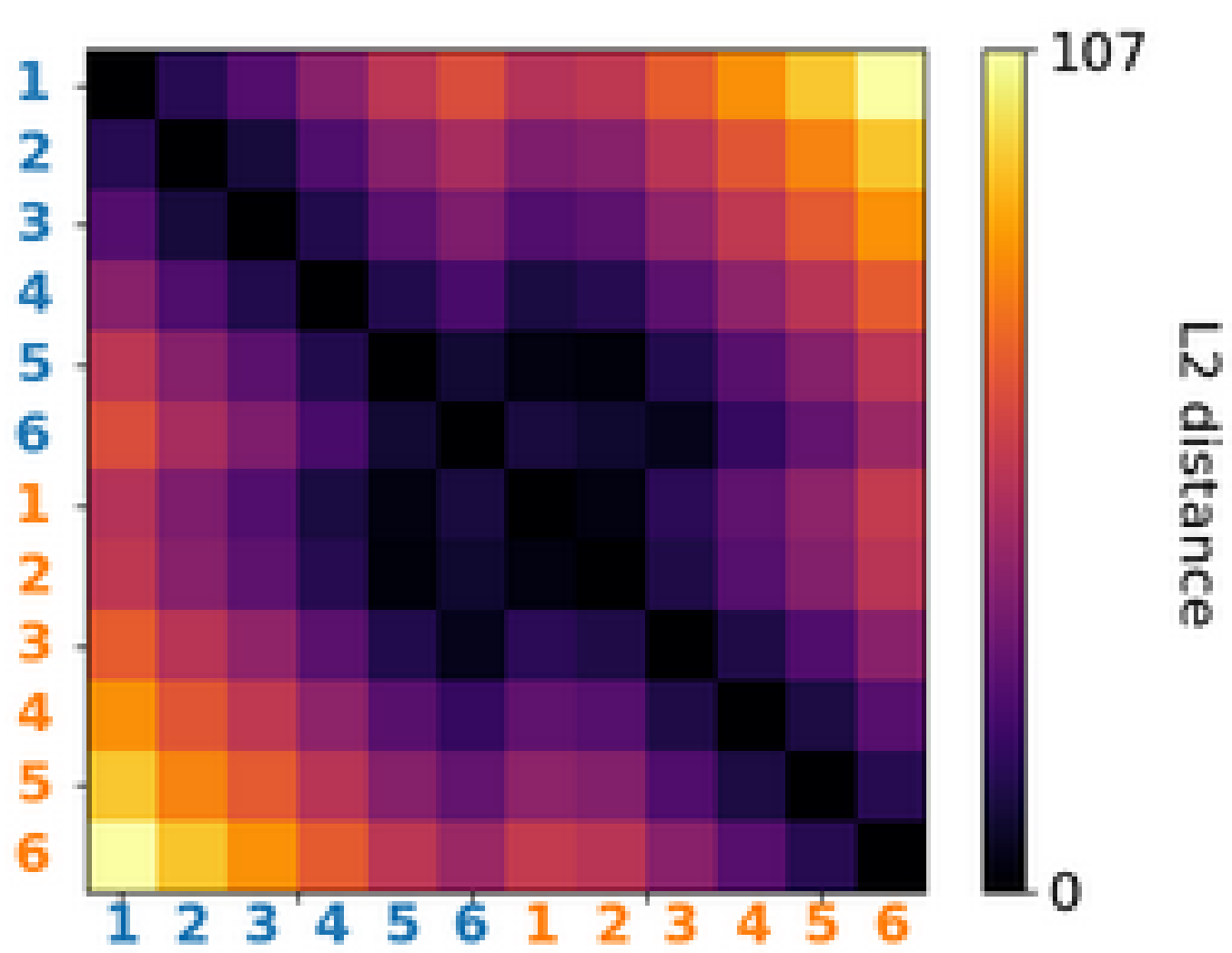

107.13577546766172

```
[5]: import torch
```
